# Diagnosis Value of Patient Evaluation Components Applicable in Primary Care Settings for the Diagnosis of Low Back Pain: A Scoping Review of Systematic Reviews

**DOI:** 10.3390/jcm12103581

**Published:** 2023-05-21

**Authors:** Janny Mathieu, Mégane Pasquier, Martin Descarreaux, Andrée-Anne Marchand

**Affiliations:** 1 Department of Anatomy, Université du Québec à Trois-Rivières, 3351, Boul. des Forges, C.P. 500, Trois-Rivieres, QC G8Z 4M3, Canada; 2 Institut Franco-Européen de Chiropraxie, 72 Chemin de la Flambère, 31300 Toulouse, France; mpasquier@ifec.net; 3 Department of Human Kinetics, Université du Québec à Trois-Rivières, 3351, Boul. des Forges, C.P. 500, Trois-Rivières, QC G8Z 4M3, Canada; martin.descarreaux@uqtr.ca; 4 Department of Chiropractic, Université du Québec à Trois-Rivières, 3351, Boul. des Forges, C.P. 500, Trois-Rivières, QC G8Z 4M3, Canada; andree-anne.marchand@uqtr.ca

**Keywords:** low back pain, diagnostic accuracy, history taking, physical examination, primary care

## Abstract

Low back pain ranks as the leading cause of years lived with disability worldwide. Although best practice guidelines share a consistent diagnostic approach for the evaluation of patients with low back pain, confusion remains as to what extent patient history and physical examination findings can inform management strategies. The aim of this study was to summarize evidence investigating the diagnostic value of patient evaluation components applicable in primary care settings for the diagnosis of low back pain. To this end, peer-reviewed systematic reviews were searched in MEDLINE, CINAHL, PsycINFO and Cochrane databases from 1 January 2000 to 10 April 2023. Paired reviewers independently reviewed all citations and articles using a two-phase screening process and independently extracted the data. Of the 2077 articles identified, 27 met the inclusion criteria, focusing on the diagnosis of lumbar spinal stenosis, radicular syndrome, non- specific low back pain and specific low back pain. Most patient evaluation components lack diagnostic accuracy for the diagnosis of low back pain when considered in isolation. Further research is needed to develop evidence-based and standardized evaluation procedures, especially for primary care settings where evidence is still scarce.

## 1. Introduction

Canada’s overall health expenditure is expected to account for 12.2% of its gross domestic product (GDP) in 2022 [1]. Increase use of health care services and compensation of health care providers are listed as the two main health care cost drivers, accounting for almost half of estimated Canada’s total health spending [1]. A cross-sectional analysis of administrative health data of the province of Ontario, Canada revealed that 1.6 million outpatient physician visits for spinal conditions, of which 86% occurred in primary care settings, were made in 2013–2014 [2]. Given its high prevalence, low back pain (LBP) accounts for a significant share of Canada’s health care spending. Compared to adults without back problems, patients with LPB present higher rates of health care utilization and costs, resulting approximately in 1.95 billion CAD in costs in 2019 [3].

Low back pain ranks as the leading cause of years lived with disability (YLDs) worldwide [4]. Although several global initiatives have been implemented to address the burden of LBP, YLDs attributable to this condition rose by 17.8% between 2007 and 2017 [4], reflecting its continuous burden increase as the population grows and ages. In the 2018 Lancet Low Back Pain Series, experts highlighted several potential measures to support healthcare systems in the prevention and management of disabling LBP [5]. Clinical and referral pathways redesign, and the integration of consistent evidence-based clinical care standards across healthcare systems and settings, were proposed to promote timely access to effective healthcare, while preventing the use of low-value care approaches [5].

Several evidence-based clinical guidelines [6,7,8,9,10,11,12] have been published over the years to provide healthcare providers with the best practice recommendations for the evaluation and management of patients with LBP. These guidelines share a consistent diagnostic approach based on a focused patient history and physical examination, which should assess level of concern for major structural or other pathologies, and the presence of co-morbidities and neurological signs. This approach should enable clinicians to identify the type of LBP (i.e., non-specific LBP, radicular syndrome, specific LBP), and help them determine whether the patient’s condition warrants further investigation or a referral to the appropriate healthcare provider. However, clinicians agree that such guideline recommendations may lack clarity and lead to confusion, as the rationale for diagnostic decisions and the diagnostic accuracy of endorsed clinical indicators are typically not provided [13,14]. This contributes to a widening gap between evidence and clinical practices [15,16,17] and leads to the use of inappropriate diagnostic and therapeutic approaches, including overuse of imaging and opioid prescriptions, as well as an increasing number of unnecessary referrals to medical specialists [16,18].

As the public health care system struggles with limited healthcare resources in the face of increasing demands for services, initial diagnostic accuracy is deemed crucial to enable patients to see the right professional at the right time, while precluding the use of ineffective and costly therapeutic approaches [5]. Thus, to assist healthcare providers in evidence-based decision making, there is a need to clearly define evaluation components and clinical indicators endorsed by practice guidelines for the diagnosis of LBP and to clarify to what extent these should inform clinical decisions. Therefore, the aim of this study was to summarize evidence investigating the diagnostic value of patient evaluation components applicable in primary care settings for the diagnosis of LBP.

## 2. Methods

### 2.1. Study Design

To address our research question, a scoping review was conducted based on the frameworks proposed by Arksey and O’Malley [19] and Levac et al. [20]. This type of study allows us to report on the current state of knowledge in a research field and captures the breadth of information on a topic that has been widely studied and for which the available data are numerous and heterogeneous [21]. Consistently with this framework, we did not appraise the methodological quality of the included studies.

### 2.2. Search Strategy

Our search strategy was developed by one of the authors (J.M.), and two coauthors (A.-A.M., M.D.) subsequently cross-validated the search to ensure completeness of results. The search strategy was developed in MEDLINE (see Supplementary File S1) and then adapted to other bibliographic databases. Search terms included controlled vocabulary for each database and free-text words for the key concepts of low back pain, diagnostic validity, patient evaluation, and systematic review. In addition, reference lists from relevant articles and previously published systematic reviews were hand-searched for additional potentially relevant reviews. We initially searched MEDLINE, CINAHL, PsycINFO and Cochrane databases from 1 January 2000 to 30 April 2022 and updated the search on 10 April 2023. EndNote was used to de-duplicate references electronically across all databases, record the number of duplicates identified and manage the search results.

### 2.3. Study Selection

#### 2.3.1. Inclusion Criteria

To be included, studies had to meet the following criteria: (1) written in the English or French languages; (2) systematic reviews of diagnostic studies that included comparative or exploratory studies, cross-sectional, cohort or case-control studies, or secondary analyses of randomized controlled trials; (3) focused on adults (aged > 18 years) suffering from any type of LBP with or without radiating pain; (4) investigated at least one index test (e.g., demographics, patient history and clinical examination findings) for the diagnosis of LBP, applicable in primary care settings; and (5) provided data on the diagnostic value of index tests. Study exclusion criteria included: primary studies, unpublished manuscripts, books and book chapters, conference proceedings, meeting and conference abstracts, thesis and dissertations, non-systematic reviews, laboratory studies, study not reporting on methodology and cadaveric or animal studies.

#### 2.3.2. Screening and Agreement

A two-phase (titles and abstracts; full-text articles) screening process was used to select eligible studies. In phase I screening, pairs of independent reviewers (J.M., M.P.) screened citation titles and abstracts to determine the eligibility of studies (categorizing studies as possibly relevant or irrelevant). In instances where eligibility could not be ascertained due to limited information in the title/abstract, the citation was considered ‘’possibly relevant” until a final decision was made upon full text review. Pairs of independent reviewers (J.M., M.P.) screened the full text of the “possibly relevant” during phase II screening to determine eligibility, and reasons for exclusion were documented. Reviewers met to discuss disagreements and to reach consensus in both phases. An additional reviewer (A.-A.M.) was involved if consensus could not be reached.

#### 2.3.3. Data Extraction

Data extraction forms were drafted and pilot-tested. Two reviewers (J.M., M.P.) independently extracted data and discussed to reconcile differences. A third reviewer (A.-A.M.) verified the extracted data to minimize error. Data extraction items included: first author’s name, publication year, country, number of included primary studies, details of search strategy, sample characteristics (e.g., size, mean age, LBP type, LBP duration), index tests and reference standards’ description, and diagnostic accuracy values (e.g., sensitivity and specificity) or measures (e.g., predictive values (PPV or NPV), likelihood ratios (LR), estimates of the summary receiver operating characteristic (ROC) curve, post-test probability (PPT), diagnostic odds ratios (DOR)). If a meta-analysis was conducted, reviewers extracted the meta-analytic summary of accuracy estimates across studies and its associated statistical uncertainty (e.g., 95% confidence intervals). Likelihood ratios were considered as the main clinical outcome measure for the purposes of this review and were clinically interpreted as outlined in Table 1.

### 2.4. Data synthesis and Analysis

A descriptive synthesis was conducted to provide details regarding the total number of studies kept for analysis, first author’s name and country, year of publication, settings of data collection, the number of primary studies included, and study populations’ characteristics (see Table 2).

To answer our research question, our review findings were sorted by LBP type (i.e., non-specific LBP, radicular syndrome, lumbar spinal stenosis, and specific LBP), and by evaluation component (i.e., demographics, patient history, and physical examination). This classification was chosen to facilitate the reporting of study results, as it reflected the LBP categories used in the eligible systematic reviews and in previously published practice guidelines.

## 3. Results

### 3.1. Descriptive Synthesis

**Figure 1:**
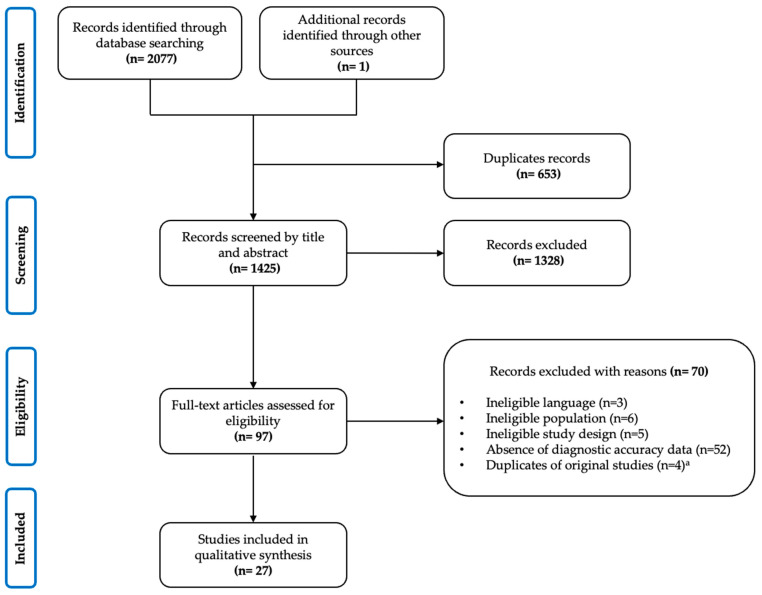
Flowchart diagram. ^a^ Excluded since they were part of a more recent systematic review.

The PRISMA flow diagram describing the process of review selection is presented in Figure 1. A total of 2077 articles were identified from the literature search. After duplicates were removed (*n* = 653), 1424 were screened by titles and abstracts and 1289 were deemed irrelevant. We reviewed full-text reports for the 96 systematic reviews of potential relevance, and of these, 69 were subsequently excluded, bringing the total count to 27 systematic reviews that were included in our analysis.

### 3.2. Characteristics of Included Reviews

**Figure 2:**
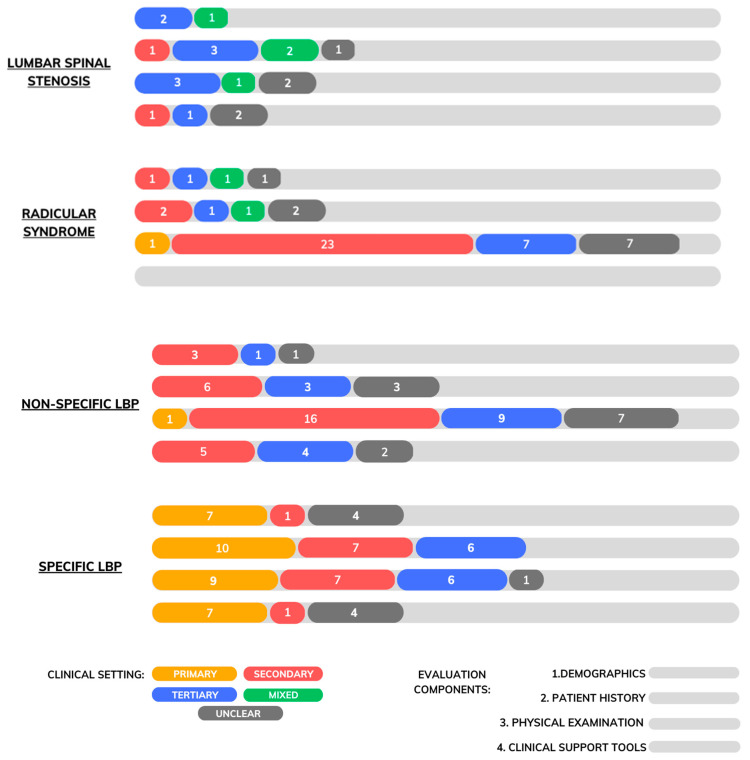
Distribution of studies by LBP type, evaluation component and care setting.

Included systematic reviews characteristics are presented in Table 1. The systematic reviews were published between 2000 and 2023, with 44.4% (12 of 27) of studies published between 2016 and 2023 and 40.7% (11 of 27) between 2010 and 2015. The total number of studies included in the reviews ranged from six to 62. Six systematic reviews performed meta-analyses. Overall, five systematic reviews were performed by researchers in Australia, four in the United Kingdom, three in the United-States, three in New Zealand, and the remaining were conducted in Canada (two), Germany (two), Italy (two), Singapore (one), Switzerland (one), Norway (one), and Kenya (one). Most reviews included various study designs such as prospective and retrospective cohort studies, case-control, and cross-sectional studies, and combined data obtained from various health care settings. Two reviews focused solely on the diagnostic accuracy of demographic, history or physical examination findings for the assessment of (1) lumbar spinal stenosis, six focused on (2) the radicular syndrome, 10 on (3) non-specific low back pain and seven on (4) specific low back pain. Two reviews [47,48] presented diagnostic accuracy data from each of the four categories. Figure 2 illustrates the distribution of studies by LBP type, evaluation component and care setting.

### 3.3. Lumbar Spinal Stenosis

Three systematic reviews [22,23,48] investigated the diagnostic accuracy of demographic, patient history or physical examination findings used to diagnose lumbar spinal stenosis [49]. A total of 12 primary studies were included in the systematic reviews, including eight (66.7%) prospective studies, three (25.0%) cross-sectional studies and one (8.3%) retrospective cohort study. Primary studies were mostly conducted in tertiary (33.3%) or secondary (25.0%) care settings. Two studies provided data from both primary and secondary health care settings. The studies’ sample size varied from 23 to 32,086 participants, with participants’ mean age ranging from 46.39 to 68.2 years old. Study populations differed, although participants were mostly described as adults with low back pain of any duration, with or without lower-extremity symptoms and with a suspicion of LSS. Of all primary studies, seven (58.3%) used a clinical reference standard (i.e., expert opinion based on clinical findings and imaging and/or surgery), whereas five studies relied solely on imaging and/or surgery findings to diagnose LSS. Imaging procedures included magnetic resonance imaging (MRI), computed tomography (CT) and radiography.

#### 3.3.1. Demographics

Supplementary Table S1 presents data on the two systematic reviews [22,23] that examined the diagnostic accuracy of demographic findings used to diagnose LSS. Neither of these systematic reviews conducted a meta-analysis. Of the three primary studies included in these reviews, two were conducted in a tertiary care setting [50,51], while one included data from both primary and secondary care settings [52]. All three primary studies only assessed the diagnostic value of patient age for the diagnosis of LSS but used a different age threshold. Overall, the criterion of being older (i.e., >48 years, >65 years or >70 years) seemed more sensitive than specific, with sensitivity values ranging from 0.64–0.88. Cook et al. [50] identified a 26% decrease in post-test probability when patient age was 48 years or younger. These findings are consistent with the pathophysiology of acquired LSS, which is known to be a degenerative process whose prevalence increases with age.

#### 3.3.2. Patient History

Three systematic reviews [22,23,48], including eight primary studies, provided data on the diagnostic value of patient history findings for the diagnosis of LSS (see Table S2). Neither of these systematic reviews pooled data statistically. Index tests identified were consistent with pain location characteristics (*n* = 15), relieving factors (*n* = 12), exacerbating factors (*n* = 11), subjective neurological symptoms (*n* = 7), pain character (*n* = 2), pain duration (*n* = 1) and functional limitations (*n* = 1). The absence of pain when seated (LR+ 7.21 [1.82, 28.61]; +PTP absolute difference 33.31%) [51] and the improvement of symptoms when bending forward (LR+ 1.41–6.4; +PTP absolute difference: 25.12%) [51,52,53] consistently presented the highest diagnostic utility for ruling in the diagnosis of LSS, generally reflected by higher positive likelihood ratios or post-test probability increases. Larger-magnitude negative likelihood ratios and post-test probability decreases were attributed to the absence of lower-extremity symptoms (LR− 0.34 [0.13, 0.88] −0.71 [0.46, 1.09]; −PTP absolute difference: 11.48–25.98%) [50,51] and to the absence of pain exacerbation when standing up or walking (LR− 0.33–0.97; −PTP absolute difference: 16.05%) [50,51,52,53,54], indicating that these index tests were generally the most clinically useful to rule out LSS. Although promising, none of these clinical tests seemed to perform strongly enough to justify using them as stands-alone for the diagnosis of LSS. Discrepancies in LSS definitions, reference standards and primary study methodologies may limit the generalizability of study findings.

#### 3.3.3. Physical Examination

Two systematic reviews [22,23], including six primary studies, investigated the diagnostic accuracy of physical examination findings for the diagnosis of LSS (see Table S3). Neither of these systematic reviews performed a meta-analysis. Functional and neurological symptoms change after level walking, and neurological examination findings were the most frequently investigated index tests. The absence of any functional neurological changes and no prolonged recovery after a two-level treadmill test [53,55] were consistently both associated with higher post-test probability decreases, ranging from 7.26 to 35%, reflective of better utility to rule out LSS. Neurological examination findings (i.e., absent Achilles reflex, sensory deficit, muscle weakness, abnormal Romberg and poor balance) [51] showed very small-magnitude positive likelihood ratios (1.49 CI 95% [0.98, 2.26] −4.06 CI 95% [1.29, 12.76]) and moderate to large positive post-test probability differences (9.35–27.17%). This suggests that the presence of neurological deficits might be used for ruling in the diagnosis of LSS. There is limited and highly inconsistent evidence to support the use of the Lumbar Extension Test and of the Straight Leg Raising Test for the diagnosis of LSS [51,52,56].

#### 3.3.4. Diagnostic Support Tools

Two systematic reviews [23,47], including four primary studies, provided data on the diagnostic value of diagnostic support tools for the diagnosis of LSS (see Table S4). Three diagnostic support tools were identified. Cook et al. [50] provided data on a five-item support tool, which improved post-test probabilities to 63–76% when from three to five of five conditions were met (i.e., (1) bilateral symptoms, (2) leg pain worse than back pain, (3) pain during walking/standing, (4) pain relief upon sitting, and (5) age > 48 years). Two studies [52,57] assessed the diagnostic value of a scoring system that involved age, self-reported symptoms and physical examination findings. Scores ≥ 7 were associated with very small-magnitude positive likelihood ratios (LR+ 1.6). Finally, Sugokia et al. [54] investigated the diagnostic performance of a clinical prediction rule that combined seven clinical findings (i.e., older age, duration of symptoms >6 months, improvement of symptoms when bending forward, no improvement of symptoms when bending backward, occurrence of symptoms when standing up, symptoms occurring when walking are improved by resting, and urinary incontinence). Scores ≥ 5 were associated with very small-magnitude positive likelihood ratios (LR+ 1.5 [1.1, 2.1]).

### 3.4. Radicular Syndrome

Seven systematic reviews [24,25,26,27,28,29,48] investigated the diagnostic accuracy of demographic, patient history or physical examination findings used to diagnose radicular syndromes. Three of the seven systematic reviews performed a meta-analysis [26,28,29]. Four systematic reviews presented data from both primary and secondary care settings, while three reviews included only studies from secondary or tertiary care environments. A total of 74 primary studies were included in these systematic reviews. The studies’ sample size varied from 16 to 2504 participants, with participants mean age ranging from 38.0 to 60.0 years old. Study populations were generally consistent, and most included adults with clinical signs and symptoms of any duration, suggestive of lumbar radiculopathy. Imaging procedures (i.e., MRI, CT, electromyography, radiography or myelography) and surgical findings were used as reference standards.

#### 3.4.1. Demographics

Two systematic reviews [24,48] examined the diagnostic accuracy of demographic findings used to diagnose radicular syndromes (see Table S5). Given the heterogeneity of the four included primary studies [58,59,60,61], meta-analyses were not performed. Demographic index tests included age, sex, living situation, education level and job type. None of these index tests significantly alter the likelihood of the condition (i.e., +LR ≥ 2; −LR ≤ 0.5), all reporting diagnostic odds ratios of less than 4.

#### 3.4.2. Patient History

Two systematic reviews [24,48], including six primary studies [58,59,60,61,62,63], investigated the diagnostic accuracy of patient history findings for the diagnosis of radicular syndromes (see Table S6). Mistry et al. provided data on the diagnostic utility of patient history findings to identify neuropathic pain in patients with low back-related leg pain. As stand-alone findings, duration and location of pain, pain history, subjective neurological symptoms and aggravating factors (i.e., coughing, sneezing, straining, sitting) appeared uninformative. Shultz et al. [48] provided data on the diagnostic accuracy of history-taking findings used to identify spinal conditions that cause low back-related leg pain. The diagnostic accuracy of 28 patient history items was reported, the most common categories corresponding to comorbidities and health history findings (*n* = 10; 35.7%), pain location characteristics (*n* = 4; 14.3%), pain duration (*n* = 4; 14.3%) and subjective neurological symptoms (*n* = 4; 14.3). For the diagnosis of lumbar radiculopathy, dermatomal distribution of pain presented the highest diagnostic performance [58,63], reflected by large-magnitude diagnostic odds ratios [DOR 24.29 (CI 95% 6.33, 93.19); 4.1 (CI 95% 2.20, 7.80)], followed by history of nerve injury [DOR 12.64 (CI 95% 3.59, 44.49)] [63]. When considered independently, other history-taking items did not significantly alter the probability of the condition (DOR < 4).

#### 3.4.3. Physical Examination

Six systematic reviews [24,25,26,27,28,29], 3 of which conducted a meta-analysis [26,28,29], provided data on the diagnostic accuracy of physical examination findings for the diagnosis of lumbar radiculopathy (see Table S7). Neurological examination findings (i.e., sensory deficits, motor deficits, impaired reflexes) and neurodynamic tests (i.e., Straight Leg raise (SLR), Crossed SLR, Slump test) were the most frequently investigated index tests. The overall findings revealed limited diagnostic accuracy of all components of the neurological examination when used in isolation to detect a nerve root compression or a disc herniation in patients with suspected radiculopathy, expressed by poor-to-moderate positive likelihood ratios. The highest specificity values attributed to neurological examination components, however, indicate that these tests might be useful to rule in the diagnosis of lumbar radiculopathy when used in combination. Based upon the current evidence, the SLR and Crossed SLR neurodynamic tests lack diagnostic utility as stand-alone findings, as suggested by variable diagnostic accuracy values. In surgical populations, the SLR and Crossed SLR tests, respectively, showed high sensitivity and moderate-to-high specificity but demonstrated poor diagnostic performance when imaging findings were used as a reference standard [28,29].

### 3.5. Non-Specific Low Back Pain

Ten systematic reviews [30,31,32,33,34,35,36,37,38,39], including 32 primary studies, investigated the diagnostic accuracy of demographic, patient history and physical examination findings commonly used in the diagnosis of non-specific low back pain. Only one systematic review performed a meta-analysis [30]. All systematic reviews presented data from mixed-care settings, the majority (17 of 30 primary studies) being from secondary care environments. The studies’ sample size varied from 21 to 337 participants, with participants’ mean age ranging from 38.4 to 62.04 years old. Study populations varied but mostly included adult patients with chronic LBP, without leg symptoms and neurological deficits. Three systematic reviews [34,35,37] focused specifically on populations with LBP presenting with suspected spondylolysis or spondylolisthesis, also referred to as lumbar instability.

#### 3.5.1. Demographics

Three systematic reviews [33,34,38], including five primary studies [64,65,66,67,68] provided data on the diagnostic accuracy of demographics for the diagnosis of non-specific LBP (see Table S8). All data were derived from secondary or tertiary care settings. Demographics investigated included older age (>65 or >50 years old), male gender, work status and the body mass index (BMI). As stand-alone findings, all demographic variables presented low-to-moderate sensitivity (Se range: 0.19 CI 95% [0.10, 0.31]–0.39 CI 95% [0.27, 0.52]) and moderate-to-high specificity values (Sp range: 0.56 CI 95% [0.44, 0.67]–0.85 CI 95% [0.78, 0.91]). The criterion of being older did not meet the threshold to alter the likelihood of the condition (+LR range: 0.6 CI 95% [0.3–1.1]–1.8 CI 95% [ 0.7, 4.7]); (−LR range: 0.78 CI 95% [0.49, 1.23]–1.21 CI 95% [0.98, 1.51]), with all studies reporting diagnostic odds ratios of less than 4. Overall, the study findings revealed that demographics had no value in diagnosing non-specific low back pain.

#### 3.5.2. Patient History

Five systematic reviews [30,33,34,36,38], including 12 primary studies [64,65,66,67,68,69,70,71,72,73,74,75], examined the diagnostic value of patient history findings for the diagnosis of non-specific LBP (see Table S9). Three systematic reviews [30,33,38] provided data on the diagnostic accuracy of patient history findings used to diagnose LBP originating from the facet joints, using single or double zygapophyseal diagnostic nerve blocks as reference standards. Most investigated index tests for this category were pain in the paraspinal area with or without leg pain, pain reduced with recumbency, and pain not increased with cough. The review by Sivayogam et al. [36] focused on the diagnostic performance of pain location characteristics (i.e., pain over groin, buttock, posterior superior iliac crest) to identify the sacroiliac joint (SIJ) as the source of pain, using SIJ blocks as reference standards. Finally, Grodahl et al. [34] provided data on the utility of patient history findings (i.e, age, male gender) to detect lumbar instability in patients with LBP. All these systematic reviews presented considerable heterogeneity in study populations, index tests’ descriptions and diagnostic accuracy data. Furthermore, most primary studies only reported sensitivity and specificity values of index tests, limiting the interpretability of study findings. Overall, evidence regarding the diagnostic accuracy of patient history components as stand-alone findings for the diagnosis of non-specific LBP was poor.

#### 3.5.3. Physical Examination

Ten systematic reviews [30,31,32,33,34,35,36,37,38,39], including 31 primary studies, provided data on the diagnostic accuracy of physical examination findings used for the diagnosis of non-specific LBP (see Table S10). Most of the systematic reviews investigated physical examination components including manual palpation, segmental motion testing, lumbar spine range of motion, and spinal orthopaedic testing. The meta-analysis performed by Han et al. [30] demonstrated informative LR+ for the distraction test (2.18; 95% CI [1.08–4.38]) to detect LBP originating from the SIJ, but uninformative LR− (0.73; 95% CI [0.54–0.99]). Absence of midline low back pain also demonstrated informative LR+ (2.41 95% CI [1.89–3.07]) and −LR 0.35 (95% CI [0.12–1.01]). Three studies [76,77,78], providing diagnostic accuracy data on static joint, joint motion and soft tissue palpation, were identified by Nolet et al. [31]. Little and inconsistent evidence was available to support the clinical usefulness of manual palpation when examining patients with LBP. When considered separately, Revel’s criteria (i.e., [1] age over 65 years, [2] pain well relieved by recumbency, and pain not exacerbated by [3] coughing, [4] forward flexion, [5] extension, [6] rising from flexion and [7] extension-rotation) also presented highly inconsistent performance in diagnosing LBP of facet joint origin, with sensitivity values ranging from 0.15 (95% CI 0.09–0.25) to 1.00 (95% CI 0.77–1.00) and specificity values ranging from 0.13 (95% CI 0.08–0.20) to 0.86 (95% CI 0.79–0.91) [33]. Overall, clinical provocation tests exhibited poor diagnostic value to identify LBP originating from the facet joints or the SIJ when used in isolation [36,39].

As for detecting lumbar spine instability, similar conclusions were drawn from four systematic reviews [32,34,35,37] examining the validity of passive segmental motion testing, manual palpation (e.g., hamstring muscle spasm, paravertebral tenderness, lumbar spinous process palpation) aberrant movements, spinal orthopaedic tests (e.g., one leg hyperextension test, prone instability test, instability catch sign, apprehension sign, sit-to-stand test) and neurodynamic tests (e.g., SLR, Active SLR, femoral stretch test), using flexion-extension radiographs as a reference standard. Almost all clinical tests were found to have poor diagnostic accuracy. Step deformity palpation, investigated in three studies [72,79,80], was the only test that showed promising diagnostic value to detect spondylolisthesis, with moderate-high sensitivity (Se range: 81–88) and high specificity values (Sp range: 87–100). Conclusions are, however, limited by studies’ risk of bias.

#### 3.5.4. Diagnostic Support Tools

Four systematic reviews [30,33,36,38], including 10 primary studies [65,66,68,74,81,82,83,84,85,86], provided data on the value of diagnostic support tools for the diagnosis of non-specific LBP (Table S11). Four primary diagnostic studies [66,68,74,82] evaluated the diagnostic performance of combined Revels’ criteria (positive with five or more clinical characteristics) to identify facet joints as the source of LBP. Due to clinical heterogeneity, the evidence for the diagnostic accuracy of combined Revels’ criteria was inconclusive, with sensitivity values ranging from 0.11 (95% CI 0.02–0.29) to 1.00 (95% CI 0.75–1.00), and the specificity values ranging from 0.66 (95% CI 0.46–0.82) to 0.91 (95% CI 0.83–0.96). Systematic reviews by Han et al. [30], Sivayogam et al. [36] and Hancock et al. [38] investigated the performance of various composites of pain provocation tests to diagnose LBP originating from the SIJ. Most studies suggested that a cutoff point of three or more positive responses from six provocation tests (i.e., distraction, compression, thigh thrust, sacral thrust and Gaenslen’s test) should be considered for clinical diagnosis of SIJ pain. Data pooling from six primary studies demonstrated informative LR+s (2.44 CI 95% [1.50, 3.98]) and LRs− (0.31 CI 95% [0.21, 0.47]) [30]. Heterogeneity in populations, reference standards and care settings may, however, limit the generalizability of study findings.

### 3.6. Specific Low Back Pain

Seven systematic reviews [40,41,42,43,44,45,46], of which two conducted a meta-analysis [40,43], investigated the diagnostic accuracy of demographic, patient history or physical examination findings used to diagnose specific LBP. Two systematic reviews [40,43] specifically focused on the diagnostic performance of clinical characteristics to screen for cauda equina syndrome (CES), two reviews focused on spinal fractures [44,46], and one review focused on spinal malignancy [45]. The systematic reviews by Maselli et al. [42] and Galliker et al. [41] evaluated the diagnostic accuracy of red flags for the diagnosis of any serious pathologies. A total of 72 primary studies were included in systematic reviews. The studies’ sample size varied from 31 to 2975 participants, with participants mean age ranging from 40.5 to 56.0 years old.

#### 3.6.1. Demographics

Cauda Equina Syndrome

One primary study [87] provided data on the diagnostic value of demographics used to diagnose CES (see Table S12). Being older than 55 years of age was identified as a potential valuable red flag to detect CES but presented highly inconsistent positive likelihood ratios values (+LR 1.5–8), thereby calling into question its diagnostic utility.

Spinal Fracture

Two systematic reviews [42,44], including six primary studies [87,88,89,90,91,92] investigated the diagnostic performance of demographic characteristics for the diagnosis of spinal fracture (see Table S13). Demographics investigated included age, gender and BMI. “Older age” at five different cut-offs was reported in four primary studies conducted in primary care [87,88,89,90]. Comparing the positive likelihood ratios at different cut-offs, “age greater than 70 years” (LR+ range: 3.1 95% CI [2.0, 4.7]–11.19 95% CI [5.33, 23.51]) and “age greater than 74 years” (LR+ range: 3.69 95% CI [3.00, 4.53]–9.39 95% CI [2.69, 32.75]) were identified as clinically informative, both resulting in a small-to-large increase in the likelihood of spinal fracture. Combining age and female gender revealed larger increases in positive likelihood ratios (LR+ range 14.59 95% CI [8.00, 26.61]–16.17 95% CI [4.47, 58.43]), indicating higher suspicion of spinal fracture. One study [92] conducted in a secondary care setting investigated the diagnostic accuracy of “BMI < 23”, which revealed a small increase (LR+ 2.3 95% CI [1.4, 3.4]) in the likelihood of fracture.

Malignancy

Two systematic reviews [42,45], including six primary studies [88,90,91,93,94,95] provided data on the diagnostic value of demographics for the diagnosis of spinal malignancy (see Table S14). The most reported index test was “Age greater than 50 years”, being investigated by five primary studies. Within the four primary care studies [88,90,93,94], the specificity of this index test ranged from 0.66 95% CI (0.63, 0.69) to 0.74 95% CI (0.70, 0.78), and the post-test probability for spinal malignancy following a positive screening test result was 0.8%.

Any Serious Spinal Pathologies

One primary study [96] provided data on the diagnostic accuracy of demographics used to diagnose any serious spinal pathologies (see Table S15). Being older than 70 years was the only index test investigated, presenting a very small-magnitude likelihood ratio (1.9 CI 95% [1.3, 2.8]).

#### 3.6.2. Patient History

Cauda Equina Syndrome

Two systematic reviews [42,43], including eight primary studies [91,92,97,98,99,100,101,102], investigated the diagnostic performance of patient history findings for the diagnosis of CES (see Table S16). Most studies (seven out of eight) were conducted in secondary and tertiary care settings. Bowel incontinence, urinary retention, urinary incontinence, leg pain and back pain were the most common signs and symptoms evaluated for their diagnostic accuracy in predicting CES against MRI. Dionne et al. [43] showed that all five clinical findings presented high pooled specificity values, ranging from 0.30 95% CI (0.23, 0.37) for back pain to 0.86 CI 95% (0.80, 0.91) for bowel incontinence, meaning these tests could be clinically useful for ruling in CES. However, pooled positive likelihood ratios, ranging from 0.80 95% CI (0.56, 1.14) for urinary incontinence to 1.60 95% CI (0.65, 3.94) for bowel incontinence suggested that a positive result in either of these tests leads to a very small shift in likelihood of CES when used in isolation.

Spinal Fracture

Three systematic reviews [42,44,46], including 11 primary studies [88,89,90,91,92,102,103,104,105,106,107] provided data on the diagnostic accuracy of patient history findings for the diagnosis of spinal fracture (see Table S17). In primary care studies [88,89,90,107], “history of trauma” was the most common red flag reported, presenting positive likelihood ratios ranging from 3.97 CI 95% (0.20, 79.15) to 12.85 CI 95% (8.58, 19.24), resulting in a small-to-large increase in the likelihood of spinal fracture. Three primary care studies [88,89,90] investigated the diagnostic value of prolonged corticosteroid use. This clinical finding was found to be highly specific (0.93 CI 95% [0.91, 0.95]–1.00), but yielded imprecise positive likelihood ratios between studies (2.5 CI 95% [1.1, 5.3]–48.5 CI 95% [11.62, 165.22]. When used in isolation, other history-taking items were not clinically informative for the diagnosis of spinal fracture.

Malignancy

Two systematic reviews [42,45], including eight primary studies [50,88,90,91,93,102,106,108], investigated the diagnostic accuracy of patient history findings for the diagnosis of spinal malignancy (see Table S18). The most common index tests evaluated were “previous history of cancer”, “no improvement in pain after one month”, “unexplained weight loss” and “insidious onset”. All clinical findings appeared more specific than sensitive across studies, resulting in very small-to-moderate increases in the likelihood of spinal malignancy. When used in isolation, only a “previous history of malignancy” significantly increased the post-test probability of spinal malignancy (LR+ 7.25 CI 95% 5.65, 9.3) in patients presenting with LBP [91].

Spinal Infection

Two systematic reviews [41,42], including two primary studies [91,109], provided data on the diagnostic accuracy of patient history findings for the diagnosis of spinal infection (see Table S19). According to one primary study conducted in a secondary care setting [91], a recent history of infection represented the most valuable red flag to detect spinal infection in patients presenting with LBP (LR+ 9.31 CI 95% 6.63, 13.07). When considered in isolation, the presence of night sweats and chills did not significantly increase the post-test probability of spinal infection, reflected by LR+ of less than 2. For the diagnosis of epidural abscess, Maselli et al. [42] found a moderate-to-large LR+ for intravenous drug use (13.7 CI 95% [11.4, 16.5]), a patient who was immunocompromised (5.1 CI 95% [3.2, 8.0]), indwelling vascular catheter (15.7 CI 95% [7.9, 31.0]) and other infection site (13.7 CI 95% [9.4, 19.8]).

Any Serious Spinal Pathologies

Two systematic reviews [41,42], including two primary studies [96,110], investigated the diagnostic accuracy of patient history findings for the diagnosis of serious spinal pathology (see Table S20). Overall, Maselli et al. [42] reported the diagnostic accuracy of 36 red flags, while Galliker et al. [41] provided data on 84 red flags for 12 serious spinal pathologies based on 10 primary studies. Current anticoagulants use (LR+ 6.4 CI 95% [2.6, 15.7]–7.0 CI 95% [1.9, 26.0]) and acute urinary retention (LR+ 2.0 CI 95% [0.6, 6.0]–8.7 CI 95% [3.1, 24.4]) were identified as clinically informative for the diagnosis of serious spinal pathology in both reviews. Other clinical findings did not significantly alter the post-test probability of serious spinal pathology when used in isolation.

#### 3.6.3. Physical Examination

Cauda Equina Syndrome

Three systematic reviews [40,42,43], including seven primary studies [97,99,100,101,111,112,113], provided data on the diagnostic accuracy of physical examination findings for the diagnosis of CES (see Table S21). All studies included adult patients presenting to secondary or tertiary care settings with acute CES, in which digital rectal examination (DRE) was the index test and lumbar MRI was the reference standard. Five studies provided data on the evaluation of anal tone that could be combined in meta-analysis. Tabrah et al. [40] found a very small pooled LR+ of 1.32 CI 95% (0.94, 1.66) and a high LR− ratio of 1.09 CI 95% (0.94, 1.26), both reflecting low diagnostic accuracy of DRE of anal tone in diagnosing CES. Based on four primary studies, Dionne et al. [43] found a very small pooled LR+ of 1.73 CI 95% (0.98, 3.08) for the presence of saddle anesthesia when used in isolation. Likelihood ratios attributed to the examination of internal anal sensation, anal squeeze and anal reflexes were not presented, although these clinical tests showed generally higher specificity than sensitivity values, suggesting these would be more clinically useful to rule in CES [40,42].

Spinal Fracture

Three systematic reviews [42,44,46], including 11 primary studies [89,90,92,103,104,105,106,107,114,115,116], investigated the diagnostic accuracy of physical examination findings for the diagnosis of spinal fracture (see Table S22). Most primary studies (eight out of 11) were conducted in secondary or tertiary care settings. Neither of the systematic reviews pooled data statistically. Tenderness of the spine, the presence of neurological deficits and the presence of back bruising were the most common index tests investigated. All clinical tests showed very small to small LR+ when considered independently, LR+ ranging from 0.69 CI 95% (0.22, 2.17) to 3.32 CI 95% (0.22, 50.86). Only one study [105] conducted in a tertiary care setting found that the presence of back bruising was superior to other tests, reporting large-magnitude LR+ (31.09 CI 95% [18.25, 52.96]).

Malignancy

One systematic review [45], including three primary studies [90,93,117] conducted in primary care, investigated the diagnostic accuracy of physical examination findings for the diagnosis of spinal malignancy (see Table S23). The presence of neurological symptoms, fever (>100 °F), muscle spasm and spinal tenderness were the index tests evaluated. When used in isolation, all these clinical tests showed poor sensitivity values, ranging from 0 to 0.15 CI 95% (0.02, 0.45), while the specificity ranged from 0.60 CI 95% (0.58, 0.62) to 0.97 CI 95% (0.95, 0.96). No further diagnostic accuracy data were provided.

Spinal Infection

Two systematic reviews [41,42], including two primary studies [91,109], evaluated the diagnostic accuracy of physical examination findings for the diagnosis of spinal infection in patients with LBP presenting to the ED (see Table S24). Based on one retrospective study of medical files [91], having fever, if present alone, did not significantly alter the post-test probability of spinal infection, reflected by a LR+ of 1.71 CI 95% (1.04, 2.81). Based on one primary study conducted in emergency department (ED), a moderate-magnitude LR+ (9.0 CI 95% [0.89, 1.01]) was found for a systolic blood pressure < 90 mmHg in diagnosing epidural abscess.

Any Serious Spinal Pathologies

Two systematic reviews [41,42], including two primary studies [96,110] conducted in the ED, provided data on the diagnostic accuracy of physical examination findings for the diagnosis of serious spinal pathology (see Table S25). Anal tone loss or faecal incontinence, spine tenderness, fever, saddle anesthesia, bladder/suprapubic fullness and sensory deficits were investigated. Of the six index tests, only anal tone loss (LR+ 6.3 CI 95% [1.9, 20.8]), saddle anesthesia (LR+ 7.0 CI 95% [1.4, 36.0]–11 CI 95% [3.1, 39.6]) and bladder/suprapubic fullness (LR+ 40.2 CI 95% [1.6, 979.1]) significantly altered the post-test probability of serious spinal pathology. No clear association between each index test and the concomitant pathology was established.

#### 3.6.4. Diagnostic Support Tools

Cauda Equina Syndrome

One systematic review [42], including two primary studies [91,112], investigated the diagnostic accuracy of index test combinations in diagnosing CES (Table S26). The combination of [1] a recent loss of bladder control and [2] a recent loss of bowel control, with (LR+ 3.46) or without saddle anesthesia (LR+ 3 CI 95% [1.01, 8.92]) improved post-test probability of CES in patients presenting with LBP.

Spinal Fracture

One systematic review [42], including four primary studies [89,90,91,92], provided data on the diagnostic accuracy of clinical support tools for the diagnostic of spinal fracture (Table S27). According to Premkumar et al. [91], diagnostic accuracy was increased by, respectively, 13.1% and 20.5%, when combining [1] a history of recent trauma to [2] age > 50 years (LR+ 2.54 CI 95% [2.05, 3.16]) or age > 70 years (LR+ 4.35 CI 95% [2.92, 6.48]). Enthoven et al. [89] identified a diagnostic prediction model combining multiple index tests (i.e., osteoporosis, age ≥ 75 years, trauma, back pain intensity score ≥ 7/10 and thoracic pain). Small-to-moderate LR+ were attributed to the presence of two or more (3.6 CI 95% [2.8, 4.8]) and three or more positive features (5.8 CI 95% [3.2, 10.8]). Henschke et al. [90] also investigated the combination of four clinical features (i.e., history of trauma, advanced age, prolonged use of corticosteroids and female gender) in detecting osteoporotic spinal fracture in patients presenting with LBP in a primary care setting. Post-test probability of spinal fracture increased up to 52% in the presence of three or more positive signs (LR+ 906.11 CI 95% [50.37, 16,299.11]). Finally, Roman et al. [92] evaluated the combination of [1] age > 52 years; [2] absence of leg pain; [3] BMI ≤ 22; [4] does not exercise regularly; and [5] female gender. Four positive tests yielded a moderate increase in the likelihood of spinal fracture (LR+ 9.6 CI 95% [3.7, 14.9]).

Malignancy

Two systematic reviews [42,45], including two primary studies [91,93], provided data on combinations of index tests for the diagnosis of spinal malignancy (Table S28). Premkumar et al. [91] evaluated the combination of [1] unexplained weight loss; and [2] history of spinal malignancy, which increased the probability of a spinal malignancy up to 14.3% (LR+ 10.25 CI 95% [3.6, 29.21]). Deyo et al. [93] also discussed the diagnostic accuracy of a combination of index tests (i.e., age greater than 50 years, history of malignancy, unexplained weight loss and failure to improve with conservative therapy), reporting a sensitivity of 100% when all 4 index tests were positive. No further data on this combination of tests were provided.

Spinal Infection

Two systematic reviews [41,42], including three primary studies [91,109,118], investigated the diagnostic accuracy of clinical support tools for the diagnosis of spinal infection (Table S29). Premkumar et al. [91] found that combination of [1] fever; [2] chills or sweating, associated with [3] a recent infection, increased the post-test probability of spinal infection up to 13.8% (LR+ 13.15 CI 95% [6.66, 25.97]). Two primary studies [109,118] evaluated the diagnostic accuracy of the combination of [1] fever ≥38 °C; [2] spinal pain; and [3] neurological deficits in detecting spinal infection in patients with LBP presenting to the ED. Also known as the “classic triad”, this combination yielded to a moderate increase in the likelihood of spinal infection (LR+ 5.7 CI 95% [1.4, 23.2]–10.0).

Inflammatory Back Pain (IBP)

One systematic review [47], including four primary studies [119,120,121,122], investigated the diagnostic accuracy of clinical support tools for the diagnosis of IBP (Table S30). Three studies [120,121,122] provided evidence on the diagnostic value of the Berlin criteria (i.e., morning stiffness, improvement in back pain with exercise but not with rest, awakening because of pain in the second half on the night, and alternating buttock pain) for the diagnosis of IBP. Positive likelihood ratios for the cutoff point of two or more predictors being present ranged from 2.8 CI 95% (1.2, 6.3) to 3.8 CI 95% (2.8, 5.0), indicating this prediction rule may have a small influence on the likelihood of IBP. Two studies [119,122] investigated the diagnosis performance of a five-item prediction rule for identifying patients with IBP. Using a cutoff point of four or more predictors being present, this prediction rule was identified to be more sensitive and less specific than the Berlin criteria, but presented similar positive likelihood ratios, ranging from 2.9 to 3.4. None of these studies were conducted in primary care settings and all used expert rheumatologists’ opinions as a reference standard.

## 4. Discussion

Most clinical practice guidelines recommend diagnostic triage to classify patients into one of three categories of LBP (i.e., non-specific LBP, radicular syndrome and specific LBP). Diagnostic recommendations also emphasize that triaging of patients should be achieved by performing a focused history-taking that enables the identification of patients with specific conditions as the underlying cause of LBP, and a physical examination that assesses the presence of neurological signs. Aside from these recommendations, little guidance is provided as to which clinical features are of appropriate diagnostic value and therefore should be questioned or assessed when triaging LBP patients. As the burden of disabling LBP continues to grow, actions are needed to develop evidence-based and standardized evaluation procedures that will promote diagnostic accuracy, and therefore the appropriate use of health resources. This can only be achieved with a clear understanding of the relevant clinical features that should be used in clinical practice.

To this end, our scoping review aimed to summarize evidence investigating the diagnostic value of patient evaluation components applicable in primary care settings for the diagnosis of LBP and to clarify to what extent patient history and physical examination findings can inform clinical decisions.

Most of the eligible systematic reviews provided data obtained from various healthcare settings (i.e., primary, secondary, and tertiary), but did not specifically examine the impact of the clinical context on diagnostic test performance. Diagnostic accuracy data were predominantly derived from secondary and tertiary care settings, with only 16 individual studies having been conducted exclusively in primary care. Of these, 15 studies focused on the diagnostic value of potential indicators of underlying spinal pathology (i.e., CES, spinal fracture, malignancy, infection and spondyloarthritis). Although it is crucial to ensure that these conditions are not overlooked, their prevalence is quite low in primary care. Interestingly, 62.5% of these studies were published before 2010, most of which present heterogenous findings, highlighting the need for further research that investigates the diagnostic value of each component of patient evaluation. Due to clinical heterogeneity, only six (22.2%) systematic reviews performed a meta-analysis. Therefore, the diagnostic values of demographic, patient history and physical examination findings in identifying patients with LSS, radicular syndrome, non-specific LBP, and specific LBP were descriptively reported in the original reviews.

The following sections outline the patient evaluation components that have demonstrated appropriate diagnostic value and that could be potentially useful for the diagnostic triage of patients with LBP.

### 4.1. Demographics

Twenty-two primary studies investigated the diagnostic value of demographic variables, including age, gender, BMI, job type, smoking status, living situation, and education level for the diagnosis of patients with LBP. Overall, only age and BMI appeared clinically informative as stand-alone findings. Older age at different cutoffs (i.e., >65 years, >70 years, and >75 years) consistently increased the likelihood of LSS and spinal fracture. One secondary care study identified “BMI < 22” as a valuable clinical finding for the diagnosis of osteoporotic spinal fracture. When combined with other clinical findings, female gender and “age > 50 years” were also identified as clinically informative for the diagnosis of spinal fracture and spinal malignancy, respectively. As such, age, BMI and gender should be considered as potentially valuable demographic variables for the diagnostic triage of patients with LBP.

### 4.2. Patient History

Twenty-two primary studies provided data on the diagnostic accuracy of patient history findings for the diagnosis of LBP. Most studies (17 of 22) investigated the diagnostic value of clinical findings used to diagnose LSS or specific LBP. Bilateral lower-extremity symptoms and “leg pain worse than back pain” significantly increased the likelihood of LSS when used in isolation. Pain relief upon sitting, improvement of symptoms with lumbar flexion, and pain exacerbation while standing up or walking showed inconsistent diagnostic accuracy as stand-alone findings but appeared to increase the likelihood of LSS when used in combination. For the diagnosis of specific LBP, bladder/bowel/saddle dysfunction, a previous history of trauma, prolonged corticosteroid use, a recent infection, and immunosuppression were identified as clinically informative when used in isolation. Dermatomal distribution of pain was the only index test identified as clinically informative in at least two primary studies for the diagnosis of radicular syndrome. All other index tests investigated yielded imprecise or poor diagnostic accuracy data. Overall, dominant site of pain (back or leg), pain distribution (dermatomal or non-dermatomal; unilateral or bilateral), aggravating or relieving factors and indicators of underlying spinal pathology should all be questioned when triaging patients with LBP.

### 4.3. Physical Examination

One hundred primary studies investigated the diagnostic accuracy of physical examination findings for the diagnosis of LBP. Inconsistent evidence supports the use of neurological examination components (i.e., sensory deficits, motor deficits, impaired reflexes) as stand-alone findings for the diagnosis of LSS and radicular syndrome. There is promising, yet imprecise evidence supporting the use of the treadmill walking test (i.e., neurological changes induced by level walking) and of lumbar ranges of motion (i.e., symptoms induced by having the patient bend backward) in identifying patients with LSS. Neurodynamic tests (i.e., Slump test and SLR) exhibited variable diagnostic value in diagnosing patients with radicular syndrome in secondary and tertiary care settings and were identified as not clinically informative in primary care populations. Based on the current literature, there is insufficient evidence to recommend the use of other physical examination findings in isolation when triaging patients with LBP.

### 4.4. Clinical Support Tools

After reviewing several studies that investigated the diagnostic performance of clinical support tools for the diagnosis of LBP, our analysis revealed that some clinical elements, deemed uninformative when used alone, should still be considered when evaluating patients with LBP. For the diagnosis of non-specific LBP, the presence of three or more positive provocation tests (i.e., distraction, compression, thigh thrust, sacral thrust and Gaenslen’s test) appears potentially useful to diagnose LBP originating from the SIJ. For the diagnosis of spinal malignancy, a previous history of malignancy, unexplained weight loss, and failure to improve with conservative care should be questioned, as well as the presence of fever, spinal pain, and neurological deficits for the diagnosis of spinal fracture. Finally, the presence of morning stiffness, improvement in back pain with exercise but not rest, awakening because of pain in the second half of the night, and alternating buttock pain were identified as clinically informative when used in combination for the diagnosis of IBP.

### 4.5. Factors Affecting Interpretation

Interpretation of our review findings may be influenced by several factors. First, most primary studies were conducted in secondary or tertiary care settings, predominantly including surgical populations. These studies generally selected patients based on a specific set of positive clinical and imaging findings, which may not be representative of patients presenting in primary settings. This could result in an overestimation of diagnostic performance. Our review also highlighted that despite a substantial increase in available evidence on patient evaluation components used for the diagnosis of LBP in the past 20 years, several index tests have been investigated by a small number of studies, still lack adequate evidence, and demonstrate imprecise diagnostic accuracy values. Moreover, many clinical features endorsed by clinical practice guidelines were not investigated in primary care settings. For instance, guidelines from seven different countries recommend using “disturbance of urinary and bowel sphincters”, and “saddle anesthesia” for the diagnosis of CES [123]. However, only one primary care study investigated these characteristics and found that they resulted in only a small increase in the post-test probability of CES [112].

Additionally, it is important to note that most studies poorly described index test procedures and did not provide cutoff values for positivity. Further, most studies provided diagnostic accuracy values for index tests used in isolation, rather than in combination with other clinical findings. This limits the applicability of the study results in clinical practice, as patient evaluation components are usually considered in combination to estimate the likelihood of a condition. To address these limitations, Finucane et al. [14], proposed an international framework that is intended to assist healthcare providers in identifying patients with underlying spinal conditions who may require further investigation or referral to a medical specialist. The authors emphasized the importance of not just considering the presence or absence of red flags when deciding whether to refer a patient or not, but also the clinical setting in which a patient presents, the quality of evidence supporting the use of each clinical finding, and the potential impact on patient outcomes.

### 4.6. Limitations and Future Research Directions

Our scoping review has some limitations. Despite conducting robust systematic searches in multiple relevant databases, studies not published in English or French (authors’ native languages) were excluded, which may have resulted in relevant studies being missed. However, it has been reported that excluding non-English publications from evidence-syntheses does not lead to bias, as it would have a minimal effect on overall conclusions [124,125]. It is important to note that the existing literature is considerably limited in providing recommendations for the assessment of LBP patients with more complex clinical profiles, for example, those presenting characteristics that may fall into more than one category of LBP. Therefore, our review findings may not be fully applicable to this subgroup of patients. The results of this scoping review must also be interpreted with caution, as a comprehensive evaluation of systematic reviews’ quality was not conducted.

## 5. Conclusions

This review provides a summary of the current evidence investigating the diagnostic value of patient evaluation components applicable in primary care settings for the diagnosis of LBP. Overall, most demographic, patient history and physical examination findings used for the diagnosis of LBP lack diagnostic accuracy when considered in isolation. Based on the available evidence, demographics (i.e., age, gender, and BMI), primary site of pain, pain distribution, aggravating and relieving factors, and indicators of underlying spinal pathology should all be questioned when triaging patients with LBP. A standardized physical examination should at least include a thorough neurological examination, combining the assessment of sensory, motor, and reflex deficits. Although several diagnostic studies have been published in recent years, our review highlights the need for evidence-based and standardized evaluation procedures, especially for primary care settings where evidence is still scarce. This is of high importance to promote the appropriate use of healthcare resources and to enable LBP patients to get a timely access to appropriate healthcare providers.

## Figures and Tables

**Table 1:** Likelihood ratios interpretation.

Likelihood Ratios	Interpretation
+LR > 10 or −LR < 0.1	Large
+LR 5–10 or −LR 0.1–0.2	Moderate
+LR 2–5 or −LR 0.2–0.5	Small
+LR 1–2 or −LR 0.5–1	Very small

**Table 2:** Systematic reviews characteristics.

LUMBAR SPINAL STENOSIS									
First Author	Year of Publication	End of Search	Country	Settings of Data Collection	Study Design(s)	Number of Primary Studies	Number of Participants	Population	Meta-Analysis
Cook	2020 [22]	Nov 2018	USA	Mixed	Prospective (7); Multicenter cross-sectional (1); Retrospective cohort (1)	*n* = 9	N = 36,228	Adult (>18 yrs) patients with LBP of any duration with a suspicion of LSS	No
De Schepper	2013 [23]	March 2011	NLD	Mixed	Prospective	*n* = 15	N = 2909	Adult patients with LSS	No
RADICULAR SYNDROME (*Lumbar radiculopathy, LDH*)									
Mistry	2020 [24]	July 2019	UK	Mixed	Cross-sectional observational study (11)	*n* = 11	N = 3908	Adult participants with LBLP	No
Tawa	2017 [25]	July 2016	Kenya	Mixed	Cohort study (11); case control (1)	*n* = 12	N = 1026	Subjects with clinical signs and symptoms consistent with lumbo-sacral radiculopathy.	No
Al Nezari	2013 [26]	March 2011	NZ	Mixed	Prospective cohort (12); Case-control (2)	*n* = 14	N = 7200	Patients with LBP of any duration with suspicion of radiculopathy caused by a potential LDH	Yes
Scaia	2012 [27]	Dec 2011	USA	Mixed	Case control, case-based case control, and cohort studies	*n* = 7	N = 4311	Patients with suspected LDH, lumbar radiculopathy or sciatica	No
Van der Windt	2010 [28]	April 2008	UK	Mixed	Case-control study (3); prospective cohort (11); retrospective cohort (5)	*n* = 19	Cohort: median N = 126, range 71-2504 Case control: 38–100 cases	Patients with low-back pain with pain radiating into the leg, who were suspected of having radiculopathy due to LDH	Yes
Devillé	2000 [29]	1997	NLD	Mixed	Unclear	*n* = 15	NA	Unclear	Yes
NON-SPECIFIC LOW BACK PAIN									
Han	2023 [30]	Jan 2023	Australia	Mixed (secondary and tertiary care)	NA	*n* = 62	Ranged from 15 to 736	Patients with LBP without serious pathology	Yes
Nolet	2021 [31]	July 2019	Canada	NA	NA	*n* = 7	N = 777	Adult patients with LBP with or without radiculopathy of any duration	No
Stolz	2020 [32]	Sept 2019	Germany	Mixed	NA	*n* = 13 (3 validity studies)	N = 235	Included at least one group of adult participants that had suffered from LBP	No
Maas	2017 [33]	June 2016	NLD	Mixed	Cross-sectional (10); case-control (1); Retrospective cohort (1)	*n* = 12	N = 1504	Adult patients, of either gender, suffering from CLBP	No
Grodahl	2016 [34]	Nov 2015	Norway	Mixed	Prospective (3); Retrospective (2); Cross-sectional (1); Non-experimental (1); Case series (1)	*n* = 8	N = 654	Population with LBP with/without radiculopathy presenting with suspected spondylolysis and/or spondylolisthesis	No
Ferrari	2015 [35]	Dec 2013	Italy	Mixed	NA	*n* = 6	N = 333	Adult population with sub-acute or chronic LBP	No
Sivayogam	2011 [36]	Feb 2011	Singapore	NA	NA	*n* = 6	N = 409	Adult patients with non-specific, non-pregnancy related LBP and/or buttock pain, with or without lower- extremity symptoms	No
Alqarni	2011 [37]	March 2010	NZ	Mixed	Prospective (3); Cross-sectional (1)	*n* = 4	N = 351	Patient with CLBP or with mixed lumbar pathology	No
Hancock	2007 [38]	Feb 2006	Australia	NA	NA	*n* = 41	NA	Patients with low back pain and no known or suspected serious pathology	No
Simpson	2006 [39]	Dec 2005	UK	NA	NA	*n* = 11	NA	Adult patients with LBP	No
SPECIFIC LOW BACK PAIN									
Tabrah	2022 [40]	Oct 2020	UK	Mixed	Retrospective cohort (5); Prospective cohort (1)	*n* = 6	N = 679	People presenting with acute CES	Yes
Galliker	2020 [41]	Jan 2019	Switzerland	Mixed	Prospective cohort (3); Retrospective cohort (15); Cross-sectional (3); Mixed (1)	*n* = 22 (10 on DA of RF)	N = 41,320	Adult patients presenting with LBP of any duration to an ED	No
Maselli	2020 [42]	June 2020	Italy	Mixed	Retrospective (19); Prospective (1); Cross-sectional (3); Observational (6); Cohort (1)	*n* = 40 (21 focused on LBP patients)	N = 49,422	Patients consulting healthcare professionals for LBP	No
Dionne	2019 [43]	Jan 2018	Canada	Mixed	Retrospective cohort (6); Prospective cohort (1)	*n* = 7	N = 869	Adults who presented with suspected CES from an insidious onset or herniated disc prolapse	Yes
Williams	2013 [44]	March 2012	Australia	Mixed (primary and secondary)	Prospective cohort (6); Retrospective cohort (2)	*n* = 8	N = 7378	Patients presenting with LBP or for lumbar spine examination	No
Henschke	2013 [45]	April 2012	Germany	Mixed	Prospective cohort (6); Retrospective cohort (2)	*n* = 8	N = 8905	Patients with LBP or requiring examination of the lumbar spine	No
Henschke	2008 [46]	Feb 2007	Australia	Mixed	Prospective (8); Retrospective (4)	*n* = 12	N = 7147	Patients with back pain presenting to the ED	No
MIXED									
Haskins	2015 [47]	July 2013	Australia	Mixed	Prospective (*n* = 9); Retrospective (*n* = 2); NA (*n* = 4)	*n* = 15	NA	Mixed	No
Shultz	2015 [48]	Nov 2013	USA	Mixed	Prospective (*n* = 9); Retrospective (*n* = 1); Cross-sectional (*n* = 1)	*n* = 11	N = 2899	Patients with low back pain (LBP) and related lower-extremity pain condition	No

CES: Cauda equina syndrome; CLBP: Chronic low back pain; DA: Diagnostic accuracy; ED: Emergency department; LBLP: Low back-related leg pain; LBP: Low back pain; LDH: Lumbar disc herniation; LSS: Lumbar spinal stenosis; NA: Not available; RF: Red Flag.

## Data Availability

All extracted data are available upon request from the corresponding author.

## Supplementary Materials

The following supporting information can be downloaded at: https://www.mdpi.com/article/10.3390/jcm12103581/s1, Table S1: Diagnostic accuracy of demographics for lumbar spinal stenosis (LSS); Table S2: Diagnostic accuracy of patient history findings for LSS; Table S3: Diagnostic accuracy of physical examination findings for LSS; Table S4: Diagnostic accuracy of clinical diagnostic support tool for LSS; Table S5: Diagnostic accuracy of demographics for lumbar radiculopathy; Table S6: Diagnostic accuracy of patient history findings for lumbar radiculopathy; Table S7: Diagnostic accuracy of physical examination findings for lumbar radiculopathy; Table S8: Diagnostic accuracy of demographics for non-specific LBP; Table S9: Diagnostic accuracy of patient history findings for non-specific LBP; Table S10: Diagnostic accuracy of physical examination findings for non-specific LBP; Table S11: Diagnostic accuracy of clinical diagnostic support tools for the diagnosis of non-specific LBP; Table S12: Diagnostic accuracy of demographics for the diagnostic of cauda equina syndrome; Table S13: Diagnostic accuracy of demographics for the diagnostic of spinal fracture; Table S14: Diagnostic accuracy of demographics for the diagnosis of spinal malignancy; Table S15: Diagnostic accuracy of demographics for the diagnosis any serious spinal pathology; Table S16: Diagnostic accuracy of patient history findings for the diagnosis of CES; Table S17: Diagnostic accuracy of patient history findings for the diagnosis of spinal fracture; Table S18: Diagnostic accuracy of patient history findings for the diagnosis of spinal malignancy; Table S19: Diagnostic accuracy of patient history findings for the diagnosis of spinal infection; Table S20: Diagnostic accuracy of patient history findings for the diagnosis of serious spinal pathology; Table S21: Diagnostic accuracy of physical examination findings for the diagnosis of CES; Table S22: Diagnosis accuracy of physical examination findings for the diagnosis of spinal fracture; Table S23: Diagnostic accuracy of physical examination findings for the diagnosis of spinal malignancy; Table S24: Diagnostic accuracy of physical examination findings for the diagnosis of spinal infection; Table S25: Diagnosis accuracy of physical examination findings for the diagnosis of serious spinal pathology; Table S26: Diagnosis accuracy of diagnostic support tools for the diagnosis of CES; Table S27: Diagnosis accuracy of diagnostic support tools for the diagnosis of spinal fracture; Table S28: Diagnostic accuracy of diagnostic support tools for the diagnosis of spinal malignancy; Table S29: Diagnostic accuracy of diagnostic support tools for the diagnosis of spinal infection; Table S30: Diagnostic accuracy of diagnostic support tools for the diagnosis of inflammatory back pain. File S1: Medline Search Strategy.
