# Supplementary material for: Diagnosis Value of Patient Evaluation Components Applicable in Primary Care Settings for the Diagnosis of Low Back Pain: A Scoping Review of Systematic Reviews
Source: J Clin Med. 2023 May 21;12(10):3581. doi: 10.3390/jcm12103581 (PMC10218868; doi:10.3390/jcm12103581)
Supplement: Supplementary file 1 [file jcm-12-03581-s001.zip › jcm-2404742-supplementary.pdf]

Table S1: Diagnostic accuracy of demographics for lumbar spinal stenosis (LSS)

**Systematic review of diagnostic accuracy of patient history, clinical findings, and physical tests in the diagnosis of lumbar spinal stenosis (Cook, 2020)**

PRIMARY STUDIES

| First author | Year of publication | Setting(s) of data collection | Number of participants | Population | Reference standard | Index test | Diagnostic accuracy data |
|---|---|---|---|---|---|---|---|
| Cook | 2011 | Tertiary | N= 1448 | Consecutive patients with suspicion of a condition associated with origin at the lumbar spine | Expert opinion based on clinical findings and imaging (MRI | Age > 48 years | Se 0.88 (0.85, 0.91); SP 0.49 (0.46, 0.52); +LR 1.70 (1.60, 1.80); -LR 0.25 (0.21, 0.32); +PTP % (95% CI): 53.46 (52, 56); +PTP absolute differences %: 13.33 **(medium)**; -PTP % (95% CI): 14.45 (11, 17); -PTP absolute differences %: 25.88 **(large)** |

**Diagnosis of Lumbar Spinal Stenosis An Updated Systematic Review of the Accuracy of Diagnostic Tests (De Shepper, 2013)**

PRIMARY STUDIES

| First author | Year of publication | Setting(s) of data collection | Number of participants | Population | Reference standard | Index test | Diagnostic accuracy data |
|---|---|---|---|---|---|---|---|
| Konno | 2007 | Primary and secondary | N=468 | Consecutive patients showing primary symptoms of pain or numbness in the lower extremities, including the buttocks, thighs, and lower legs | Consensus diagnostic impression of expert physicians, confirmation by x-rays and MRI | Age < 60 years | Se 0.15 (0.11,0.20); Sp 0.62 (0.56,0.68); +LR 0.41; -LR 1.4 |
| | | | | | | Age > 70 years | Se 0.64 (0.58, 0.71); Sp 0.68 (0.62, 0.74); +LR 2.0; -LR 0.52 |
| Katz | 1995 | Tertiary | N=75 | Patients with low back pain with or without radiation to the lower extremities | Expert opinion (>80% confidence in diagnosis for cases and <20% for non-cases) | Age > 65 years | Se 0.77 (0.64,0.90); Sp 0.69 (0.53,0.85); +LR 2.5; -LR 0.33 |

Table S2: Diagnostic accuracy of patient history findings for LSS

| Systematic review of diagnostic accuracy of patient history, clinical findings, and physical tests in the diagnosis of lumbar spinal stenosis (Cook, 2020) | | | | | | | |
|---|---|---|---|---|---|---|---|
| PRIMARY STUDIES | | | | | | | |
| First author | Year of publication | Setting(s) of data collection | Number of participants | Population | Reference standard | Index test | Diagnostic accuracy data |
| Cook | 2011 | Tertiary | N= 1448 | Consecutive patients with suspicion of a condition associated with origin at the lumbar spine | Expert opinion based on clinical findings and imaging (MRI) | Bilateral symptoms | Se 0.03 (0.02, 0.05); Sp 0.98 (0.97, 0.99); +LR 2.30 (1.10, 4.80); -LR 0.98 (0.97, 0.99); +PTP %(95% CI): 60.85 (35, 66); +PTP absolute differences %: 20.52 (medium); -PTP %(95% CI): 39.84 (40, 41); -PTP absolute differences %: 0.49 (small) |
| | | | | | | Leg or back pain worse | Se 0.17 (0.14, 0.20); Sp 0.92 (0.90, 0.94); +LR 2.10 (1.50, 2.80); -LR 0.91 (0.87, 0.94); +PTP %(95% CI): 58.66 (50, 64); +PTP absolute differences %: 18.33 (medium); −PTP % (95% CI): 38.02 (37, 38); −PPT absolute differences %: 2.31 (small) |
| | | | | | | Moderate back pain | "Se 0.95 (0.93, 0.97); Sp 0.02 (0.01, 0.03); +LR 0.97 (0.96, 0.99); -LR 2.10 (1.20, 3.90); PTP %(95% CI): 39.59 (39, 40); +PTP absolute differences %: 0.74 (small); −PTP % (95% CI): 63 (49, 75); −PPT absolute differences %: 22.67 (medium)" |
| | | | | | | Moderate buttock pain | Se 0.81 (0.78, 0.84); Sp 0.33 (0.30, 0.36); +LR 1.20 (1.10, 1.30); -LR 0.60 (0.46–0.72); PTP %(95% CI): 44.78 (44, 47); +PTP absolute differences %: 4.45 (small); -PTP % (95% CI): 28.85 (24, 32); −PPT absolute differences %: 11.48 (small) |
| | | | | | | Moderate leg pain | Se 0.90 (0.87, 0.92); Sp 0.24 (0.21, 0.27); +LR 1.20 (1.10, 1.20); -LR 0.43 (0.32, 0.58); PTP %(95% CI): 44.78 (43, 46); +PTP absolute differences %: 4.45 (small); −PTP % (95% CI):22.51 (18, 27); −PPT absolute differences %: 18.72 (medium) |
| | | | | | | Pain constancy | Se 0.23 (0.20, 0.27); Sp 0.78 (0.75, 0.81); +LR 1.10 (0.86, 1.30); -LR 0.98 (0.92, 1.04); PTP %(95% CI):42.64 (37, 46) ; +PTP absolute differences %: 2.31 (small);−PTP % (95% CI): 39.84 (39, 41); −PPT absolute differences %: 0.49 (small) |

| | | | | | | | |
|---|---|---|---|---|---|---|---|
| | | | | | | Pain with walking/ standing | Se 0.67 (0.64, 0.69); Sp 0.44 (0.42, 0.46); +LR 1.2; -LR 0.75 |
| | | | | | | Sitting relieves pain | Se 0.26 (0.24, 0.29); Sp 0.86 (0.84,0.88); +LR 1.9, -LR 0.86 |
| Sugiokia | 2008 | Primary and secondary | N=469 | Patients showing primary symptoms of pain or numbness in the lower limbs, including the buttocks, thighs and lower legs | Physician-diagnosed LSS | Change position frequently to try and get lower extremities comfortable | Se 0.73 (0.66, 0.79); Sp 0.39 (0.32, 0.47); +LR 1.19 (1.03, 1.38); -LR 0.70 (0.52, 0.94); +PTP % 57.50 (54, 61); +PTP absolute differences %: 4.29 (small); −PTP % 44.32 (37, 51); −PPT absolute differences %: 8.89 (small) |
| | | | | | | Improvement of symptoms when bending backward | Se 0.16 (0.11, 0.21); Sp 0.78 (0.71, 0.84); +LR 0.70 (0.45, 1.08); -LR 1.08 (0.98, 1.20); +PTP % 45 (34, 56); +PTP absolute differences %: 8.21 (small); −PTP % 55 (53, 58); −PPT absolute differences %: 1.79 (small) |
| | | | | | | Have to hold onto something to get out of an easy chair due to lower extremities | Se 0.43 (0.36, 0.50); Sp 0.68 (0.61, 0.75); +LR 1.35 (1.03, 1.76); -LR 0.83 (0.71, 0.98); +PTP % 60.55 (54, 67); +PTP absolute differences %: 7.34 (small); −PTP % 48.55 (45, 53); −PPT absolute differences %: 4.66 (small) |
| | | | | | | Numbness of lower legs while walking | Se 0.75 (0.68, 0.81); Sp 0.37 (0.29, 0.44); +LR 1.18 (1.03, 1.36); -LR 0.68 (0.50, 0.93); +PTP % 58.29 (54, 61); +PTP absolute differences %: 5.08 (small); −PTP % 43.60 (36, 51); −PPT absolute differences %: 9.61 (small) |
| | | | | | | Only stand for short periods of time due to lower extremities | Se 0.71 (0.65, 0.78); Sp 0.43 (0.35, 0.51); +LR 1.25 (1.07, 1.46); -LR 0.67 (0.50, 0.88); +PTP % 58.70 (55, 62); +PTP absolute differences %: 5.49 (small); −PTP % 43.24 (37, 50); −PPT absolute differences %: 10 (small) |
| | | | | | | Only walk for short distances due to lower extremities | Se 0.76 (0.69, 0.82); Sp 0.37 (0.29, 0.44); +LR 1.21 (1.05, 1.38); -LR 0.65 (0.47, 0.89); +PTP % 57.91 (54, 61); +PTP absolute differences %: 4.70 (small); −PTP % 57 (49, 65); −PPT absolute differences %: 3.79 (small) |
| | | | | | | Use a handrail to get upstairs due to lower extremities | Se 0.66 (0.59, 0.73); Sp 0.66 (0.57, 0.75); +LR 1.27 (1.06, 1.50); -LR 0.71 (0.55, 0.91); +PTP % 59.08 (55, 63); +PTP absolute differences %: 5.87 (small); |

| | | | | | | | |
|---|---|---|---|---|---|---|---|
| | | | | | | | −PTP % 44.67 (38, 51); −PPT absolute differences %: 8.54 (small) |
| | | | | | | Walking is easier when bending forward | Se 0.55 (0.48, 0.62); Sp 0.61 (0.53, 0.68); +LR 1.42 (1.14, 1.78); -LR 0.73 (0.60, 0.89); +PTP % 61.75 (56, 67); +PTP absolute differences %: 8.54 (small); −PTP % 45.36 (41, 50); −PPT absolute differences %: 7.85 (small) |
| | | | | | | Walk more slowly than usual due to lower extremities | Se 0.86 (0.81, 0.91); Sp 0.27 (0.20, 0.34); +LR 1.17 (1.06, 1.30); -LR 0.52 (0.34, 0.79); +PTP % 57.09 (55, 60); +PTP absolute differences %: 3.88 (small); −PTP % 37.16 (28, 47); −PPT absolute differences %: 16.05 (medium) |
| | | | | | | Change position frequently to try and get lower extremities comfortable | Se 0.73 (0.66, 0.79); Sp 0.39 (0.32, 0.47); +LR 1.19 (1.03, 1.38); -LR 0.70 (0.52, 0.94); +PTP % 57.50 (54, 61); +PTP absolute differences %: 4.29 (small); −PTP % 44.32 (37, 51); −PPT absolute differences %: 8.89 (small) |
| | | | | | | Improvement of symptoms when bending backward | Se 0.16 (0.11, 0.21); Sp 0.78 (0.71, 0.84); +LR 0.70 (0.45, 1.08); -LR 1.08 (0.98, 1.20); +PTP % 45 (34, 56); +PTP absolute differences %: 8.21 (small); −PTP % 55 (53, 58); −PPT absolute differences %: 1.79 (small) |
| | | | | | | Exacerbated while standing up | Se 0.92 (0.88, 0.96); Sp 0.20 (0.14, 0.27); +LR 1.2; -LR 0.39 |
| | | | | | | Wake up to urinate at night | Se 0.86 (0.82, 0.91); Sp 0.27 (0.20, 0.33); +LR 1.2; -LR 0.51 |
| Konno | 2007 | Primary and secondary | N=468 | Consecutive patients showing primary symptoms of pain or numbness in the lower extremities, including the buttocks, thighs and lower legs | MRI | Bilateral plantar numbness | Se 0.27 (0.21, 0.33); Sp 0.87 (0.83, 0.91); +LR 2.14 (1.45, 3.18); -LR 0.83 (0.76, 0.92); +PTP % 65.88 (57, 74); +PTP absolute differences %: 18.44 (medium); −PTP % 42.82 (41, 45); −PPT absolute differences %: 4.62 (small) |
| | | | | | | Leg pain or numbness | Se 0.58 (0.51, 0.64); Sp 0.55 (0.49, 0.62); +LR 1.30 (1.09, 1.56); -LR 0.76 (0.63, 0.92); +PTP % 53.98 (49, 58); +PTP absolute differences %: 6.54 (small); −PTP % 40.68 (36, 46); −PPT absolute differences %: 6.76 (small) |
| | | | | | | Low back pain | Se 0.66 (0.60, 0.72); Sp 0.41 (0.35, 0.48); +LR 1.13 (0.98, 1.30); -LR 0.82 (0.64, 1.03); +PTP % 50 (47, 54); +PTP absolute differences %: 2.56 (small); |

| | | | | | | | |
|---|---|---|---|---|---|---|---|
| | | | | | | | −PTP % 42.53 (37, 48); −PPT absolute differences %: 4.91 (small) |
| | | | | | | Numbness of perineal region | Se 0.05 (0.02, 0.08); Sp 0.99 (0.96, 1.00); +LR 3.69 (1.03, 13.25); -LR 0.97 (0.94, 1.00); +PTP % 76.90 (48, 93); +PTP absolute differences %: 29.46 (large); −PTP % 46.68 (46, 47); −PPT absolute differences %: 0.76 (small) |
| Fritz | 1997 | Tertiary | N=45 | Patients with low back and lower extremity pain and self-reported limitations in walking tolerance | MRI and CT scan | Walk better holding onto a shopping cart | Se 0.63 (0.38, 0.84); Sp 0.67 (0.35, 0.90); +LR 1.89 (0.79, 4.53); -LR 0.55 (0.27, 1.13); +PTP %(95% CI):75 (56, 88) ; +PTP absolute differences %: 14 (medium) ; −PTP % (95% CI): 47 (30, 64) ; −PPT absolute differences %: 14 (medium) |
| Roach | 1997 | Tertiary | N=106 | Patients with recurrent or chronic low back pain | Patient's complaints, results of physical examination, diagnostic radiologic tests, laboratory work, consultations, and any other diagnostic tests | Pseudoclaudication | Se 0.63 (0.41, 0.81); Sp 0.71 (0.60, 0.80); +LR 2.20 (1.38, 3.50); -LR 0.52 (0.31, 0.89); +PTP % 39.16 (29, 50); +PTP absolute differences (%): 16.52 (medium); −PTP % 13.20 (8, 21); −PPT absolute differences (%): 9.44 (small) |
| Katz | 1995 | Tertiary | N=75 | Patients with low back pain with or without radiation to the lower extremities | The expert attending physician's confidence in the diagnosis of LSS (>80% confidence in diagnosis for cases and <20% for non-cases) | No pain with flexion | Se 0.79 (0.64, 0.90); Sp 0.44 (0.26, 0.62); +LR 1.41 (1.00, 1.98); -LR 0.48 (0.24, 0.96); +PTP % 65.45 (57, 73); +PTP absolute differences %: 25.12 (large); −PTP % 39.20 (24, 56); −PPT absolute differences %: 1.13 (small) |
| | | | | | | No pain when seated | Se 0.47 (0.31, 0.62); Sp 0.94 (0.79, 0.99); +LR 7.21 (1.82, 28.61); -LR 0.57 (0.43, 0.77); +PTP % 90.64 (71, 97); +PTP absolute differences %: 33.31 (large); −PTP % 43.36 (37, 51); −PPT absolute differences %: 13.97 (medium) |
| | | | | | | Numbness | Se 0.63 (0.47, 0.77); Sp 0.59 (0.41, 0.76); +LR 1.50 (0.93, 2.40); -LR 0.64 (0.39, 1.05); +PTP % 66.83 (56, 77); +PTP absolute differences %: 9.50 (small); −PTP % 46.23 (34, 58); −PPT absolute differences %: 11 (medium) |

| | | | | | | | |
|---|---|---|---|---|---|---|---|
| | | | | | | Pain below buttocks | Se 0.88 (0.75, 0.96); Sp 0.34 (0.19, 0.53); +LR 1.35 (1.02, 1.77); -LR 0.34 (0.13, 0.88); +PTP % 64.46 (58, 70); +PTP absolute differences %: 7.13 (small); −PTP % 31.35 (16, 55); −PPT absolute differences %: 25.98 (large) |
| | | | | | | Pain below knees | Se 0.56 (0.40, 0.71); Sp 0.63 (0.44, 0.79); +LR 1.49 (0.88, 2.50); -LR 0.71 (0.46, 1.09); +PTP % 66.68 (55, 77); +PTP absolute differences %: 9.35 (small); −PTP % 52 (41, 62); −PPT absolute differences %: 5.33 (small) |
| | | | | | | Severe lower extremity pain | Se 0.65 (0.49, 0.79); Sp 0.66 (0.47, 0.81); +LR 2.02 (1.16, 3.52); -LR 0.51 (0.32, 0.83); +PTP % 73.07 (61, 82); +PTP absolute differences %: 15.74 (medium); −PTP % 40.66 (31, 53); −PPT absolute differences %: 16.67 (medium) |

**Diagnostic accuracy of self-report and subjective history in the diagnosis of low back pain with non-specific lower extremity symptoms: A systematic review (Shultz, 2015)**

PRIMARY STUDIES

| First author | Year of publication | Setting(s) of data collection | Number of participants | Population | Reference standard | Index test | Diagnostic accuracy data |
|---|---|---|---|---|---|---|---|
| Beattie | 2000 | NA | N=428 | Patients with low back pain or lower extremity radiculopathy | MRI | Lower extremity pain for LSS | DOR: 2.09 (1.03,4.26) |

**Diagnosis of Lumbar Spinal Stenosis An Updated Systematic Review of the Accuracy of Diagnostic Tests (De Schepper, 2013)**

PRIMARY STUDIES

| First author | Year of publication | Setting(s) of data collection | Number of participants | Population | Reference standard | Index test | Diagnostic accuracy data |
|---|---|---|---|---|---|---|---|
| Cook | 2011 | Tertiary | N=1448 | Consecutive patients with suspicion of a condition associated with origin at the lumbar spine | Expert opinion based on clinical findings and imaging (MRI) | Pain with walking/ standing | Se 0.67 (0.64, 0.69); Sp 0.44 (0.42, 0.46); +LR 1.2; -LR 0.75 |
| | | | | | | Sitting relieves pain | Se 0.26 (0.24, 0.29); Sp 0.86 (0.84,0.88); +LR 1.9, -LR 0.86 |

| | | | | | | | |
|---|---|---|---|---|---|---|---|
| Sugiokia | 2008 | Primary and secondary | N=469 | Consecutive patients showing primary symptoms of pain or numbness in the lower extremities, including the buttocks, thighs and lower legs | Consensus diagnostic impression of expert physicians, confirmation by x-rays and MRI | Exacerbated while standing up | Se 0.92 (0.88, 0.96); Sp 0.20 (0.14, 0.27); +LR 1.2; -LR 0.39 |
| | | | | | | Treatment for symptoms needs to be repeated every year | Se 0.40 (0.33, 0.47); Sp 0.81 (0.75, 0.86); +LR 2.1; -LR 0.74 |
| | | | | | | Wake up to urinate at night | Se 0.86 (0.82, 0.91); Sp 0.27 (0.20, 0.33); +LR 1.2; -LR 0.51 |
| Konno | 2007 | Primary and secondary | N=468 | Patients showing primary symptoms of pain or numbness in the lower extremities, including the buttocks, thighs and lower legs | Consensus diagnostic impression of expert physicians, confirmation by x-rays and MRI | Burning sensation around the buttocks and/or intermittent priapism associated with walking | Se 0.06 (0.03, 0.09); Sp 0.99 (0.98, 1.00); +LR 7.2; -LR 0.95 |
| | | | | | | Intermittent claudication | Se 0.82 (0.77, 0.87); Sp 0.78 (0.73, 0.83); +LR 3.7; -LR 0.23 |
| | | | | | | Exacerbation when standing up | Se 0.68 (0.62, 0.74); Sp 0.70 (0.65, 0.76); +LR 2.3; -LR 0.45 |
| | | | | | | Improvement when bending forward | Se 0.52 (0.45, 0.58); Sp 0.92 (0.88, 0.95); +LR 6.4; -LR 0.52 |
| | | | | | | Urinary disturbance | Se 0.14 (0.09, 0.19); Sp 0.98 (0.96, 1.00); +LR 6.9; -LR 0.88 |
| Fritz | 1997 | Tertiary | N=45 | Patients with low back and lower extremity pain and self-reported limitations in walking tolerance | MRI or CT scan | Pain in legs relieved by sitting | Se 0.81 (0.61, 0.93); Sp 0.16 (0.03, 0.40); +LR 1.0; -LR 1.2 |
| | | | | | | Sitting best posture with regard to symptoms | Se 0.89 (0.70, 0.98); Sp 0.39 (0.17, 0.64); +LR 1.5, -LR 0.28 |
| | | | | | | Walk/stand worst posture with regard to symptoms | Se 0.89 (0.70, 0.98); Sp 0.33 (0.13, 0.59); +LR 1.3, -LR 0.33 |
| Roach | 1997 | Tertiary | N=106 | Patients with recurrent or | Patient's complaints, results of physical examination, | Radiating leg pain | Se 0.94; Sp 0.21; +LR 1.2; -LR 0.29 |

| | | | | | | | |
|---|---|---|---|---|---|---|---|
| | | | | chronic low back pain | diagnostic radiologic tests, laboratory work, consultations, and any other diagnostic tests | | |
| Katz | 1995 | Tertiary | N=75 | Patients with low back pain with or without radiation to the lower extremities | Expert opinion (>80% confidence in diagnosis for cases and <20% for non-cases) | Symptoms improve when seated | Se 0.52 (0.37,0.67); Sp 0.83 (0.70,0.96); +LR 3.1; -LR 0.58 |
| | | | | | | Worse when walking | Se 0.71 (0.57,0.85); Sp 0.30 (0.14,0.46); +LR 1.0; -LR 0.97 |
| Ljunggren | 1991 | Secondary | N=179 | Consecutive patients with lumbago-sciatica and no previous back surgery. | Diagnosis by physical and neurological examination, imaging and surgery | Pain relief with assuming a suitable body position | Se 0.61 (0.50,0.72); Sp 0.55 (0.45, 0.65); +LR 1.4; -LR 0.71 |
| | | | | | | Bilateral buttock or leg pain | Se 0.51 (0.40,0.62); Sp 0.92 (0.87,0.97); +LR 6.3; -LR 0.54 |
| | | | | | | Lumbosacral pain | Se 0.75 (0.65,0.84); Sp 0.27 (0.18,0.36); +LR 1.0; -LR 0.94 |
| | | | | | | Gluteal pain | Se 0.84 (0.75,0.92); Sp 0.05 (0.01,0.09); +LR 0.88; -LR 3.3 |
| | | | | | | Thigh pain | Se 0.95 (0.90,1.00); Sp 0.14 (0.07,0.20); +LR 1.1, -LR 0.37 |
| | | | | | | Calf pain | Se 0.91 (0.85,0.97); Sp 0.06 (0.01, 0.11); +LR 0.97; -LR 1.5 |
| | | | | | | Foot pain | 0.57 (0.46–0.68) 0.27 (0.18–0.36) 0.78 1.6 |

Table S3: Diagnostic accuracy of physical examination findings for LSS

| Systematic review of diagnostic accuracy of patient history, clinical findings, and physical tests in the diagnosis of lumbar spinal stenosis (Cook, 2020) | | | | | | | |
|---|---|---|---|---|---|---|---|
| PRIMARY STUDIES | | | | | | | |
| First author | Year of publication | Setting(s) of data collection | Number of participants (N) | Population | Reference standard | Index test | Diagnostic accuracy data |
| Dobbs | 2016 | NA | N= 30 | Subjects aged 50 or over with subjective report of unilateral or bilateral pain or paraesthesia radiating below the gluteal fold | MRI | Modified extension test | Se 0.92 (0.74, 0.99); Sp 0.40 (0.05, 0.85); +LR 1.53 (0.74, 1.36); -LR 0.20 (0.00, 1.36); +PTP %(95% CI): 88 (79, 94); +PTP absolute differences (%): 5 (small); −PTP % (95% CI): 50 (17, 85); −PPT absolute differences (%): 33 (large) |
| Konno | 2007 | Primary and secondary | N=468 | Consecutive patients showing primary symptoms of pain or numbness in the lower extremities, including the buttocks, thighs, and lower legs | Consensus diagnostic impression of expert physicians, confirmation by x-rays and MRI | Straight leg raising test positive | Se 0.17 (0.12, 0.22); Sp 0.67 (0.61, 0.73); +LR 0.51 (0.36, 0.72); -LR 1.24 (1.12, 1.38); +PTP % 31.52 (25, 39); +PTP absolute differences (%): 15.92 (medium); −PTP % 52.81 (50, 55); −PPT absolute differences (%): 5.37 (small) |
| | | | | | | Symptoms induced by having patients bend forward | Se 0.18 (0.13, 0.23); Sp 0.63 (0.56, 0.69); +LR 0.48 (0.34, 0.66); -LR 1.31 (1.17, 1.47); +PTP % 30.22 (23, 37); +PTP absolute differences (%): 17.22 (medium); −PTP % 54.17 (51, 57); −PPT absolute differences (%): 6.73 (small) |
| | | | | | | Symptoms induced by having patients bend backward | Se 0.70 (0.63, 0.76); Sp 0.54 (0.48, 0.61); +LR 6.34 (4.09, 9.84); -LR 0.53 (0.46, 0.61); +PTP % 58 (54, 62); +PTP absolute differences (%): 10.56 (small); −PTP % 32.35 (28, 39); −PPT absolute differences (%): 15.09 (medium) |
| Fritz | 1997 | Tertiary | N=45 | Patients with low back and | MRI or CT scan | Prolonged recovery after level walking | Se 0.82 (0.60, 0.95); Sp 0.68 (0.43, 0.87); +LR 2.59 (1.30, 5.17); -LR 0.26 (0.10, 0.68); +PTP %(95% |

| | | | | | | | |
|---|---|---|---|---|---|---|---|
| | | | | lower extremity pain and self-reported limitations in walking tolerance | | | CI): 75 (60, 86) ; +PTP absolute differences (%): 21 (medium); −PTP % (95% CI): 24 (10, 44); −PPT absolute differences (%): 30 (large) |
| | | | | | | Two-stage treadmill test (on a level; earlier onset of symptoms with level walking) | Se 0.68 (0.46, 0.85); Sp 0.83 (0.59, 0.96); +LR 4.0 (1.40, 11.86); -LR 0.38 (0.21, 0.71); +PTP %(95% CI): 84.55 (66, 94) ; +PTP absolute differences (%): 26.55 (large); −PTP % (95% CI):34.21 (23, 50); −PPT absolute differences (%): 23.79 (medium) |
| | | | | | | Two-stage treadmill test (on an inclined treadmill; longer total walking time during inclined walking) | Se 0.50 (0.25, 0.75); Sp 0.92 (0.64, 1.00); +LR 6.50 (0.93, 45.50); -LR 0.54 (0.32, 0.91); +PTP %(95% CI): 89.89 (53, 98) ; +PTP absolute differences (%): 34.89 (large) ; −PTP % (95% CI): 40 (28, 53); −PPT absolute differences (%): 15 (medium) |
| Katz | 1995 | Tertiary | N=75 | Patients with low back pain with or without radiation to the lower extremities | Expert opinion (>80% confidence in diagnosis for cases and <20% for non-cases) | Thigh pain with 30 s of lumbar extension | Se 0.51 (0.35, 0.67); Sp 0.69 (0.50, 0.84); +LR 1.64 (0.91, 2.96); -LR 0.71 (0.48, 1.04); +PTP % 68.78 (55, 80); +PTP absolute differences %: 11.45 (medium); −PTP % 48.82 (39, 58); −PPT absolute differences %: 8.51 (small) |
| | | | | | | Abnormal Romberg | Se 0.40 (0.25, 0.56); Sp 0.91 (0.75, 0.98); +LR 4.06 (1.29, 12.76); -LR 0.68 (0.53, 0.89); +PTP % 84.50 (64, 95); +PTP absolute differences (%): 27.17 (large); −PTP % 47.74 (41, 54); −PPT absolute differences (%): 8.87 (small) |
| | | | | | | Absent Achilles reflex | Se 0.47 (0.31, 0.62); Sp 0.78 (0.60, 0.91); +LR 2.07 (0.99, 4.31); -LR 0.70 (0.50, 0.98); +PTP % 73.55 (58, 85); +PTP absolute differences (%): 16.22 (medium); −PTP % 48.46 (40, 56); −PPT absolute differences (%): 8.87 (small) |
| | | | | | | Pinprick deficit (sensibility) | Se 0.47 (0.31, 0.62); Sp 0.81 (0.64, 0.93); +LR 2.54 (1.16, 5.58); -LR 0.64 (0.46, 0.90); +PTP % 77.33 (61, 88); +PTP absolute differences (%): 20 (medium); −PTP % 46.23 (39, 55); −PPT absolute differences (%): 11.10 (medium) |
| | | | | | | Poor balance | Se 0.70 (0.54, 0.83); Sp 0.53 (0.35, 0.71); +LR 1.49 (0.98, 2.26); -LR 0.57 (0.33, 0.99); +PTP % 66.68 (57, 75); +PTP absolute differences (%): 9.35 |

| | | | | | | | |
|---|---|---|---|---|---|---|---|
| | | | | | | | (small); −PTP % 43.36 (30, 57); −PPT absolute differences (%): 13.97 (medium) |
| | | | | | | Vibration deficit | Se 0.53 (0.38, 0.69); Sp 0.81 (0.64, 0.93); +LR 2.79 (1.28, 6.07); -LR 0.59 (0.41, 0.84); +PTP % 78.94 (63, 89); +PTP absolute differences (%): 21.61 (medium); −PTP % 44.21 (36, 53); −PPT absolute differences (%): 13.12 (medium) |
| | | | | | | Weakness | Se 0.47 (0.31, 0.62); Sp 0.78 (0.60, 0.91); +LR 2.18 (1.05, 4.51); -LR 0.67 (0.48, 0.94); +PTP % 74.54 (58, 86); +PTP absolute differences (%): 17.21 (medium); −PTP % 47.37 (40, 56); −PPT absolute differences (%): 9.96 (small) |
| Jensen | 1989 | NA | N=23 | Indication for myelography; symptoms: neurogenic claudication, unilateral in 14 cases, bilateral in 9 cases | Myelographical examination | Any change of neurological status after walk test (treadmill) | Se 1.00 (0.63, 1.00); Sp 0.33 (0.12, 0.62); +LR 1.50 (1.05, 2.15); -LR 0; +PTP % 44.44 (34, 53); +PTP absolute differences (%): 9.44 (small); −PTP % 0 (0, 58); −PPT absolute differences (%): 35 (large) |
| | | | | | | Bilateral paresis after walk test (treadmill) | Se 0.38 (0.09, 0.76); Sp 0.87 (0.60, 0.98); +LR 2.81 (0.59, 13.52); -LR 0.72 (0.41, 1.28); +PTP % 59.97 (24, 88); +PTP absolute differences (%): 25 (medium); −PTP % 27.74 (18, 41); −PPT absolute differences (%): 7.26 (small) |
| | | | | | | Bilateral reflex changes after walk test (treadmill) | Se 0.50 (0.16, 0.84); Sp 0.80 (0.52, 0.96); +LR 2.50 (0.73, 8.52); -LR 0.62 (0.30, 1.31); +PTP % 57.14 (28, 82); +PTP absolute differences (%): 22.14 (large); −PTP % 24.84 (14, 41); −PPT absolute differences (%): 10.16 (small) |
| | | | | | | Functional test (neurological status change as a result of a treadmill test) | Se 1.00 (0.63, 1.00); Sp 0.33 (0.12, 0.62); +LR 1.50 (1.05, 2.15); -LR 0; +PTP % 44.44 (34, 53); +PTP absolute differences (%): 9.44 (small); −PTP % 0 (0, 58); −PPT absolute differences (%):35 (large) |
| | | | | | | Symptom-march after a walk test (treadmill) | Se 0.63 (0.24, 0.91); Sp 0.80 (0.52, 0.96); +LR 3.12 (0.99, 9.83); -LR 0.46 (0.19, 1.19); +PTP % 62.45 (35, 84); +PTP absolute differences (%): 27.45 (large); −PTP % 19.69 (9, 39); −PPT absolute differences (%): 15.31 (medium) |
| **Diagnosis of Lumbar Spinal Stenosis An Updated Systematic Review of the Accuracy of Diagnostic Tests (De Shepper, 2013)** | | | | | | | |
| PRIMARY STUDIES | | | | | | | |

| First author | Year of publication | Setting(s) of data collection | Number of participants (N) | Population | Reference standard | Index test | Diagnostic accuracy data |
|---|---|---|---|---|---|---|---|
| Cook | 2011 | Tertiary | N=1448 | Consecutive patients with suspicion of a condition associated with origin at the lumbar spine | Expert opinion based on clinical findings and imaging (MRI) | Gait abnormality | Se 0.29 (0.27, 0.32); Sp 0.81 (0.79, 0.83); +LR 1.6; -LR 0.87 |
| Katz | 1995 | Tertiary | N=75 | Patients with low back pain with or without radiation to the lower extremities | Expert opinion (>80% confidence in diagnosis for cases and <20% for non-cases) | Wide-based gait | Se 0.43 (0.28, 0.58); Sp 0.97 (0.91,1.00); +LR 14; -LR 0.59 |

Table S4: Diagnostic accuracy of clinical diagnostic support tool for LSS

| **Diagnostic Clinical Prediction Rules for Specific Subtypes of Low Back Pain: A Systematic Review (Haskins, 2015)** | | | | | | | |
|---|---|---|---|---|---|---|---|
| PRIMARY STUDIES | | | | | | | |
| First author | Year of publication | Setting(s) of data collection | Number of participants (N) | Population | Reference standard | Index test | Diagnostic accuracy data |
| Sugiokia | 2008 | Primary and secondary | N=469 | Patients showing primary symptoms of pain or numbness in the lower limbs, including the buttocks, thighs and lower legs | Physician-diagnosed LSS | Clinical prediction rule (score ≥5 : older age, duration of symptoms > 6m, improvement of symptoms when bending backward, occurrence of symptoms when standing up, improvement of symptoms while resting, urinary incontinence | Se 0.75; Sp 0.51; LRs not reported but calculated to be LR+ 1.5 (1.1, 2.1); LR- 0.50 (0.29, 0.88) |
| **Diagnosis of Lumbar Spinal Stenosis An Updated Systematic Review of the Accuracy of Diagnostic Tests (De Shepper, 2013)** | | | | | | | |
| PRIMARY STUDIES | | | | | | | |
| First author | Year of publication | Setting(s) of data collection | Number of participants (N) | Population | Reference standard | Index test | Diagnostic accuracy data |
| Cook | 2011 | Tertiary | N=1448 | Consecutive patients with suspicion of a condition associated with origin at the lumbar spine | Expert opinion based on clinical findings and imaging (MRI) | 1 of 5 positive findings (bilateral symptoms, leg pain more than back pain, pain during walking/standing, pain relief upon | Se 0.96 (0.94, 0.97); Sp 0.20 (0.19, 0.21); +LR 1.2, -LR 0.19 |

| | | | | | | | |
|---|---|---|---|---|---|---|---|
| | | | | | | sitting, age > 48 yr) | |
| | | | | | | 2 of 5 positive findings | Se 0.68 (0.65,0.71); Sp 0.62 (0.60, 0.64); +LR 1.8 ; -LR 0.51 |
| | | | | | | 3 of 5 positive findings | Se 0.29 (0.27,0.31); Sp 0.88 (0.87,0.90); +LR 2.5; -LR 0.80 |
| | | | | | | 4 of 5 positive findings | Se 0.06 (0.05, 0.07); Sp 0.98 (0.98, 0.99); +LR 4.6; -LR 0.95 |
| Kato | 2009 | Secondary | N=119 | Patients with symptoms in lower extremities | Consensus diagnostic impression of expert physicians, confirmation by x-rays, CT and MRI | Clinical diagnostic support tool (LSS ≥ 7) | Se 0.95 (0.89, 1.00); Sp 0.40 (0.28,0.52); +LR 1.6; -LR 0.13 |
| Konno | 2007 | Primary and secondary | N=468 | Consecutive patients showing primary symptoms of pain or numbness in the lower extremities, including the buttocks, thighs, and lower legs | Consensus diagnostic impression of expert physicians, confirmation by x-rays and MRI | Clinical diagnostic support tool (LSS ≥ 7) | Se 0.95 (0.89, 1.00); Sp 0.40 (0.28,0.52); +LR 1.6; -LR 0.13 |

Table S5: Diagnostic accuracy of demographics for lumbar radiculopathy

| **Diagnostic utility of patient history, clinical examination and screening tool data to identify neuropathic pain in low back related leg pain: a systematic review and a narrative synthesis (Mistry, 2020)** | | | | | | | |
|---|---|---|---|---|---|---|---|
| PRIMARY STUDIES | | | | | | | |
| First author | Year of publication | Setting(s) of data collection | Number of participants (N) | Population | Reference standard | Index test | Diagnostic accuracy data |
| Verwoerd | 2014 | Secondary | N=395 | Adults patients diagnosed by neurologist with incapacitating | MRI | Age > 40 | (Nerve root compression/herniated disc): Se 57%/58%; Sp 37%/36%; LR+ 0.9/0.9; LR- 1.16/1.17 |

| | | | | | | | |
|---|---|---|---|---|---|---|---|
| | | | | lumbosacral radicular syndrome | | Male sex | Se 66%/65%; Sp 47%/45%; LR+1.25/1.18; LR- 0.23/0.78 |
| | | | | | | BMI ≥ 30 | Se 13%/12%; Sp 78%/72%; LR+ 0.59/0.43; LR- 0.74/0.74 |
| | | | | | | Health-related absenteeism | Se 80%/81%; Sp 20%/24%; LR+ 1/0.7; LR- 1/0.79 |
| | | | | | | Having an intellectually heavy job | Se 69%/69%; Sp 42%/42%; LR+ 1.19/1.19; LR- 0.74/0.74 |
| | | | | | | Having a physically heavy job | Se 38%/39%; Sp 57%/60%; LR+ 0.95/0.95; LR- 1.05/1.03 |
| | | | | | | Smoking | Se 39%/39%; Sp 58%/59%; LR+ 0.95/0.95; LR- 1.05/1.03 |
| **Diagnostic accuracy of self-report and subjective history in the diagnosis of low back pain with non-specific lower extremity symptoms: A systematic review (Shultz, 2015)** | | | | | | | |
| PRIMARY STUDIES | | | | | | | |
| First author | Year of publication | Setting(s) of data collection | Number of participants (N) | Population | Reference standard | Index test | Diagnostic accuracy data |
| Coster | 2010 | Tertiary | N= 202 | Patients had suspicion of lumbosacral radiculopathy | Radiological nerve root compression | Age > 40 years | DOR: 1.20 (0.60, 2.20) |
| | | | | | | Gender male | DOR: 1.70 (0.90, 3.00) |
| Vroomen | 2002 | Primary and secondary | N=274 | Patients with pain radiating into the leg | MRI | Age 41-50 | DOR: 1.30 (0.70, 2.30) |
| | | | | | | Age 51-81 | DOR: 2.20 (1.20, 4.00) |
| | | | | | | Male Sex | DOR: 1.80 (1.10, 3.00) |
| | | | | | | Living Alone | DOR: 1.30 (1.70, 2.40) |
| | | | | | | High Education Level | DOR: 1.60 (0.90, 2.90) |
| | | | | | | Job Type: Cognitive | DOR: 1.10 (0.60, 2.00) |
| | | | | | | Job Type: Standing/walking/lifting | DOR: 0.50 (0.30, 0.95) |
| Vucetic | 1997 | NA | N=274 | Patients with pain radiating into the leg (suspicion of LDH) | MRI | Education | DOR: 3.22 (1.30, 7.80) |

Table S6: Diagnostic accuracy of patient history findings for lumbar radiculopathy

| **Diagnostic utility of patient history, clinical examination and screening tool data to identify neuropathic pain in low back related leg pain: a systematic review and a narrative synthesis (Mistry, 2020)** | | | | | | | |
|---|---|---|---|---|---|---|---|
| PRIMARY STUDIES | | | | | | | |
| First author | Year of publication | Setting(s) of data collection | Number of participants (N) | Population | Reference standard | Index test | Diagnostic accuracy data |
| Verwoerd | 2014 | | N=395 | Adults patients diagnosed by neurologist with incapacitating lumbosacral radicular syndrome | MRI | Duration of pain in leg ≥ 9 weeks | (Nerve root compression/herniated disc): Se 26%/25%; Sp 73%/71%; LR+ 0.96/0.86; LR- 1.01/1.06 |
| | | | | | | Pain worse in leg than back | Se 53%/52%; Sp 59%/59%; LR+ 1.29/1.27; LR- 0.8/0.81 |
| | | | | | | Having pain in the back > 12 weeks | Se 34%/36%; Sp 58%/61%; LR+ 0.81/0.92; LR- 1.14/1.25 |
| | | | | | | Sudden onset | Se 58%/60%; Sp 24%/24%; LR+ 0.76/0.79; LR- 1.75/0.53 |
| | | | | | | Paroxysmal pain | Se 38%/39%; Sp 59%/61%; LR+ 1.29/1.27; LR- 0.8/0.81 |
| | | | | | | Having had pain in the same leg previously | Se 12%/12%; Sp 90%/91%; LR+ 1.2/1.2; LR- 0.98/0.98 |
| | | | | | | Subjective sensory loss | Se 89%/90%; Sp 18%/25%; LR+ 1.09/1.2; LR- 0.61/0.13 |
| | | | | | | Subjective muscle weakness | Se 66%/67%; Sp 27%/31%; LR+ 0.9/0.97; LR- 1.63/1.06 |
| | | | | | | Positive family history | Se 42%/43%; Sp 62%/67%; LR+ 0.88/1.3; LR- 0.94/1.73 |
| | | | | | | Pain worse on coughing/sneezing/straining | Se 71%/71%; Sp 32%/31%; LR+ 1.04/1.03; LR- 0.91/0.94 |
| | | | | | | Worsening on sitting | Se 73%73%; Sp 22%/22%; LR+ 0.94/0,94; LR- 1.23/1.23 |

| **Diagnostic accuracy of self-report and subjective history in the diagnosis of low back pain with non-specific lower extremity symptoms: A systematic review (Shultz, 2015)** | | | | | | | |
|---|---|---|---|---|---|---|---|
| PRIMARY STUDIES | | | | | | | |
| First author | Year of publication | Setting(s) of data collection | Number of participants (N) | Population | Reference standard | Index test | Diagnostic accuracy data |
| Smart | 2012 | Secondary | N=102 | Patients >18 years of age with low back (±leg) pain referred for physiotherapy assessment and/or treatment | Experienced clinical judgment regarding the likely dominant mechanisms | History of nerve injury | DOR: 12.64 (3.59, 44.49) |
| | | | | | | Dermatomal distribution | DOR: 24.29 (6.33, 93.19) |
| Coster | 2010 | Tertiary | N= 202 | Patients had suspicion of lumbosacral radiculopathy | Radiological nerve root compression | Duration of symptoms 3-12w | DOR: 1.40 (0.80, 2.60) |
| | | | | | | Lumbago | DOR: 1.10 (0.50, 2.20) |
| | | | | | | HNP Surgery | DOR: 0.50 (0.20, 1.10) |
| | | | | | | Diabetes Mellitus | DOR: 0.80 (0.20, 3.00) |
| | | | | | | Physiotherapy | DOR: 1.10 (0.60, 1.90) |
| | | | | | | Use of pain killers | DOR: 1.70 (0.80, 3.60) |
| | | | | | | Dermatomal radiation | DOR: 4.10 (2.20, 7.80) |
| | | | | | | More pain on coughing, sneezing or straining | DOR: 3.20 (1.80, 5.70) |
| | | | | | | More pain on sitting | DOR: 1.90 (1.10, 3.40) |
| | | | | | | Less pain on standing | DOR: 0.90 (0.50, 1.50) |
| | | | | | | Less pain on lying down | DOR: 1.50 (0.80, 2.90) |
| | | | | | | Subjective muscle weakness | DOR: 2.20 (1.20, 4.00) |
| | | | | | | Subjective sensory loss | DOR: 2.10 (1.00, 4.20) |
| | | | | | | Disturbed Urinary Passage | DOR: 2.3 (0.20, 25.60) |
| Vroomen | 2002 | Primary and secondary | N=274 | Patients with pain radiating into the leg | MRI | Duration 15-30 days | DOR: 1.70 (0.90, 3.20) |
| | | | | | | Duration >30 days | DOR: 0.70 (0.40, 1.20) |
| | | | | | | Any Comorbidity | DOR: 1.30 (0.80, 2.10) |
| | | | | | | Smoking | DOR: 0.70 (0.40, 1.10) |
| | | | | | | Sports Activities | DOR: 1.10 (0.70, 2.2) |
| | | | | | | Exercised back/abd muscles | DOR: 0.80 (0.50, 1.30) |
| | | | | | | Family History Sciatica | DOR: 1.10 (0.70, 1.90) |

| | | | | | | | |
|---|---|---|---|---|---|---|---|
| Beattie | 2000 | | N=428 | Patients with low back pain or lower extremity radiculopathy | MRI | Leg pain for lumbar radiculopathy | DOR: 2.35 (1.36, 4.06) |
| | | | | | | Weakness for LDH | DOR: 1.86 (.99, 3.50) |
| Vucetic | 1997 | NA | N=274 | Patients with pain radiating into the leg (suspicion of LDH) | MRI | Duration of sciatica (week) | DOR: 0.76 (0.41, 1.11) |
| | | | | | | Progressive Sciatic Pain | DOR: 2.77 (1.20, 6.30) |
| | | | | | | Previous non-spinal surgery | DOR: 3.52 (1.60, 8.00) |

Table S7: Diagnostic accuracy of physical examination findings for lumbar radiculopathy

**Diagnostic utility of patient history, clinical examination and screening tool data to identify neuropathic pain in low back related leg pain: a systematic review and a narrative synthesis (Mistry, 2020)**

| PRIMARY STUDIES | | | | | | | |
|---|---|---|---|---|---|---|---|
| First author | Year of publication | Setting(s) of data collection | Number of participants (N) | Population | Reference standard | Index test | Diagnostic accuracy data |
| Lin | 2017 | Tertiary | N=60 | Patients with back pain with or without leg pain lasting > 3 months | MRI | Low strength | Se 82; Sp 92; LR+ 10.25; LR- 2.25 |
| | | | | | | High strength | Se 73; Sp 97; LR+ 24.33; LR- 0.97 |
| | | | | | | Pinprick | Se 55; Sp 100; LR+ 0; LR- 0.45 |
| | | | | | | Brush | Se 46; Sp 100; LR+ 0; LR- 0.54 |
| | | | | | | Blunt | Se 59; Sp 97; LR+ 10.67; LR- 0.42 |
| | | | | | | Vibration | Se 59; Sp 92; LR+ 7.38; LR- 0.45 |
| | | | | | | Warm | Se 68; Sp 97; LR+ 22.67; LR- 0.33 |
| | | | | | | Cold | Se 59; Sp 87; LR+ 4.54; LR- 0.47 |
| Urban | 2015 | Secondary | N=21 | Patients with LBP with or without leg pain, 25 years or > | Standard clinical assessment | Slump test | Se 91; Sp 70; LR+ 3.03; LR- 0.13 |
| Walsh | 2009 | NA | N=45 | Patients with unilateral LBLP | SLR + slump tests | Nerve palpation: 2 more of sciatic, tibial, common peroneal | Se 83; Sp 73; LR+ 3.07; LR- 0.23 |

| | | | | | | | |
|---|---|---|---|---|---|---|---|
| Poiraudeau | 2001 | Secondary | N=78 | Patients hospitalised for acute or chronic sciatica of mechanical origin | MRI, CT, saccoradiculography | Bell's test | Se 46; Sp 62; LR+ 1.21; LR- 0.87 |
| | | | | | | HE test | Se 44; Sp 67; LR+ 1.33; LR- 0.83 |
| | | | | | | Lasegue signs | Se 79; Sp 37; LR+ 1.25; LR- 0.57 |
| | | | | | | Crossed Lasegue sign | Se 29; Sp 83; LR+ 0.41; LR- 0.85 |

**Accuracy of clinical neurological examination in diagnosing lumbo-sacral radiculopathy: a systematic literature review (Tawa, 2017)**

PRIMARY STUDIES

| First author | Year of publication | Setting(s) of data collection | Number of participants (N) | Population | Reference standard | Index test | Diagnostic accuracy data |
|---|---|---|---|---|---|---|---|
| Iversen | 2013 | Outpatient multidisciplinary back clinics | N=116 | Patients with history & clinical presentation suggestive of chronic lumbar radiculopathy | MRI and CT | Sensory tests | Se 0.33 (0.06, 0.79); Sp 0.88 (0.81, 0.93); +LR 2.8; -LR 1.3 |
| | | | | | | Motor tests | Se 0.33 (0.06, 0.97); Sp 0.68 (0.59, 0.76); +LR 1.0; -LR 1.0 |
| | | | | | | Patellar reflex | Se 0.67 (0.21, 0.94); Sp 0.83 (0.75, 0.89); +LR 4.0; -LR 2.5 |
| | | | | | | Achilles reflex | Se 0.67 (0.21,0.94); Sp 0.60 (0.51, 0.69); +LR 1.7, -LR 1.8 |
| Suri | 2011 | Hospital spine center | N=54 | Patients with lower extremity radiating pain | MRI | Motor tests | **L3:** Se 0.50 (0.19, 0.81); Sp 0.77 (0.62, 0.89); +LR 2.2; -LR 1.5; **L4:** Se 0.54 (0.25, 0.81), Sp 0.80 (0.65, 0.91); +LR 2.7; -LR 1.7; **L5:** Se 0.61 (0.36, 0.83); Sp 0.86 (0.71, 0.95); +LR 4.4; -LR 2.2; **S1:** Se 0.29 (0.10, 0.56); Sp 0.97 (0.85, 1.00); +LR 1.0; -LR 1.4 |
| | | | | | | Achilles reflex | Se 0.33 (0.13, 0.59); Sp 0.91 (0.77, 0.98); +LR 3.7; -LR 1.4 |
| | | | | | | SLR | Se 0.29 (0.28, 0.32); Sp 0.57 (0.48, 0.58); +LR 0.7; -LR 0.8 |
| Trainor | 2011 | Orthopedic spinal clinic | N=16 | Patients with pain radiating into one or both legs distal to the groin or gluteal fold | MRI | Slump test | Se 1.00 (0.40, 1.00); 0.83 (0.52, 0.98); +LR 5.9; -LR 0.8 |

| | | | | | | | |
|---|---|---|---|---|---|---|---|
| | | | | Distribution of pain in dermatomal pattern | | | |
| Coster | 2010 | Tertiary | N=202 | Subjects referred by general practitioners with clinical suspicion of Lumbo-Sacral Radicular Syndrome (LSRS) | EMG | Patellar reflex | Se 0.18 (0.10, 0.18); Sp 0.66 (0.58, 0.71); +LR 0.5; -LR 0.8 |
| | | | | | | SLR | Se 0.44 (0.38, 0.52); Sp 1.00 (0.48, 1.00); +LR 0.4; -LR 1.8 |
| Suri | 2010 | Hospital spine center | N=51 | Patients presenting with lower extremity radiating pain and MRI- visible lumbar disk herniation. | MRI | Sensory (soft touch & pin prick) | **L2**: Se 0.08 (0.01, 0.27); Sp 0.96 (0.82, 1.00); +LR 2.0; -LR 1.0; **L3**: Se 0.17 (0.05, 0.37); Sp 0.96 (0.82, 1.00); +LR 4.3; -LR 1.2; **L4:** Se 0.17 (0.05, 0.37); Sp 1.00 (0.88, 1.00); +LR 0.2; -LR 1.2; **L5:** Se 0.13 (0.03, 0.34); Sp 0.82 (0.63, 0.94); +LR 0.7; -LR 0.9; **S1:** Se 0.08 (0.01, 0.27); Sp 0.79 (0.59, 0.92); +LR 0.4; -LR 0.9 |
| | | | | | | Motor (heel raise & sit-to-stand) | Se 0.39 (0.32, 0.52); Sp 0.83 (0.78, 0.87); +LR 2.3; -LR 1.4 |
| | | | | | | Patellar reflex | Se 0.32 (0.31, 0.53); Sp 0.90 (0.89, 0.95); +LR 3.2; -LR 1.3 |
| | | | | | | SLR | Se 0.64 (0.47, 0.82); Sp 0.48 (0.45, 0.50); +LR 1.2; -LR 1.3 |
| Bertilson | 2010 | Radiology clinic | N=61 | Patients referred for lumbar spine MRI | MRI | Sensory (soft touch & pain prick) | **L4:** Se 0.07 (0.01, 0.22); Sp 0.81 (0.63, 0.93); +LR 0.4; -LR 0.9; **L5:** Se 0.17 (0.06, 0.35); Sp 0.58 (0.39, 0.75); +LR 0.4; -LR 0.7; **S1**: Se 0.20 (0.08, 0.39); Sp 0.84 (0.66, 0.95); +LR 1.3; -LR 1.1 |
| | | | | | | Motor (hypotrophy) | L4: Se 0.13 (0.04, 0.31); Sp 0.87 (0.28, 3.76); +LR 1.0; -LR 1.0; **L5**: Se 0.27 (0.12, 0.46); Sp 0.68 (0.49, |

| | | | | | | | |
|---|---|---|---|---|---|---|---|
| | | | | | | | 0.83); +LR 0.8; -LR 0.9; **S1:** Se 0.17 (0.06, 0.35); Sp 0.81 (0.63, 0.93); +LR 0.9; -LR 1.0 |
| Majilesi | 2008 | Secondary | N=180 | Patients with complaints suggestive of lumbar disc herniation with low back, leg, or low back and leg pain | MRI | SLR | Se 0.52 (0.42, 0.58); Sp 0.89 (0.79, 0.95); +LR 4.7; -LR 1.9 |
| | | | | | | Slump test | Se 0.84 (0.74, 0.90); Sp 0.83 (0.73, 0.90); +LR 5.0; -LR 5.2 |
| Rabin | 2007 | Tertiary | N=38 | Low back pain or paraesthesia radiation below the knee | MRI | Seated SLRT and supine SLRT | Se 0.67 (0.53, 0.79); Sp 0.43 (0.38, 0.46); +LR 1.0; -LR 1.3 |
| Vroomen | 2002 | Primary | N=58 | Patients referred to the neurology department with a new episode of pain radiating into the leg below the gluteal fold | MRI | Sensory tests | Se 0.14 (0.09, 0.21); Sp 0.93 (0.87, 0.97); +LR 2.0; -LR 1.1 |
| | | | | | | Motor tests (paresis) | Se 0.27 (0.20, 0.35); Sp 0.93 (0.87, 0.97); +LR 3.9; -LR 1.3 |
| | | | | | | Achilles reflex | Se 0.14 (0.09, 0.21); Sp 0.93 (0.87, 0.97); +LR 2.0; -LR 1.1 |
| | | | | | | SLR | Se 0.64 (0.56, 0.71); Sp 0.57 (0.47, 0.66); +LR 1.5; -LR 1.6 |
| Haldeman | 1998 | Secondary | N=10 | Patients with complaints of low-back pain and leg pain, consistent with a diagnosis of sciatica | CT and electro-diagnostics | SLR | Se 0.37 (0.19, 0.58); Sp 0.78 (0.67, 0.87); +LR 1.7; -LR 1.2 |
| Albeck | 1996 | Secondary | N=80 | Patients with mono-radicular pain from L5 or S1 | Surgery | Sensory tests | Se 0.61 (0.47, 0.73); Sp 0.63 (0.38, 0.84); +LR 1.6; -LR 1.6 |
| | | | | | | Motor tests | Se 0.34 (0.23, 0.48); Sp 0.47 (0.24, 0.71); +LR 0.6; -LR 0.7 |

| | | | | | | | |
|---|---|---|---|---|---|---|---|
| | | | | | | Achilles reflex | Se 0.61 (0.47, 0.73); Sp 0.63 (0.38, 0.84); +LR 1.8; -LR 1.6 |
| | | | | | | SLR | Se 0.84 (0.72, 0.92); Sp 0.21 (0.06, 0.46); +LR 1.1; -LR 1.3 |
| **Neurological examination of the peripheral nervous system to diagnose lumbar spinal disc herniation with suspected radiculopathy: a systematic review and meta-analysis (Al Nezari, 2013)** | | | | | | | |
| **POOLED DATA** | | | | | | | |
| First author | Year of publication | Setting(s) of data collection | Number of participants (N) / Number of studies (n) | Population | Reference standard | Index test | Diagnostic accuracy data (pooled estimates) |
| Al Nezari | 2013 | Mixed | N=7200 | Patients with LBP with suspicion of radiculopathy caused by a potential LDH | Surgery | Sensory deficits | Se (0.40; CI 0.38, 0.43); Sp (0.59; CI 0.51, 0.67) |
| | | | | | Imaging findings | | Se (0.32; CI 0.28, 0.37); Sp (0.72; CI 0.67, 0.77) for diagnosis made at any lumbar segmental level and Se (0.35; CI 0.33, 0.38); Sp (0.64; CI 0.61, 0.66) for diagnosis made at a specific segmental level. |
| | | | | | Surgery | Motor deficits (paresis) | Se (0.22; CI 0.21, 0.23); Sp (0.79; CI 0.77, 0.80); +LRs (1.05; CI 0.87, 1.26) |
| | | | | | Imaging findings | | Se (0.40; CI 0.37, 0.42); Sp (0.62; CI 0.60, 0.64); +LRs (1.17; CI 0.99, 1.38) |
| | | | n=14 | | Surgery | Motor deficits (atrophy) | Se (0.31; CI 0.26, 0.36); Sp (0.76; CI 0.65, 0.85);+LRs (1.08; CI 0.34, 3.46) |
| | | | | | Surgery | Reflex deficits | Se (0.29; CI 0.28, 0.30); Sp (0.78; CI 0.76, 0.80); +LRs (1.26; CI 1.01, 1.58);-LR (0.87; CI 0.76, 0.98) |
| | | | | | Imaging findings | | Se (0.25; CI 0.22, 0.28); Sp (0.75; IC 0.73, 0.78); +LR (1.25; CI 0.71, 2.20); -LR (0.96; CI 0.82, 1.12) |

| | | | | | | | |
|---|---|---|---|---|---|---|---|
| **The pain provocation-based straight leg raise test for diagnosis of lumbar disc herniation, lumbar radiculopathy, and/or sciatica: a systematic review of clinical utility (Scaia, 2012)** | | | | | | | |
| **PRIMARY STUDIES** | | | | | | | |
| First author | Year of publication | Setting(s) of data collection | Number of participants | Population | Reference standard | Index test | Diagnostic accuracy data |
| Capra | 2011 | NA | N=1305 | Patients with suspected lumbar disc herniation, lumbar radiculopathy or sciatica | MRI | SLR | Se 36; Sp 74; +LR 1.39; -LR 0.87 |
| Poiraudeau | 2001 | Secondary | N=78 | | Electrodiagnosis | | Se 19; Sp 84; +LR 1.19; -LR 0.96 |
| Lauder | 2000 | NA | N=170 | | Myelography | | Se 96; Sp 10; +LR 1.07; -LR 0.4 |
| Kostelinatz | 1984 | Secondary | N=100 | | Myelography | | Se 76; Sp 45; +LR 1.38; -LR 0.53 |
| Spangfort | 1972 | Secondary | N=2377 | | MRI, CT or Myelography | | Se 52; Sp 89; +LR 4.73; -LR 0.54 |
| Knuttson | 1961 | Secondary | N=206 | | Myelography | | Se 77–83; Sp 36–39; +LR 1.20–1.36; -LR 0.44–0.63 |
| **Physical examination for lumbar radiculopathy due to disc herniation in patients with low-back pain (Van der Windt, 2010)** | | | | | | | |
| **POOLED DATA** | | | | | | | |
| First author | Year of publication | Setting(s) of data collection | Number of participants (N) / Number of studies (n) | Population | Reference standard | Index test | Diagnostic accuracy data (pooled estimates) |
| Van der Windt | 2010 | Primary and secondary care | Cohort studies: Median N = 126, range 71 to 2504; Case control studies: 38 to 100 cases n=19 | Patients with low-back pain with pain radiating into the leg (sciatica), who were suspected of having radiculopathy due to disc herniation | Imaging | SLR | Se 0.64 (0.56 to 0.71); Sp 0.57 (0.47 to 0.66) |
| | | | | | Surgery | | Se 0.92 (0.87, 0.95); Sp 0.28 (0.18, 0.40); +LR 1.3 (1.1, 1.4); -LR 0.30 (0.24, 0.39) |
| | | | | | Surgery | Crossed SLR | Se 0.28 (0.22, 0.35); Sp 0.90 (0.85, 0.94); +LR 2.1 (1.6, 2.8); -LR 0.86 (0.83, 0.89) |
| | | | | | Surgery | Scoliosis | Se range: 0.39 to 0.68; Sp range: 0.62 to 0.89 |

| | | | | | | | |
|---|---|---|---|---|---|---|---|
| | | | | | Surgery | Paresis or muscle weakness | Se range: 0.29 to 0.62; Sp range: 0.50 to 0.89 |
| | | | | | MRI | | Se 0.27 (0.20, 0.37); Sp 0.93 (0.88, 0.97) |
| | | | | | Surgery | Muscle wasting | Se range: range: 0.15 to 0.38; Sp range: 0.50 to 0.94 |
| | | | | | Surgery | Impaired reflex | Se range: 0.31 to 0.62; Sp range: 0.60 to 0.89 |
| | | | | | MRI | | Se 0.15 (0.09, 0.21); Sp 0.93 (0.88, 0.97) |
| | | | | | Surgery | Sensory deficits | Se range: 0.26 to 0.67; Sp range: 0.42 to 0.69 |
| | | | | | MRI | | Se 0.28 (0.21, 0.36); Sp 0.66 (0.56, 0.74) |
| | | | | | Surgery | Forward flexion | Se range: 0.85 and 0.90; Sp range: 0.16 and 0.29 |
| | | | n=16 | | MRI | | Se 0.45 (0.37, 0.53); Sp 0.74 (0.65, 0.81) |
| | | | | | MRI-CT | Extension test (sciatia was reproduced or worsened) | Se range: 0.13 to 0.90; Sp range: 0.17 to 0.94 (depending on cut-off point) |
| | | | | | MRT-CT | Slump test | Se range: 0.44 to 0.87; Sp range: 0.23 to 0.63 (depending on cut-off point) |
| | | | | | MRI-CT | Bell test | Se 0.49 (0.33, 0.65); Sp 0.63 (0.45, 0.79) |
| **The test of Lasègue: systematic review of the accuracy in diagnosing herniated discs. (Devillé, 2000)** | | | | | | | |
| POOLED DATA | | | | | | | |
| First author | Year of publication | Setting(s) of data collection | Number of participants (N) / Number of studies (n) | Population | Reference standard | Index test | Diagnostic accuracy data (pooled estimates) |

| | | | | | | | |
|---|---|---|---|---|---|---|---|
| Devillé | 2000 | Mixed | NA | Unclear | Surgery | SLR typical (+ SLR reproduces the sciatic pain between 30° and 60–75°. An atypical SLR was defined by three studies as pain produced in the back only) | Se 0.91 (0.82, 0.94); Sp 0.26 (0.16, 0.38); DOR 3.97 (3.22, 4.9) |
| | | | n=15 | | | Crossed SLR | Se 0.29 (0.24, 0.34); Sp 0.88 (0.86, 0.90); DOR 4.39 (0.74–25.9) |

Table S8: Diagnostic accuracy of demographics for non-specific LBP

**Systematic review of patient history and physical examination to diagnose chronic low back pain originating from the facet joints (Maas, 2017)**

PRIMARY STUDIES

| First author | Year of publication | Setting(s) of data collection | Number of participants (N) | Population | Reference standard | Index test | Diagnostic accuracy data |
|---|---|---|---|---|---|---|---|
| Laslett | 2006 | Secondary | N=120 | Adult patients, of either gender, suffering from CLBP | ZJ block 95% reduction standard | Age 50 or more | Se 0.62 (0.36, 0.82); Sp 0.77 (0.68,0.84) |
| Manchikanti | 2000 | Secondary (One private pain management practice, in a non- university setting) | N=200 | | Double diagnostic block | Age ≥ 65 | Se 0.21 (0.14, 0.31); Sp 0.85 (0.78, 0.91) |
| | | | | | | Old age | Se 0.25 (0.17, 0.35); Sp 0.84 (0.77, 0.90) |
| | | | | | | Positive work status | Se 0.34 (0.23, 0.47); Sp 0.62 (0.51, 0.72) |
| | | | | | | BMI (normal) | Se 0.40 (0.30, 0.51); Sp 0.69 (0.60, 0.77) |
| Manchikanti | 1999 | | N=120 | | | Age > 65 | Se 0.19 (0.10, 0.31); Sp 0.82 (0.71, 0.89) |
| | | | | | | Gender male | Se 0.39 (0.27, 0.52); Sp 0.56 (0.44, 0.67) |
| Revel | 1998 | Tertiary | N=80 | | 75% response on Lidocaine block | Age > 65 | Se 0.38 (0.18, 0.64); Sp 0.74 (0.62, 0.90) |

**Diagnostic utility of patient history and physical examination data to detect spondylolysis and spondylolisthesis in athletes with low back pain: A systematic review (Grodahl, 2016)**

PRIMARY STUDIES

| First author | Year of publication | Setting(s) of data collection | Number of participants | Population | Reference standard | Index test | Diagnostic accuracy data |
|---|---|---|---|---|---|---|---|
| Gregg | 2009 | Sports medicine clinic | N=71 | All patients with LBP referred for a SPECT scan to confirm suspected diagnosis of spondylolysis | SPECT scan | Age at bone scan (<20 yrs) | Se 80.77; Sp 43.64 |
| | | | | | | Gender male | Se 73.08; Sp 57.4 |

**Systematic review of tests to identify the disc, SIJ or facet joint as the source of low back pain (Hancock, 2007)**

| PRIMARY STUDIES | | | | | | | |
|---|---|---|---|---|---|---|---|
| First author | Year of publication | Setting(s) of data collection | Number of participants | Population | Reference standard | Index test | Diagnostic accuracy data |
| Manchikanti | 2000 | Secondary (One private pain management practice, in a non- university setting) | N=200 | Patients presenting to private pain management centre, with chronic LBP +/- lower extremity pain | Intra-articular local anaesthetic blocks for SIJ pain; and either intra-articular blocks or medial branch blocks for facet joint pain. | Age > 65 | Se 22 (14, 32); Sp 85 (77, 91); +LR 1.5 (0.8, 2.6); -LR 0.92 (0.80, 1.05) |
| Manchikanti | 1999 | | N=120 | | | Age > 65 | Se 19 (10, 32); Sp 66 (54,78); +LR 0.6 (0.3, 1.1); -LR 1.21 (0.98, 1.51) |
| Revel | 1998 | Tertiary | N=42 | Patients with LBP >3/12 referred for facet injection | | Age > 65 | Se 39 (15, 68); Sp 78 (60,91); +LR 1.8 (0.7,4.7); -LR 0.78 (0.49, 1.23) |

Table S9: Diagnostic accuracy of patient history findings for non-specific LBP

| **Low back pain of disc, sacroiliac joint, or facet joint origin: a diagnostic accuracy systematic review (Han, 2023)** | | | | | | | |
|---|---|---|---|---|---|---|---|
| PRIMARY STUDIES | | | | | | | |
| First author | Year of publication | Setting(s) of data collection | Number of participants (N) | Population | Reference standard | Index test | Diagnostic accuracy data |
| Depalma | 2011 | Tertiary care | N=160 | Patients with LBP that was either recalcitrant to spine-focused physical therapy, oral analgesics, or oral anti-inflammatory medication | Discography with a concordant pain provocation score of 6 out of 10 or greater and an adjacent pain-free control disc | Midline LBP (for pain originating from the disc) | Se 95.8 (88.1, 99.1); Sp 74.7 (65.0, 82.9); LR+ 3.79 (2.69, 5.34); LR- 0.06 (0.02, 0.17) |
| | | | | | | Para-midline LBP (for pain originating from the disc) | 67.3 (52.9 to 79.7) 9.3 (3.8 to 18.3) 3.50 (1.56 to 7.84) 0.74 (0.61 to 0.91) |
| | | | | | Greater than or equal to 80% pain relief with double blocks | Absence of midline LBP (for facet joint origin) | Se 15.4 (6.9, 28.1); Sp 28.0 (20.1, 37.0); LR+ 3.03 (2.22, 4.13); LR- 0.21 (0.11, 0.41) |
| | | | | | | Para-midline LBP (for facet joint origin) | Se 95 (83.1, 99.4); Sp 25.3 (16.6, 35.7); LR+ 1.27 (1.10, 1.47); LR- 0.20 (0.05, 0.80) |
| | | | | | Greater than or equal to 50% pain relief with SIJ double blocks | Absence of midline LBP (pain originating from the SIJ) | Se 12.9 (3.6, 29.8); Sp 36.0 (28.0, 44.5); LR+ 2.42 (1.87, 3.14); LR- 0.20 (0.08, 0.51) |
| | | | | | | Absence of midline LBP (pain originating from the SIJ) | Se 96 (79.6, 99.9); Sp 22.5 (14.9, 31.9); LR+ 1.24 (1.09, 1.41); LR- 0.18 (0.03, 1.25) |
| Lewinnek | 1986 | NA | N=21 | Patients with LBP | Greater than or equal to 80% pain relief with double blocks | Wakes from sleep (for pain originating from the facet joint) | Se 58.3 (27.7, 84.8); Sp 94.1 (56.7, 100.0); LR+ 9.92 (0.65, 152.24); LR- 0.44 (0.22, 0.88) |

| | | | | | | | |
|---|---|---|---|---|---|---|---|
| | | | | | | Acute onset | Se 95.2 (63.3, 100.0); Sp 50.0 (18.7, 81.3); LR+ 1.91 (1.01, 3.59); LR- 0.10 (0.01, 1.53) |
| | | | | | | Pain below knee | Se 88.9 (51.8, 99.7); Sp 63.6 (30.8, 89.1); LR+ 2.44 (1.08, 5.52); LR- 0.18 (0.03, 1.17) |
| | | | | | | Sitting increases pain | Se 63.6 (30.8, 89.1); Sp 83.3 (35.9, 99.6); LR+ 3.82 (0.60, 24.14); LR- 0.44 (0.19, 1.03) |
| | | | | | | Time-dependent positional distress | Se 78.6 (49.2, 95.3); Sp 66.7 (22.3, 95.7); LR+ 2.36 (0.74, 7.55); LR- 0.32 (0.10, 1.02) |
| | | | | | | NSAID helped | Se 50.0 (11.8, 88.2); Sp 91.7 (61.5, 99.8); LR+ 6.00 (0.78, 46.14); LR- 0.55 (0.24, 1.24) |
| **Systematic review of patient history and physical examination to diagnose chronic low back pain originating from the facet joints (Maas, 2017)** | | | | | | | |
| PRIMARY STUDIES | | | | | | | |
| First author | Year of publication | Setting(s) of data collection | Number of participants (N) | Population | Reference standard | Index test | Diagnostic accuracy data |
| Jung | 2007 | Secondary | N=259 | Patients had pain lasting more than 3 months and pain intensity greater than 5 on the NRS | Single or double diagnostic nerve blocks | Paravertebral area | Se 0.64 (0.55, 0.72); Sp 0.65 (0.56, 0.72) |
| | | | | | | Paravertebral area combined to leg pain in the posterior thigh and calf | Se 0.11 (0.07, 0.18); Sp 0.94 (0.88, 0.97) |
| | | | | | | Paravertebral area combined to leg pain in the posterior thigh only | Se 0.06 (0.03, 0.12); Sp 0.98 (0.95, 0.10) |
| | | | | | | Paravertebral area combined | Se 0.05 (0.03, 0.11); Sp 0.93 (0.87, 0.96) |

| | | | | | | | |
|---|---|---|---|---|---|---|---|
| | | | | | | with leg pain in the calf only | |
| | | | | | | Mixed patterns | Se 0.13 (0.08, 0.20); Sp 0.88 (0.81, 0.92) |
| Laslett | 2006 | Secondary | N=120 | Adult patients, of either gender, suffering from CLBP | ZJ block 95% reduction standard | Best activity is walking | Se 0.31 (0.13, 0.58); Sp 0.92 (0.85, 0.95) |
| | | | | | | Best activity is sitting | Sp 0.33 (0.14, 0.61); Sp 0.90 (0.82, 0.94) |
| | | | | | | Onset pain is paraspinal | Se 0.75 (0.47, 0.91); Sp 0.72 (0.63, 0.80) |
| | | | | | | MSPQ>13 | Se 0.46 (0.23, 0.71); Sp 0.70 (0.60, 0.77) |
| Manchikanti | 2000 | Secondary (One private pain management practice, in a non- university setting) | N=200 | | Double diagnostic block | Pain well relieved by supine | Se 0.94 (0.86, 0.97); Sp 0.16 (0.11, 0.24) |
| | | | | | | Pain not exacerbated by coughing | Se 0.90 (0.82, 0.95); Sp 0.13 (0.08, 0.20) |
| | | | | | | Traumatic onset | Se 0.48 (0.37, 0.58); Sp 0.50 (0.41, 0.59) |
| | | | | | | Prior history of LBP | Se 0.70 (0.59, 0.79); Sp 0.25 (0.18, 0.33) |
| | | | | | | Post surgery | Se 0.20 (0.13, 0.30); Sp 0.63 (0.54, 0.72) |
| | | | | | | Onset occupational | Se 0.14 (0.08, 0.23); Sp 0.72 (0.63, 0.79) |
| | | | | | | Positive work status | Se 0.34 (0.23 0.47); Sp 0.62 (0.51, 0.72) |
| | | | | | | Duration < 1 year | Se 0.21 (0.14 0.31); Sp 0.79 (0.71, 0.86) |
| | | | | | | Low back. hip buttocks pain | Se 0.98 (0.92 0.99); Sp 0.00 (0.00 0.03) |
| | | | | | | Bilateral pain | Se 0.76 (0.66 0.84); Sp 0.35 (0.27, 0.44) |

| | | | | | | | |
|---|---|---|---|---|---|---|---|
| | | | | | | Back pain with groin or thigh | Se 0.35 (0.25, 0.45); Se 0.68 (0.59, 0.76) |
| | | | | | | Pseudoradicular pain | Se 0.51 (0.41, 0.62); Sp 0.43 (0.34, 0.52) |
| | | | | | | Leg pain | Se 0.59 (0.48, 0.69); Sp 0.34 (0.26, 0.43) |
| | | | | | | Subjective pain ≥8/10 | Se 0.38 (0.28, 0.49); Sp 0.66 (0.57, 0.74) |
| | | | | | | Cramping pain above knees | Se 0.43 (0.33, 0.54); Sp 0.56 (0.47, 0.65) |
| | | | | | | Paresthesia | Se 0.74 (0.64, 0.82); Sp 0.16 (0.11, 0.24) |
| | | | | | | Low back pain stiffness | Se 0.58 (0.47, 0.68); Sp 0.38 (0.30, 0.48) |
| Manchikanti | 1999 | | N=120 | | | Traumatic onset | Se 0.54 (0.41, 0.66); Sp 0.47 (0.35, 0.59) |
| | | | | | | Previous surgery | Se 0.17 (0.09, 0.29); Sp 0.67 (0.55, 0.77) |
| | | | | | | Duration of pain > 4 years | Se 0.56 (0.42, 0.68); Sp 0.64 (0.52, 0.76) |
| Revel | 1998 | Tertiary | N=80 | | 75% response on Lidocaine block | Pain well relieved by recumbency | Se 0.92 (0.67, 0.99); Sp 0.24 (0.12, 0.42) |
| | | | | | | Pain not exacerbated by coughing | Se 1.00 (0.77, 1.00); Sp 0.35 (0.20, 0.53) |
| Scwharzer | 1994 | Secondary | N=176 | Adult patients with LBP referred by neurosurgeons, orthopaedic surgeons, and physiatrists | Double diagnostic block | Left groin pain referral | Se 0.21 (0.09, 0.43); Sp 0.84 (0.78, 0.90) |
| | | | | | | Right groin pain referral | Se 0.04 (0.01, 0.20); Sp 0.82 (0.75, 0.88) |
| | | | | | | Left buttock pain referral | Se 0.17 (0.09, 0.28); Sp 0.85 (0.76, 0.90) |
| | | | | | | Right buttock pain referral | Se 0.15 (0.08, 0.25); Sp 0.83 (0.74, 0.89) |

| | | | | | | | |
|---|---|---|---|---|---|---|---|
| | | | | | | Left thigh pain referral | Se 0.17 (0.09, 0.28); Sp 0.84 (0.77, 0.90) |
| | | | | | | Right thigh pain referral | Se 0.14 (0.08, 0.25); Sp 0.86 (0.79, 0.91) |
| | | | | | | Left calf pain referral | Se 0.17 (0.09, 0. 31); Sp ewq0.85 (0.77,0.89) |
| | | | | | | Right calf pain referral | Se 0.08 (0.03, 0.17); Sp 0.81 (0.73, 0.87) |
| | | | | | | Left foot pain referral | Se 0.26 (0.14, 0.43); Sp 0.86 (0.79, 0.91) |
| | | | | | | Right foot pain referral | Se 0.07 (0.02, 0.21); Sp 0.82 (0.75, 0.88) |
| Revel | 1992 | NA | N=40 | Patients suffering from LBP whatever the anatomic structure thought to be involved | 75% response on Lidocaine block | Relief with recumbency | Se 0.91 (0.72, 0.97); Sp 0.44 (0.25, 0.66) |
| | | | | | | Pain not worse with cough | Se 0.82 (0.62, 0.93); Sp 0.50 (0.29, 0.71) |
| **Diagnostic utility of patient history and physical examination data to detect spondylolysis and spondylolisthesis in athletes with low back pain: A systematic review (Grodahl, 2016)** | | | | | | | |
| PRIMARY STUDIES | | | | | | | |
| First author | Year of publication | Setting(s) of data collection | Number of participants (N) | Population | Reference standard | Index test | Diagnostic accuracy data |
| Gregg | 2009 | Sports medicine clinic | N=71 | All patients with LBP referred for a SPECT scan to confirm suspected diagnosis of spondylolysis | SPECT scan | Injured period < 3 months | Se 65.38; Sp 54.55 |
| | | | | | | Onset of symptoms (sudden) | Se 87.5; Sp 50.91 |
| | | | | | | Sports participation (yes) | Se 84.62; Sp 33.93 |
| Kalpakcioglu | 2009 | Hospital setting | N=130 | Patients with LBP and radiological diagnosis of spondylolisthesis | Antero-posterior, lateral, oblique and lateral flexion/extension | Pain localised to the low back | Se 23; Sp 76.67 |
| | | | | | | Sciatica | Se 61; Sp 26.67 |

| | | | | | | | |
|---|---|---|---|---|---|---|---|
| | | | | | radiograph flexion | Pain in the gluteal region or backside of the femur | Se 16; Sp 96.67 |

**Diagnostic performance of clinical tests for sacroiliac joint pain (Sivayogam, 2011)**

PRIMARY STUDIES

| First author | Year of publication | Setting(s) of data collection | Number of participants (N) | Population | Reference standard | Index test | Diagnostic accuracy data |
|---|---|---|---|---|---|---|---|
| Dreyfuss | 1996 | Tertiary | N=85 | Patients ≥ 18 years with non-specific, non-pregnancy related low back pain and/or buttock pain, with or without lower extremity symptoms | SIJB {with 1.5 ml lidocaine 2% and 0.5 ml corticosteroids; 90% pain relief on VAS) | Pain over SIJ | Se 0.85; Sp 0.08; +LR 0.9; -LR 1.87 |
| | | | | | | Pain over groin | Se 0.19; Sp 0.63; +LR 0.5; -LR 1.28 |
| | | | | | | Buttock pain | Se 0.80; Sp 0.14; +LR 0.9; -LR 1.42 |
| | | | | | | Sitting position | Se 0.03; Sp 0.90; +LR 0.3; -LR 1.07 |
| | | | | | | PSIS pointing | Se 0.76; Sp 0.47; +LR 1.4; -LR 0.51 |

**Systematic review of tests to identify the disc, SIJ or facet joint as the source of low back pain (Hancock, 2007)**

PRIMARY STUDIES

| First author | Year of publication | Setting(s) of data collection | Number of participants | Population | Reference standard | Index test | Diagnostic accuracy data |
|---|---|---|---|---|---|---|---|
| Manchikanti | 2000 | Secondary (One private pain management practice, in a non- university setting) | N=200 | Patients presenting to private pain management centre, with chronic LBP +/- lower extremity pain | Double diagnostic block | Pain reduced with recumbency | Se 94 (86, 98); Sp 17 (10,25); +LR 1.1 (1.0,1.2); -LR 0.39 (0.18,0.96) |
| | | | | | | Pain not increased with cough | Se 90 (82, 95); Sp 13 (8, 21); +LR 1.0 (0.9, 1.1); -LR 0.76 (0.34,1.66) |
| | | | | | | Traumatic onset | Se 48 (37,59); Sp 50 (41, 59); +LR 1.0 (0.7, 1.3); -LR 1.05 (0.80–1.37) |
| Manchikanti | 1999 | | N=120 | | | Traumatic onset | Se 54 (40, 67); Sp 47 (35, 60); +LR 1.01 (0.7, 1.4); -LR 0.99 (0.67, 1.44) |
| Revel | 1998 | Tertiary | N=42 | | 75% response on Lidocaine block | Pain reduced with recumbency | Se 89 (62, 99); Sp 25 (11, 44); +LR 1.2 (0.9, 1.6); -LR 0.43 (0.08–2.20) |

| | | | | | | | |
|---|---|---|---|---|---|---|---|
| | | | | Patients with LBP >3/12 referred for facet injection | | Pain not increased with cough | Se 96 (71, 100); Sp 35 (19, 55); +LR 1.5 (1.1, 2.0); -LR 0.10 (0.01,1.62) |
| Revel | 1992 | NA | N=40 | Patients suffering from LBP whatever the anatomic structure thought to be involved | 75% response on Lidocaine block | Pain reduced with recumbency | Se 89 (69, 98); Sp 45 (22, 69); +LR 1.6 (1.1,2.5); -LR 0.24 (0.07, 0.87) |
| | | | | | | Pain not increased with cough | Se 80 (59, 94); Sp 50 (27, 73); +LR 1.6 (1.0, 2.6); -LR 0.39 (0.15, 1.01) |

Table S10: Diagnostic accuracy of physical examination findings for non-specific LBP

| **Low back pain of disc, sacroiliac joint, or facet joint origin: a diagnostic accuracy systematic review (Han, 2023)** | | | | | | | |
|---|---|---|---|---|---|---|---|
| POOLED DATA | | | | | | | |
| First author | Year of publication | Setting(s) of data collection | Number of participants (N) / Number of studies (n) | Population | Reference standard | Index test | Diagnostic accuracy data (pooled estimates) |
| Han | 2023 | Secondary and tertiary care | N = ranged from 15 to 736; n=62 | Patients with low back pain without serious pathology such as cancer, infection, or fracture | Greater than or equal to 50% pain relief with double SIJ block | Gaenslen's test | Se 47.9 (38.7, 57.2); Sp 47.9 (37.5, 58.4); LR+ 0.85 (0.56, 1.28); LR- 1.12 (0.77, 1.62) |
| | | | | | | Sacral thrust test | Se 57.3 (45.9, 68.2); Sp 48.8 (37.9, 59.9); LR+ 1.13 (0.73, 1.75); LR- 0.87 (0.52, 1.44) |
| | | | | | | Thigh thrust test | Se 54.1 (48.1, 60.1); Sp 53.7 (44.9, 62.3); LR+ 1.13 (0.83, 1.55); LR- 0.91 (0.67, 1.22) |
| | | | | | | Compression test | Se 48.6 (31.9, 65.6); Sp 71.7 (56.5, 84.0); LR+ 1.79 (1.03, 3.11); LR- 0.74 (0.52, 1.05) |
| | | | | | | Patrick's test (FABER test) | Se 76.4 (70.2, 81.8); Sp 32.3 (23.3, 42.5); LR+ 1.05 (0.69, 1.60); LR- 0.86 (0.30, 2.48) |
| | | | | | | Distraction test | Se 41.7 (25.5, 59.2); Sp 80.4 (66.1, 90.6); LR+ 2.18 (1.08, 4.38); LR- 0.73 (0.54, 0.99) |
| | | | | | | Gillet's test | Se 67.5 (56.4, 77.3); Sp 45.5 (31.2, 60.2); LR+ 1.01 (0.80, 1.28); LR- 1.08 (0.75, 1.55) |
| | | | | | | Absence of midline LBP | Se 24.6 (14.1, 37.8); Sp 34.3 (27.2, 42.0); LR+ 2.41 (1.89, 3.07); LR- 0.35 (0.12, 1.01) |
| PRIMARY STUDIES | | | | | | | |

| First author | Year of publication | Setting(s) of data collection | Number of participants (N) | Population | Reference standard | Index test | Diagnostic accuracy data |
|---|---|---|---|---|---|---|---|
| Mekhail | 2021 | Tertiary | N=199 | Patients with LBP evaluated by diagnostic SIJ injections | Greater than or equal to 50% pain relief with double SIJ block | Mekhail test | 80.4 (73.3 to 86.3) 26.8 (14.2 to 42.9) 1.10 (0.90 to 1.34) 0.73 (0.40 to 1.33) |
| | | | | | | Patrick test | 81.6 (74.7 to 87.3) 43.9 (28.5 to 60.3) 1.46 (1.10 to 1.93) 0.42 (0.26 to 0.67) |
| | | | | | | Thigh thrust test | 53.2 (45.1 to 61.1) 48.8 (32.9 to 64.9) 1.04 (0.74 to 1.45) 0.96 (0.67 to 1.37) |
| | | | | | | Mekhail/Patrick test | 94.3 (89.5 to 97.4) 17.1 (7.2 to 32.1) 1.14 (0.99 to 1.31) 0.34 (0.13 to 0.84) |
| | | | | | | Mekhail/thigh thrust test | 89.9 (84.1 to 94.1) 14.6 (5.6 to 29.2) 1.05 (0.92 to 1.21) 0.69 (0.29 to 1.66) |
| | | | | | | Patrick/thigh thrust test | 89.2 (83.3 to 93.6) 29.3 (16.1 to 45.5) 1.26 (1.03 to 1.55) 0.37 (0.19 to 0.71) |
| Nejati | 2020 | Secondary | N=48 | Patients with LBP evaluated by diagnostic SIJ injections | Greater than or equal to 50% pain relief with double SIJ block | FABER test | Se 71.8 (55.1, 85.0); Sp 66.7 (29.9, 92.5); LR+ 2.15 (0.84, 5.54); LR- 0.42 (0.21, 0.84) |
| | | | | | | Thigh thrust test | Se 74.4 (57.9, 87.0); Sp 44.4 (13.7, 78.8); LR+ 1.34 (0.73, 2.47); LR- 0.58 (0.23, 1.43) |
| | | | | | | Gaenslen test | Se 38.5 (23.4, 55.4); Sp 33.3 (7.5, 70.1); LR+ 0.58 (0.31, 1.06); LR- 1.85 (0.71, 4.81) |
| | | | | | | Yeoman test | Se 64.1 (47.2, 78.8); Sp 33.3 (7.5, 70.1); LR+ 0.96 (0.57, 1.61); LR- 1.08 (0.39, 2.97) |

| | | | | | | | |
|---|---|---|---|---|---|---|---|
| | | | | | | Gillet test | Se 98.7 (88.8, 100.00); Sp 5.3 (0.0, 39.7); LR+ 1.04 (0.89, 1.22) LR- 0.24 (0.01, 11.36) |
| | | | | | | Forward flexion test | Se 98.7 (88.8, 100.00); Sp 5.3 (0.0, 39.7); LR+ 1.04 (0.89, 1.22) LR- 0.24 (0.01, 11.36) |
| Lewinnek | 1986 | NA | N=21 | Patients with LBP | Greater than or equal to 80% pain relief with double blocks | Pain on extension (for pain originating from the facet joint) | Se 73.3 (44.9, 92.2); Sp 80.0 (28.4, 99.5); LR+ 3.67 (0.62, 21.73); LR- 0.33 (0.13, 0.86) |
| | | | | | | SLR (pain originating from the facet joint) | Se 66.7 (22.3, 95.7); Sp 76.9 (46.2, 95.0); LR+ 2.89 (0.92, 9.06); LR- 0.43 (0.13, 1.40) |

**Reliability and validity of manual palpation for the assessment of patients with low back pain: a systematic and critical review (Nolet, 2021)**

PRIMARY STUDIES

| First author | Year of publication | Setting(s) of data collection | Number of participants (N) | Population | Reference standard | Index test | Diagnostic accuracy data |
|---|---|---|---|---|---|---|---|
| Adelmanesh | 2016 | NA | N=777 | Adult patients with LBP with or without radiculopathy of any duration | Multidisciplinary panel of experts based on examination of clinical evaluations, MRI, and if needed, electrodiagnostic testing | Palpation of the superior-lateral quadrant of the gluteal muscle to identify GTrP representing the combination of tenderness, taut band and pain | Se 74.1 (67.7, 80.3); Sp 91.4 (86.8, 96); +LR 8.6; -LR 0.28; PPV 91.9 (87.6, 96.3); NPV 72.7 (66.1, 79.3); ROC curve 0.827 (0.781, 0.874) |
| Koppenhaver | 2014 | NA | N=51 | Adult patients with chronic LBP, with modified ODI ≥20/100 | Spinal stiffness was quantified using a mechanized indentation device. | Palpation of spinal stiffness: the spinous processes of L1-L5 palpated with the subject lying prone | Se 0.45 (0.28, 0.62); Sp 0.38 (0.21, 0.59); +LR 0.69 (0.37, 1.31); -LR 1.38 (0.82, 2.33) |

| Evidence and recommendations for the use of segmental motion testing for patients with LBP - A systematic review (Stolz, 2020) | | | | | | | |
|---|---|---|---|---|---|---|---|
| PRIMARY STUDIES | | | | | | | |
| First author | Year of publication | Setting(s) of data collection | Number of participants (N) | Population | Reference standard | Index test | Diagnostic accuracy data |
| Fritz | 2005 | Tertiary | N=49 | Adult patients with a chief complaint of LBP of any duration | F/E radiographs | PAIVMs | Hypomobility: Se 0.43 (0.27, 0.61); Sp 0.95 (0.77, 0.99); LR+: 9.00 (1.3, 63.9); LR-: 0.60 (0.43, 0.84) Hypermobility: Se 0.46 (0.30, 0.64); Sp 0.81 (0.60, 0.92); LR+: 2.42 (0.93, 6.4) LR- 0.66 (0.44, 0.99) |
| Abbot | 2005 | Physical therapy clinics | N=123 | Patients with a new episode of LBP or history of LBP at least three months before recruitment | F/E radiographs | PAIVMs | Rotation LSI (by segment): Se 0.17 (0.03, 0.56); Sp 0.95 (0.92, 0.96); LR+: 3.10 (0.50, 19.39); LR- : 0.88 (0.62, 1.26); Translation LSI (by segment): Se 0.19 (0.07, 0.43); Sp 0.95 (0.92, 0.97); LR 3.73 (1.24, 11.23); LR- 0.86 (0.68, 1.08) |
| | | | | | | Flex PPIVMs | Rotation LSI (by segment): Se 0.07 (0.01, 0.44); Sp 0,99 (0.98, 1,00); LR+ 12.71 (0.67, 241.27); LR- 0.93 (0.76, 1.15); Translation LSI (by segment): Se 0.03 (0.00, 0.22); Sp 0.99 (0.92, 1.00); LR+ 4.82 (0.24, 96.82); LR- 0.98 (0.90, 1.06) |
| | | | | | | Ext PPVMs | Rotation LSI (by segment) Se 0.07 (0.01, 0.44); Sp 0.99 (0.97, 1.00); LR+ 6.75 (0.39, 110.76); LR- 0.94 (0.76, 1.15); Translation LSI (by segment): Se 0.03 (0.00, 0.25); Sp 1.00 (1.00, 1.00); LR+ 26.8 (0.55, 1305.5); LR- 0.97 (0.88, 1.06) |
| Philips and Twoney | 1996 | NA | N=63 | NA | Spinal anaesthetic block | PAIVMs and PPIVMs | Se 0.53; Sp 0.80 |

| Systematic review of patient history and physical examination to diagnose chronic low back pain originating from the facet joints (Maas, 2017) | | | | | | | |
|---|---|---|---|---|---|---|---|
| PRIMARY STUDIES | | | | | | | |
| First author | Year of publication | Setting(s) of data collection | Number of participants (N) | Population | Reference standard | Index test | Diagnostic accuracy data |
| Laslett | 2006 | Secondary | N=120 | Adult patients, of either gender, suffering from CLBP | ZJ block 75% reduction standard | Extension rotation test | Se 0.86 (0.69, 0.94); Sp 0.22 (0.14, 0.32) |
| | | | | | ZJ block 80% reduction standard | | Se 0.87 (0.69,0.96); Sp 0.22 (0.14, 0.32) |
| | | | | | ZJ block 85% reduction standard | | Se 0.91 (0.72, 0.97); Sp 0.22 (0.15, 0.31) |
| | | | | | ZJ block 90% reduction standard | | Se 0.94 (0.72, 0.99); Sp 0.22 (0.15, 0.31) |
| | | | | | ZJ block 95% reduction standard | | Se 1.00 (0.76,1.00); Sp 0.22 (0.15, 0.31) |
| | | | | | ZJ block 95% reduction standard | Absence of centralization phenomenon | Se 1.00 (0.74., 1.00); Sp 0.17 (0.11., 0.27) |
| Gonzales | 2004 | Pain clinic | N=150 | Adult patients with lumbar zygapophysial joint pain | Double diagnostic blocks (corresponding facet joints, combination of local anesthetic and corticosteroid) | Lumbar facet sign | Se 0.95 (0.84, 0.99); Sp unable to estimate |
| Young | 2003 | Secondary (Private radiology practice) | N=81 | Adult patients with chronic lumbar or lumbopelvic pain who were referred to a private radiology practice | ≥80% pain relief after lumbar zygapophysial joint injection | Centralization | Se 0.00 (0.00, 0.22); Sp 0.89 (0.56, 0.98) |
| | | | | | | Non-centralization | Se 1.00 (0.78, 1.00); Sp 0.11 (0.02, 0.44) |
| Manchikanti | 2000 | Secondary (One private pain management | N=200 | Patients presenting to private pain management centre, | Double diagnostic block | Pain not exacerbated by forward flexion | Se 0.15 (0.09,0.25); Sp 0.82 (0.74, 0.88) |

| | | | | | | | |
|---|---|---|---|---|---|---|---|
| | | practice, in a non- university setting) | | with chronic LBP +/- lower extremity pain | | Pain not exacerbated by deflexion | Se 0.55 (0.44,0.65); Sp 0.48 (0.39, 0.57) |
| | | | | | | Pain not exacerbated by hyperextension | Se 0.10 (0.05,0.18); Sp 0.86 (0.79, 0.91) |
| | | | | | | Pain not exacerbated by extension/rotation | Se 0.68 (0.57,0.77); Sp 0.30 (0.23, 0.39) |
| | | | | | | Pain with flexion | Se 0.85 (0.75, 0.91); Sp 0.18 (0.12, 0.26) |
| | | | | | | Pain with deflexion | Se 0.57 (0.46, 0.67); Sp 0.46 (0.37, 0.55) |
| | | | | | | Pain with extension | Se 0.88 (0.79, 0.93); Sp 0.14 (0.09, 0.21) |
| | | | | | | Pain with lateral rotation | Se 0.68 (0.57, 0.77); Sp 0.30 (0.23, 0.39) |
| | | | | | | Pain with sitting and bending | Se 0.49 (0.38, 0.59); Sp 0.56 (0.47, 0.65) |
| | | | | | | Normal gait | Se 0.98 (0.92, 0.99); Sp 0.10 (0.06, 0.17) |
| | | | | | | Muscle spasm | Se 0.48 (0.37, 0.58); Sp 0.46 (0.37, 0.55) |
| | | | | | | Paravertebral tenderness | Se 0.93 (0.85, 0.97); Sp 0.13 (0.08, 0.20) |
| | | | | | | Pain increased by cough/valsalva | Se 0.14 (0.08, 0.23); Sp 0.86 (0.79, 0.91) |
| | | | | | | Back pain with SLR | Se 0.49 (0.39, 0.60); Sp 0.37 (0.29, 0.46) |
| | | | | | | Negative neurological exam | Se 0.91 (0.83, 0.95); Sp 0.25 (0.18, 0.34) |

| | | | | | | | |
|---|---|---|---|---|---|---|---|
| Revel | 1998 | Tertiary | N=80 | Adult patients with lumbar zygapophysial joint pain | 75% response on Lidocaine block | Pain not exacerbated by forward flexion | Se 1.00 (0.77, 1.00); Sp 0.48 (0.31, 0.66) |
| | | | | | | Pain not exacerbated by rising from flexion | Se 1.00 (0.77, 1.00); Sp 0.59 (0.41, 0.74) |
| | | | | | | Pain not exacerbated by hyperextension | Se 0.92 (0.67,0.98); Sp 0.62 (0.44, 0.77) |
| | | | | | | Pain not exacerbated by extension/rotation | Se 0.76 (0.50, 0.92); Sp 0.48 (0.31, 0.66) |
| Scwharzer | 1994 | Secondary | N=176 | Adult patients with LBP referred by neurosurgeons, orthopaedic surgeons, and physiatrists | Double diagnostic block | Absence of pain on combined rotation and extension | Se 0.18 (0.12, 0.25); Sp 1.0 (0.81, 1.0) |
| Revel | 1992 | NA | N=40 | Patients suffering from LBP whatever the anatomic structure thought to be involved | 75% response on Lidocaine block | Pain not worse with forward flexion | Se 0.64 (0.43, 0.80); Sp 0.78 (0.55, 0.91) |
| | | | | | | Pain not worse with raising from forward flexion | Se 0.77 (0.57, 0.90); Sp 0.56 (0.34, 0.75) |
| | | | | | | Pain not worse with hyperextension | Se 0.55 (0.35,0.73); Sp 0.72 (0.49, 0.88) |
| | | | | | | Pain not worse with extension/rotation | Se 0.68 (0.47, 0.84); Sp 0.78 (0.55, 0.91) |

| Diagnostic utility of patient history and physical examination data to detect spondylolysis and spondylolisthesis in athletes with low back pain: A systematic review (Grodahl, 2016) | | | | | | | |
|---|---|---|---|---|---|---|---|
| **PRIMARY STUDIES** | | | | | | | |
| First author | Year of publication | Setting(s) of data collection | Number of participants (N) | Population | Reference standard | Index test | Diagnostic accuracy data |
| Anh and Juhn | 2015 | Pain management clinic | N=86 | Patients with LBP/lumbar radicular pain | Lumbar lateral radiography | Step deformiy (palpation) | Se 81.3; Sp 89.1; +LR 7.43; -LR 0.21 |
| Ferrari | 2014 | Pain management clinic | N=119 | Patients with LBP with/without referred pain and a diagnosis of spondylolisthesis | Radiographs, MRI or CT | ASLR | Se 64; Sp 45; +LR 1.16; -LR 0.80 |
| | | | | | | Prone instability test | Se 44; Sp 45; +LR 0.80; -LR 1.24 |
| | | | | | | Passive lumbar extension | Se 43; Sp 86; +LR 3.07; -LR 0.66 |
| | | | | | | Abberant movement | Se 41; Sp 77; +LR 1.78; -LR 0.76 |
| Sundell | 2013 | Physiotherapy clinic | N=25 | Patients practicing 6 h of sports/week and >3 weeks of LBP, hindering their ADL or physical activity, with suspected spondylolisis | MRI and CT | OLHET | Se 61.54; Sp 0; +LR 0.62 |
| | | | | | | Prone back extension with fixed pelvis test | Se 46.15; Sp 33.33; +LR 0.69; -LR 1.62 |
| | | | | | | Coin test | Se 84.62; Sp 16.67; +LR 1.02; -LR 0.92 |
| | | | | | | Percussion test with reflex hammer | Se 38.76; Sp 50; +LR 0.77; -LR 1.12 |
| | | | | | | Rocking test | Se 69.23; Sp 25; +LR 0.92; -LR 1.23 |
| | | | | | | Sacrum nutation test | Se 23; Sp 58.33; +LR 0.55; -LR 1.32 |
| | | | | | | HOOK test | Se 46.15; Sp 75; +LR 1.85; -LR 0.72 |
| | | | | | | MCI control test | Se 69.23; Sp 50; +LR 1.38; -LR 0.62 |
| Gregg | 2009 | Sports medicine clinic | N=71 | All patients with LBP referred for a SPECT scan to confirm | SPECT scan | OLHET | Se 73; Sp 17.2 |

| | | | | | | | |
|---|---|---|---|---|---|---|---|
| | | | | suspected diagnosis of spondylolysis | | | |
| Kalpakcioglu | 2009 | Hospital setting | N=130 | Patients with LBP and radiological diagnosis of spondylolisthesis | Antero-posterior, lateral, oblique and lateral flexion/extension radiograph flexion | Gait disorder | Se 5; Sp 93.33; +LR 0.75; -LR 1.02 |
| | | | | | | Weak/ dropping abdominal wall | Se 99; Sp 40; +LR 1.65; -LR 0.03 |
| | | | | | | Paravertebral muscles hypertrophy | Se 65; Sp 70; +LR 2.17; -LR 0.50 |
| | | | | | | Paravertebral muscles spasm | Se 87; Sp 13.33; +LR 1.0; -LR 0.98 |
| | | | | | | Increase in lumbar lordosis | Se 58; Sp 63; +LR 1.58; -LR 0.66 |
| | | | | | | Signs of slipping (inspection) | Se 21; Sp 100; -LR 0.29 |
| | | | | | | Step deformiy (palpation) | Se 88; Sp 100; -LR 0.12 |
| | | | | | | Hamstring muscle spasm | Se 27; Sp 96.67; +LR 8.10; -LR 0.76; |
| | | | | | | Contracting hamstring muscle | Se 1; Sp 90; +LR 0.10; -LR 1.10 |
| | | | | | | Z posture | Se 2; Sp 100; -LR 1.10 |
| | | | | | | Lumbar flexion | Se 19; Sp 3.33; +LR 0.20; -LR 24.3 |
| | | | | | | Lumbar extension | Se 79; Sp 66.67; +LR 2.37; -LR 0.31 |
| | | | | | | Lumbar lateral flexion | Se 46; Sp 83.33; +LR 2.76; -LR 0.65 |
| | | | | | | Lumbar rotation | Se 10; Sp 96.67; +LR 3; -LR 0.93 |
| | | | | | | SLR | Se 10; Sp 90; +LR 1; -LR 1 |
| | | | | | | ASLR | Se 87; Sp 76.67; 3.73; -LR 0.17 |
| | | | | | | Femoral stretch test | Se 14; Sp 96.67; +LR 4.20; -LR 0.89 |
| | | | | | | Achilles reflex | Se 13; Sp 93.33; +LR 2.40; -LR 0.95 |
| | | | | | | Patellar reflex | Se 8; Sp 96.67; +LR 0.30; -LR 1.02 |

| | | | | | | | |
|---|---|---|---|---|---|---|---|
| | | | | | | Loss of strength | Se 1; Sp 96.67; +LR 0.3; -LR 1.02 |
| | | | | | | Sensorial change | Se 2; Sp 100; -LR 0.98 |
| | | | | | | Walking distance < 250 m | Se 74; Sp 60; +LR 1.85; -LR 0.43 |
| Masci | 2006 | Sports medicine clinic | N=82 | Patients engaged in regular activity, symptoms of LBP <6 months, provisional diagnosis of active spondylolysis | Bone scintigraphy with SPECT/CT | OLHET | Left leg: Se 50, Sp 32.4; +LR 0.74; -LR 1.54; Right leg: Se 55; Sp 45.4; +LR 1.01; -LR 0.98 |
| Collaer | 2006 | Hospital/Sports medicine clinic | N=30 | Patients with LBP and/or radiculopathy | Lumbar lateral radiography | Step deformiy (palpation) | Se 60; Sp 87; +LR 4.68; -LR 0.46 |
| Möller | 2000 | Hospital setting | N=111 | Patients with lumbar isthmic spondylolisthesis of all grades with at least 1 year of low back pain or sciatica and severely restricted functional ability | Radiographs | EHL-reduced power | Se 6.31; +LR 0.06 |
| | | | | | | SLR | Se 11.71; +LR 0.12 |
| | | | | | | Femoral stretch test | Se 1.80; +LR 0.02 |
| | | | | | | Lateral flexion | Se 42.34; +LR 0.42 |
| | | | | | | Hamstring tightness | Se 20.72; +LR 0.21 |
| | | | | | | Achilles reflex | Se 5.41; +LR 0.05 |
| | | | | | | Patellar reflex | Se 4.50; +LR 0.05 |
| | | | | | | Sensorial change | Se 23.42; +LR 0.43 |
| | | | | | | Lumbosacral tenderness | Se 66.67; +LR 0.67 |
| **A literature review of clinical tests for lumbar instability in low back pain: validity and applicability in clinical practice (Ferrari, 2015)** | | | | | | | |
| PRIMARY STUDIES | | | | | | | |
| First author | Year of publication | Setting(s) of data collection | Number of participants (N) | Population | Reference standard | Index test | Diagnostic accuracy data |
| Fritz | 2005 | Tertiary | N=38 | Patients with LBP with or without buttock or leg pain | Dynamic X-Ray (Flexion/extension) | Aberrant movement pattern: Painful arc on flexion; | Se 0.18 (0.08, 0.36); Sp 0.95 (0.77, 0.99); +LR 3.75 (0.47, 29.75); -LR (0.71, 1.05) |

| | | | | | | | |
|---|---|---|---|---|---|---|---|
| | | | | | | Painful arc on return; Instability catch; Gower sign ("thigh climbing"); Reverse lumbopelvic rhythm. (Positive test when at least 1 of the previous 5 signs was present.) | |
| | | | | | | Prone Instability Test (Positive test when pain provoked during the first part of the test decreases when the test is repeated with the legs off the floor) | Se 0.71 (0.53, 0.85); Sp 0.57 (0.37, 0.76); +LR 1.67 (0.97, 2.88); -LR 0.50 (0.97, 2.88) |
| | | | | | | Posterior Shear Test: Positive test if familiar symptoms are provoked. | Se 0.50 (0.34,0.66); Sp 0.48 (0.28, 0.68); +LR 0.96 (0.56, 1.63); -LR 1.05 (0.60, 1.85) |
| Kasai | 2006 | NA | N=122 | Patients with lumbar degenerative diseases (89 lumbar spinal canal stenosis; 21 lumbar spondylolisthesis; 12 lumbar degenerative scoliosis) | Dynamic x-ray: flexion-extension films of the lumbar spine, lateral vision. | Passive lumbar extension test | Se 0.84 (0.7, 0.93); Sp 0.90 (0.82, 0.95); +LR 8.84 (4.51,17.34); -LR 0.18 (0.08, 0.37) |
| | | | | | | Instability catch sign | Se 0.26 (0.15,0.42); Sp 0.86 (0.77, 0.92); +LR 1.84 (0.87, 3.89); -LR 0.86 (0.87, 1.06) |

| (Alqarni, 2011) | | | | | | | |
|---|---|---|---|---|---|---|---|
| PRIMARY STUDIES | | | | | | | |
| First author | Year of publication | Setting(s) of data collection | Number of participants (N) | Population | Reference standard | Index test | Diagnostic accuracy data |
| Kasai | 2006 | NA | N=122 | Patients with lumbar degenerative diseases (89 lumbar spinal canal stenosis; 21 lumbar spondylolisthesis; 12 lumbar degenerative scoliosis) | Dynamic x-ray: flexion-extension films of the lumbar spine, lateral vision. | Painful catch sign | Se 37 (22, 54); Sp 73 (62, 82); +LR 1.4 (0.8, 2.3); -LR 0.9 (0.7, 1.1) |
| | | | | | | Apprehension sign | Se 18 (8, 35); Sp 88 (79,94); +LR 1.6 (0.6, 3.8); -LR 0.9 (0.8, 1.1) |
| Maigne | 2003 | Tertiary | N=42 | Patients with chronic LBP | Radiographic diagnosis of translational LSI (flexion-extension radiographs) | Sit-to-stand test (pain upon sitting down and relieved by standing up) | Se 31 (10, 61); Sp 100 (85, 100); -LR 0.7 (0.5, 1.0) |

| **Diagnostic performance of clinical tests for sacroiliac joint pain (Sivayogam, 2011)** | | | | | | | |
|---|---|---|---|---|---|---|---|
| PRIMARY STUDIES | | | | | | | |
| First author | Year of publication | Setting(s) of data collection | Number of participants (N) | Population | Reference standard | Index test | Diagnostic accuracy data |
| Laslett | 2005 | Secondary | N=48 | Patients with buttock pain, with or without lumbar or lower extremity symptoms | Double SIJB with 1.5 ml lidocaine and bupivacaine; pain provocation followed by 80% pain relief on NRS1 | Distraction | Se 0.60 (0.36, 0.80); Sp 0.81 (0.65, 0.91); +LR 3.20; -LR 0.49 |
| | | | | | | Compression | Se 0.69 (0.44, 0.86); Sp 0.69 (0.51); +LR 2.20; - LR 0.46 |
| | | | | | | Thigh thrust | Se 0.88 (0.64, 0.97); Sp 0.69 (0.82); +LR 2.80; -LR 0.18 |
| | | | | | | Gaenslen's right | Se 0.53 (0.30, 0.75); Sp 0.71 (0.53, 0.84); +LR 1.84; -LR 0.66 |
| | | | | | | Gaenslen's left | Se 0.50 (0.27,0.73); Sp 0.77 (0.60, 0.89); +LR 2.21; -LR 0.65 |

| | | | | | | | |
|---|---|---|---|---|---|---|---|
| | | | | | | Sacral thrust | Se 0.63 (0.39, 0.82); Sp 0.75 (0.58, 0.87); +LR 2.50; -LR 0.50 |
| Broadhurst and Bond | 1998 | NA | N=40 | NA | SIJB with 4 ml lidocaine 1% or 0.9% NaCl; 70% pain relief on VAS | Patrick's sign | Se 0.77; Sp 1.00 |
| | | | | | | Thigh thrust | Se 0.80; Sp 1.00 |
| | | | | | | Resisted abduction | Se 0.87; Sp 1.00 |
| Dreyfuss | 1996 | Tertiary | N=85 | Patients ≥ 18 years with non-specific, non-pregnancy related low back pain and/or buttock pain, with or without lower extremity symptoms | SIJB {with 1.5 ml lidocaine 2% and 0.5 ml corticosteroids; 90% pain relief on VAS) | Gillet test | Se 0.43; Sp 0.68; +LR 1.3; -LR 0.83 |
| | | | | | | Thigh thrust | Se 0.36; Sp 0.50; +LR 0.7; -LR 1.28 |
| | | | | | | Patrick's sign | Se 0.69; Sp 0.16; +LR 0.8; -LR 1.93 |
| | | | | | | Gaenslen's test | Se 0.71; Sp 0.26; +LR 1.0; -LR 1.11 |
| | | | | | | Sacral thrust | Se 0.53; Sp 0.29; +LR 0.8; -LR 1.62 |
| | | | | | | Sacral spring | Se 0.75; Sp 0.35; +LR 1.2; -LR 0.71 |
| | | | | | | Sacral sulcus | Se 0.95; Sp 0.09; +LR 1.0; -LR 0.55 |
| **Systematic review of tests to identify the disc, SIJ or facet joint as the source of low back pain (Hancock, 2007)** | | | | | | | |
| PRIMARY STUDIES | | | | | | | |
| First author | Year of publication | Setting(s) of data collection | Number of participants | Population | Reference standard | Index test | Diagnostic accuracy data |
| Laslett | 2006 | Secondary | N=120 | Adult patients, of either gender, suffering from CLBP | NA | No centralisation | Se 96 (65, 100); Sp 16 (8, 28); +LR 1.1 (1.0, 1.4); -LR 0.28 (0.02, 4.43) |
| Laslett | 2005 | Secondary | N=48 | Patients with buttock pain, with or without lumbar or lower extremity symptoms | Double SIJB with 1.5 ml lidocaine and bupivacaine; pain provocation followed by 80% pain relief on NRS1 | Thigh thrust | Se 69 (45, 87); Sp 64 (44,81); +LR 1.9 (1.1,3.3); -LR 0.49 (0.24,0.97) |
| | | | | | | Sacral thrust | Se 51 (36,66); Sp 40 (25, 57); +LR 0.9 (0.6, 1.2); -LR 1.22 (0.76, 1.96) |
| Young | 2003 | Secondary (Private radiology practice) | N=81 | Adult patients with chronic lumbar or lumbopelvic pain who were referred to a private radiology practice | ≥80% pain relief after lumbar zygapophysial joint injection | No centralisation | Se 97 (73, 100); Sp 15 (1, 50); +LR 1.1 (0.9, 1.5); -LR 0.22 (0.01, 4.93) |

| | | | | | | | |
|---|---|---|---|---|---|---|---|
| Manchikanti | 2000 | Secondary (One private pain management practice, in a non- university setting) | N=200 | Patients presenting to private pain management centre, with chronic LBP +/- lower extremity pain | Double diagnostic block | Pain not increased with forward flexion | Se 16 (9,25); Sp 82 (73, 88); +LR 0.9 (0.5,1.6); -LR 1.03 (0.91,1.17) |
| | | | | | | Pain not increased with rising from flexion | Se 55 (44,65); Sp 48 (39, 58); +LR 1.1 (0.8,1.4); -LR 0.94 (0.70, 1.26) |
| | | | | | | Pain not increased with hyperextension | Se 10 (5,18); Sp 86 (78,92); +LR 0.7 (0.3,1.5); +LR 1.05 (0.95,1.16) |
| | | | | | | Pain not increased with extension / rotation | Se 68 (57,77); Sp 30 (22, 40); +LR 1.0 (0.8, 1.2); -LR 1.07 (0.71, 1.61) |
| Revel | 1998 | Tertiary | N=42 | Patients with LBP >3/12 referred for facet injection | 75% response on Lidocaine block | Pain not increased with forward flexion | Se 89 (62, 99); Sp 25 (11, 44); +LR 1.2 (0.9, 1.6); -LR 0.43 (0.08–2.20) |
| | | | | | | Pain not increased with rising from flexion | Se 96 (71, 100); Sp 35 (19, 55); +LR 1.5 (1.1,2.0); -LR 0.10 (0.01–1.62) |
| | | | | | | Pain not increased with hyperextension | Se 96 (71, 100); Sp 48 (30, 67); +LR 1.9 (1.3, 2.7); -LR 0.07 (0.01, 1.15) |
| | | | | | | Pain not increased with extension / rotation | Se 96 (71, 100); Sp 58 (39, 76); +LR 2.3 (1.5, 3.6); -LR 0.06 (0.00, 0.97) |
| Dreyfuss | 1996 | Tertiary | N=85 | Patients ≥ 18 years with non-specific, non-pregnancy related low back pain and/or buttock pain, with or without lower extremity symptoms | SIJB {with 1.5 ml lidocaine 2% and 0.5 ml corticosteroids; 90% pain relief on VAS) | Sacral thrust | Se 51 (36, 66); Sp 40 (25,57), +LR 0.9 (0.6, 1.2); -LR 1.22 (0.76, 1.96) |
| Revel | 1992 | NA | N=40 | Patients suffering from LBP whatever the | 75% response on Lidocaine block | Pain not increased with forward flexion | Se 63 (41, 82); Sp 76 (52, 92); +LR 2.7 (1.1, 6.3); -LR 0.48 (0.27, 0.87) |

| | | | | | | | |
|---|---|---|---|---|---|---|---|
| | | | | anatomic structure thought to be involved | | Pain not increased with rising from flexion | Se 76 (54, 91); Sp 55 (31, 78); +LR 1.7 (1.0, 2.9); -LR 0.43 (0.19, 1.00) |
| | | | | | | Pain not increased with hyperextension | Se 54 (33, 75); Sp 71 (46, 89); +LR 1.9 (0.8, 4.2); -LR 0.64 (0.38, 1.09) |
| | | | | | | Pain not increased with extension / rotation | Se 68 (45, 85); Sp 76 (52, 92); +LR 2.8 (1.2, 6.7); -LR 0.43 (0.23, 0.81) |
| **Accuracy of spinal orthopaedic tests: a systematic review (Simpson, 2006)** | | | | | | | |
| PRIMARY STUDIES | | | | | | | |
| First author | Year of publication | Setting(s) of data collection | Number of participants | Population | Reference standard | Index test | Diagnostic accuracy data |
| Laslett | 2003 | Tertiary | N=48 | Patients with buttock pain, with or without lumbar or lower extremity symptoms, referred to a private radiology practice | SIJB | SI Compression | Se 91; Sp 83 |
| | | | | | | SI Distraction | Se 92; Sp 83 |
| | | | | | | Thigh trust | Se 93; Sp 83 |
| | | | | | | Gaenslen | Se 94; Sp 83 |
| | | | | | | Sacral Thrust | Se 95; Sp 83 |
| Leboeuf | 1990 | Primary | N=68 | Patient with lumbosacral pain attending a chiropractic clinic | NA | Fabere | Se 10; Sp 86 |
| | | | | | | SI Aggravation | Se 20; Sp 59 |
| | | | | | | Ely | Se 44; Sp 83 |
| | | | | | | Yoeman | Se 46; Sp 72 |
| | | | | | | Sacral Base Spring | Se 33; Sp 59 |

Table S11: Diagnostic accuracy of clinical diagnostic support tools for the diagnosis of non-specific LBP

| **Low back pain of disc, sacroiliac joint, or facet joint origin: a diagnostic accuracy systematic review (Han, 2023)** | | | | | | | |
|---|---|---|---|---|---|---|---|
| POOLED DATA | | | | | | | |
| First author | Year of publication | Setting(s) of data collection | Number of participants (N) / Number of studies (n) | Population | Reference standard | Index test | Diagnostic accuracy data (pooled estimates) |
| Han | 2023 | Secondary and tertiary care | N = ranged from 15 to 736 / n=62 | Patients with low back pain without serious pathology such as cancer, infection, or fracture | Greater than or equal to 50% pain relief with double SIJ block | 3 or more positive SIJ pain provocation test | Se 80.5 (72.0, 87.4); Sp 68.1 (60.4, 75.2); LR+ 2.44 (1.50, 3.98); LR- 0.31 (0.21, 0.47) |
| **Systematic review of patient history and physical examination to diagnose chronic low back pain originating from the facet joints (Maas, 2017)** | | | | | | | |
| PRIMARY STUDIES | | | | | | | |
| First author | Year of publication | Setting(s) of data collection | Number of participants (N) | Population | Reference standard | Index test | Diagnostic accuracy data |
| Laslett | 2006 | Secondary | N=120 | Adult patients, of either gender, suffering from CLBP | ZJ block 95% reduction standard | Clinical prediction rule (CPR) 1 | Se 1.00 (0.70, 1.00); Sp 0.87 (0.70, 0.93) |
| | | | | | | Clinical prediction rule (CPR) 2 | Se 1.00 (0.76, 1.00); Sp 0.36 (0.27, 0.45) |
| | | | | | | Clinical prediction rule (CPR) 3 | Se 1.00 (0.76,1.00); Sp 0.26 (0.19, 0.35) |
| | | | | | | Clinical prediction rule (CPR) 4 | Se 1.00 (0.76, 1.00); Sp 0.50 (0.41, 0.60) |
| | | | | | | Clinical prediction rule (CPR) 5 | Se 0.85 (0.58,0.96); Sp 0.91 (0.84, 0.95) |
| Laslett | 2004 | Secondary | N=151 | Patients with low back pain with or without lower extremity | ZJ block 75% reduction standard | Revel's criteria | Se 0.11 (0.02, 0.29); Sp 0.91 (0.83, 0.96) |

| | | | | | | | |
|---|---|---|---|---|---|---|---|
| | | | | symptoms, referred to a private radiology practice | ZJ block 100% reduction standard | Revel's criteria. | Se 0.06 (0.01, 0.28); Sp 0.93 (0.86, 0.97) |
| Manchikanti | 2000 | Secondary (One private pain management practice, in a non- university setting) | N=200 | Patients presenting to private pain management centre, with chronic LBP +/- lower extremity pain | Double diagnostic block | Revel's criteria | Se 0.13 (0.07, 0.22); Sp 0.84 (0.76, 0.90) |
| | | | | | | > 3 inappropriate symptoms | Se 0.24 (0.16, 0.34); Sp 0.68 (0.59, 0.76) |
| | | | | | | > 3 inappropriate signs | Se 0.54 (0.43, 0.64); Sp 0.59 (0.49, 0.67) |
| Revel | 1998 | Tertiary | N=80 | Patients with LBP >3/12 referred for facet injection | 75% response on Lidocaine block | 5 of 7 characteristics | Se 1.00 (0.75,1.00); Sp 0.66 (0.46, 0.82) |
| | | | | | | 5 of 7 characteristics including relieved by recumbancy | Se 0.92 (0.67, 0.99); Sp 0.79 (0.62, 0.91) |
| Revel | 1992 | NA | N=40 | Patients suffering from LBP whatever the anatomic structure thought to be involved | 75% response on Lidocaine block | 6 of 7 variables | Se 0.45 (0.27, 0.65); Sp 1.00 (0.82, 1.00) |
| | | | | | | 5 of 7 variables | Se 0.64 (0.41, 0.83); Sp 0.89 (0.65, 0.99) |
| | | | | | | 4 of 7 variables | Se 0.82 (0.61, 0.93); Sp 0.78 (0.55, 0.91) |
| **Diagnostic performance of clinical tests for sacroiliac joint pain (Sivayogam, 2011)** | | | | | | | |
| PRIMARY STUDIES | | | | | | | |
| First author | Year of publication | Setting(s) of data collection | Number of participants (N) | Population | Reference standard | Index test | Diagnostic accuracy data |
| Stanford and Burnhan | 2010 | NA | N=34 | Patients with LBP that was refractory to non-invasive conservative spine treatment for >6/12 | Pre-block (SIJB with 1.5 ml lidocaine 2% and bupivacaine 0.25%; 80% index pain relief) | Composites of distraction, thigh trust, Gaenslen's test, compression and sacral thrust (3 or more positive tests) | Se 0.82 (0.58, 0.95); Sp 0.57 (0.45, 0.63); +LR 1.9; -LR 0.31 |

| | | | | | | | |
|---|---|---|---|---|---|---|---|
| | | | | | Post-block (normalization) | | Se 0.89 (0.71, 0.98); Sp 0.57 (0.45, 0.63); +LR 1.3; -LR 0.36 |
| Van der Wurff | 2006 | Tertiray | N=140 | Patients referred for SIJ blocks, with pain principally below L5 | Double SIJB with 2 ml lidocaine 2% or bupivacaine 0.25%; 50% pain relief on VAS | One or more positive tests | Se 1.00 (1.00, 1.00); Sp 42.4 (25.5, 60.8); +LR 1.74; -LR 0.00 |
| | | | | | | Two or more positive tests | Se 0.93 (0.76, 0.99); Sp 0.58 (0.39, 0.75); +LR 2.18; -LR 0.13 |
| | | | | | | Three or more positive tests | Se 0.85 (0.72,0.99); Sp 0.79 (0.65, 0.93); +LR 4.02; -LR 0.19 |
| | | | | | | Four or more positive tests | Se 0.30 (0.11, 0.46); Sp 0.82 (0.64, 0.93); +LR 1.43; -LR 0.91 |
| | | | | | | Five or more positive tests | Se 0.00 (0.00, 0.00); Sp 1.00 (1.00, 1.00), +LR 0.00; -LR 1.00 |
| Laslett | 2005 | Secondary | N=48 | Patients with buttock pain, with or without lumbar or lower extremity symptoms | Double SIJB with 1.5 ml lidocaine and bupivacaine; pain provocation followed by 80% pain relief on NRS | One or more positive tests | Se 1.00 (0.81,1.00); Sp 0.44 (0.28, 0.61); +LR 1.78; -LR 0.00 |
| | | | | | | Two or more positive tests | Se 0.93 (0.72, 0.99); Sp 0.66 (0.48, 0.80); +LR 2.73; -LR 0.10 |
| | | | | | | Three or more positive tests | Se 0.94 (0.72, 0.99); Sp 0.78 (0.61, 0.89); +LR 4.29; -LR 0.80 |
| | | | | | | Four or more positive tests | Se 0.60 (0.36, 0.80); Sp 0.81 (0.65, 0.91); +LR 3.20; -LR 0.49 |
| | | | | | | Five or more positive tests | Se 0.27 (0.11, 0.52); Sp 0.88 (0.72, 0.95); +LR 2.13; -LR 0.84 |
| | | | | | | Two positive tests of distraction, thigh thrust, compression, and sacral thrust | Se 0.88 (0.64, 0.97); Sp 0.78 (0.61, 0.89); +LR 4.00; -LR 0.16 |
| Laslett | 2003 | Tertiary | N=48 | Patients with buttock pain, with or | | Three or more positive tests | Se 0.91 (0.62, 0.98); Sp 0.78 (0.61, 0.89); +LR 4.16; -LR 0.12 |

| | | | | | | | |
|---|---|---|---|---|---|---|---|
| | | | | without lumbar or lower extremity symptoms, referred to a private radiology practice | | Three or more positive tests in the absence of centralization during repeated movement test | Se 0.91 (0.62, 0.98); Sp 0.87 (0.68, 0.96); +LR 6.97; -LR 0.10 |
| **Systematic review of tests to identify the disc, SIJ or facet joint as the source of low back pain (Hancock, 2007)** | | | | | | | |
| PRIMARY STUDIES | | | | | | | |
| First author | Year of publication | Setting(s) of data collection | Number of participants | Population | Reference standard | Index test | Diagnostic accuracy data |
| Van der Wurff | 2006 | Tertiary | N=140 | Patients referred for SIJ blocks, with pain principally below L5 | Double SIJB with 2 ml lidocaine 2% or bupivacaine 0.25%; 50% pain relief on VAS | 3 of more positive pain provocation procedures | Se 84 (65, 95); Sp 78 (60, 90); +LR 3.8 (2.0, 7.3); -LR 0.21 (0.09, 0.49) |
| Laslett | 2005 | Secondary | N=48 | Patients with buttock pain, with or without lumbar or lower extremity symptoms | Double SIJB with 1.5 ml lidocaine and bupivacaine; pain provocation followed by 80% pain relief on NRS | 3 of more positive pain provocation procedures | Se 74 (50, 90); Sp 74 (55, 89); +LR 2.9 (1.5, 5.6); -LR 0.35 (0.17, 0.75) |
| Laslett | 2004 | Secondary | N=151 | Patients with low back pain with or without lower extremity symptoms, referred to a private radiology practice | ZJ blocks | Revel's criteria | Se 18 (5, 43); Sp 93 (86,97); +LR 2.6 (0.8, 8.6); -LR 0.88 (0.70, 1.10) |
| Laslett | 2003 | Tertiary (Private radiology practice) | N=81 | Adult patients with chronic lumbar or lumbopelvic pain who were referred to a private radiology practice | ≥80% pain relief after lumbar zygapophysial joint injection | 3 of more positive pain provocation procedures | Se 89 (59,99); Sp 80 (62, 92); +LR 4.4 (2.1, 8.9); -LR 0.15 (0.03, 0.66) |

| | | | | | | | |
|---|---|---|---|---|---|---|---|
| Young | 2003 | Secondary (Private radiology practice) | N=81 | Adult patients with chronic lumbar or lumbopelvic pain who were referred to a private radiology practice | ≥80% pain relief after lumbar zygapophysial joint injection | 3 of more positive pain provocation procedures | Se 76 (56, 90); Sp 69 (50, 85); +LR 2.5 (1.4, 4.4); -LR 0.35 (0.17, 0.71) |
| Manchikanti | 2000 | Secondary (One private pain management practice, in a non- university setting) | N=200 | Patients presenting to private pain management centre, with chronic LBP +/- lower extremity pain | Double diagnostic block | Revel's criteria | Se 13 (7, 22); Sp 84 (76, 90); +LR 0.8 (0.4, 1.7); -LR 1.03 (0.92, 1.16) |
| Revel | 1998 | Tertiary | N=42 | Patients with LBP >3/12 referred for facet injection | 75% response on Lidocaine block | Revel's criteria | Se 96 (71, 100); Sp 65 (46, 81); +LR 2.8 (1.7, 4.5); -LR 0.06 (0.00,0.85) |
| Revel | 1992 | NA | N=40 | Patients suffering from LBP whatever the anatomic structure thought to be involved | 75% response on Lidocaine block | Revel's criteria | Se 63 (41, 82); Sp 87 (64, 98); +LR 4.8 (1.4, 15.9); -LR 0.43 (0.24, 0.75) |

Table S12: Diagnostic accuracy of demographics for the diagnostic of cauda equina syndrome

| **The diagnostic value of red flags in thoracolumbar pain: a systematic review (Maselli, 2020)** | | | | | | | |
|---|---|---|---|---|---|---|---|
| PRIMARY STUDIES | | | | | | | |
| First author | Year of publication | Setting(s) of data collection | Number of participants (N) | Population | Reference standard | Index test | Diagnostic accuracy data |
| Van Den Bosch | 2004 | Primary | N=2007 | Patients referred with low back pain for lumbar spine radiography | Lumbar radiographs | Age 55 or more | +LR 1.5-8 |

Table S13: Diagnostic accuracy of demographics for the diagnostic of spinal fracture

| **The diagnostic value of red flags in thoracolumbar pain: a systematic review (Maselli, 2020)** | | | | | | | |
|---|---|---|---|---|---|---|---|
| PRIMARY STUDIES | | | | | | | |
| First author | Year of publication | Setting(s) of data collection | Number of participants (N) | Population | Reference standard | Index test | Diagnostic accuracy data |
| Premkumar | 2018 | NA Retrospective study of medical files | N= 9940 | Patients visiting their medical doctor for a chief complaint of LBP | Imaging reports | Age > 50 | Se 0.74; Sp 0.33; +LR 1.1 (1.05-1.16); -LR 0.79 (0.69-0.91) |
| | | | | | | Age > 70 | Se 0.39; Sp 0.80; +LR 1.55 (1.36–1.76); -LR 0.86 (0.82–0.91) |
| Enthoven | 2016 | Primary | N=669 | Patients with back pain consulting their GP | Lumbar radiographs | Age > 75 | Se 0.45 (0.28–0.62); Sp 0.85(0.82–0.88); +LR 3.1 (2.0–4.7); -LR 0.6 (0.5–0.9) |
| Roman | 2010 | Secondary | N=1448 | Patients with lumbar-related disorders | Standard radiograph or CT assessing sagittal alignment, vertebral body compression and | Age>50 | Se 0.95 (0.83–0.95); Sp 0.39 (0.38–0.40); +LR 1.5 (1.3–1.5); -LR 0.14 (0.03–0.45) |
| | | | | | | Body mass index < 22 | Se 0.38 (0.24, 0.55); 0.83 (0.82, 0.84); +LR 2.3 (1.4, 3.4); -LR 0.74 (0.54, 0.91) |

| | | | | | | | |
|---|---|---|---|---|---|---|---|
| | | | | | spinal canal dimensions | Female gender | Se 0.90 (0.76, 0.96); 0.41 (0.41, 0,42); +LR 1.5 (1.3, 1.6); -LR 0.26 (0.10, 0.60) |
| Henschke | 2009 | Primary | N= 1172 | Patients presenting with acute LBP | Imaging studies and specialist view | Age > 70 | Se 0.50; Sp 0.96; +LR 11.0 (4.65, 19.48); -LR 0.52 (0.23,0.82) |
| Van Den Bosch | 2004 | Primary | N=2007 | Patients referred with low back pain for lumbar spine radiography | Lumbar radiographs | Age 55 or more | +LR 1.5-8 |
| **Red flags to screen for vertebral fracture in patients presenting with low-back pain (Williams, 2013)** | | | | | | | |
| PRIMARY STUDIES | | | | | | | |
| First author | Year of publication | Setting(s) of data collection | Number of participants (N) | Population | Reference standard | Index test | Diagnostic accuracy data |
| Roman | 2010 | Secondary | N=1448 | Patients with lumbar-related disorders | Standard radiograph or CT assessing sagittal alignment, vertebral body compression and spinal canal dimensions | Age > 52 years | Se 0.95 (0.82, 0.99); Sp 0.39 (0.36, 0.41); LR + 1.52 (1.42, 1.68); LR - 0.14 (0.04, 0.53) |
| | | | | | | Gender | Se 0.89 (0.75, 0.97); Sp 0.41 (0.38, 0.44); LR + 1.51 (1.35, 1.71); LR - 0.26 (0.10, 0.65) |
| | | | | | | BMI <23 | Se 0.37 (0.22, 0.54); Sp 0.83 (0.81, 0.85) ; LR +2.22 (1.44 - 3.42); LR- 0.76 (0.59, 0.97) |
| Henschke | 2009 | Primary | N= 1172 | Patients presenting with acute LBP | Imaging studies and specialist view | Age > 50 years | Se 0.63 (0.24, 0.91); Sp 0.66 (0.63, 0.69); LR + 1.84 (1.07-3.17); LR - 0.57 (0.23-1.39) |
| | | | | | | Age > 54 years | Se 0.63 (0.24, 0.91); Sp 0.88 (0.86, 0.90); LR+ 2.57 (1.49, 4.44); LR- 0.50 (0.2, 1.21) |
| | | | | | | Age > 64 years | Se 0.63 (0.24, 0.91); Sp 0.96 (0.94, 0.97); LR+ 7.13 (4.04,12.59); LR- 0.41 (0.17, 1.01) |

| | | | | | | | |
|---|---|---|---|---|---|---|---|
| | | | | | | Age > 70 years | Se 0.50 (0.16, 0.84); Sp 0.96 (0.94, 0.97); LR+ 11.19 (5.33, 23.51); LR- 0.52 (0.26, 1.05) |
| | | | | | | Age > 74 years | Se 0.25 (0.03, 0.65); Sp 0.97 (0.96, 0.98); LR + 9.39 (2.69, 32.75); LR- 0.77 (0.52, 1.15) |
| | | | | | | Female Age > 54 years | Se 0.63 (0.24, 0.91); Sp 0.88 (0.86, 0.90); LR+ 5.39 (3.08, 9.43); LR - 0.42 (0.17,1.04) |
| | | | | | | Female Age > 64 years | Se 0.63 (0.24, 0.91); Sp 0.96 (0.94, 0.97); LR + 14.59 (8.00, 26.61); LR - 0.39 (0.16, 0.96) |
| | | | | | | Female Age > 74 years | Se 0.25 (0.03, 0.65); Sp 0.98 (0.98, 0.99); LR+ 16.17 (4.47, 58.43); LR- 0.76 (0.51, 1.14) |
| | | | | | | Age > 50 years | Se 0.63 (0.24, 0.91); Sp 0.66 (0.63, 0.69); LR + 1.84 (1.07-3.17); LR - 0.57 (0.23-1.39) |
| Van Den Bosch | 2004 | Primary | N=2007 | Patients referred with low back pain for lumbar spine radiography | Lumbar radiographs | Age > 54 years | Se 0.83 (0.73, 0.90); Sp 0.52 (0.49, 0.54); LR+ 1.72 (1.54, 1.91); LR-0.33 (0.20, 0.53) |
| | | | | | | Age > 64 years | Se 0.78 (0.68, 0.87); Sp 0.68 (0.66, 0.70) LR + 2.46 (2.16, 2.8); LR- 0.32 (0.21, 0.48) |
| | | | | | | Age > 74 years | Se 0.59 (0.48, 0.70); Sp 0.84 (0.82, 0.86); LR + 3.69 (3.00, 4.53); LR- 0.49 (0.38, 0.63) |
| | | | | | | Female Age > 54 years | Se 0.63 (0.51, 0.73); Sp 0.69 (0.67, 0.71); LR +2.01 (1.68, 2.40); LR - 0.54 (0.41, 0.72) |
| | | | | | | Female Age > 64 years | Se 0.59 (0.48, 0.70); Sp 0.59 (0.48, 0.70); LR + 2.75 (2.26, 3.35); LR - 0.52 (0.40, 0.68) |

| | | | | | | | |
|---|---|---|---|---|---|---|---|
| | | | | | | Female Age > 74 years | LR + 4.14 (3.17, 5.44); LR - 0.62 (0.51 - 0.75) |
| | | | | | | Gender | Se 0.72 (0.61, 0.82); Sp 0.43 (0.41, 0.45) |
| Deyo | 1986 | Primary | N=621 | Patients presenting with LBP, 311 received imaging | X-ray - anteroposterior and lateral lumbar view | Age > 50 years | Se 0.79 (0.49, 0.95); Sp 0.64 (0.58, 0.69); LR+ 2.16 (1.58-2.95); LR- 0.34 (0.12-0.92) |

Table S14: Diagnostic accuracy of demographics for the diagnosis of spinal malignancy

| **The diagnostic value of red flags in thoracolumbar pain: a systematic review (Maselli, 2020)** | | | | | | | |
|---|---|---|---|---|---|---|---|
| PRIMARY STUDIES | | | | | | | |
| First author | Year of publication | Setting(s) of data collection | Number of participants (N) | Population | Reference standard | Index test | Diagnostic accuracy data |
| Premkumar | 2018 | NA Retrospective study of medical files | N= 9940 | Patients visiting their medical doctor for a chief complaint of LBP | Imaging reports | Age > 50 | Se 0.72; Sp 0.33; LR+ 1.06 (0.96, 1.17); LR- 0.87 (0.68, 1.11) |
| | | | | | | Age > 70 | Se 0.22; Sp 0.80; LR+ 1.1 (0.82, 1.47); 0.97 (0.9, 1.06) |
| **Red flags to screen for malignancy in patients with low-back pain (Henschke, 2013)** | | | | | | | |
| PRIMARY STUDIES | | | | | | | |
| First author | Year of publication | Setting(s) of data collection | Number of participants (N) | Population | Reference standard | Index test | Diagnostic accuracy data |
| Henschke | 2009 | Primary | N= 1172 | Patients presenting with acute LBP | Imaging studies and specialist view | Age > 50 | Sp 0.66 (0.63; 0.69) |
| | | | | | | Age > 70 | Sp 0.95 (0.94; 0.96) |
| Jacobson | 1997 | Secondary | N=491 | Patients with a complaint of middle to lower back pain | CT Scan | Age > 50 | Se 1 (0.81,1); Sp 0.41 (0.35, 0.48) |

| | | | | | | | |
|---|---|---|---|---|---|---|---|
| Frazier | 1989 | Primary | N=1037 | Patients with acute lumbosacral back pain | Diagnostic imaging and follow-up | Age >50 | Se 0.50 (0.01, 0.99); Sp 0.74 (0.70, 0.78) |
| Deyo | 1988 | Primary | N=1975 | Patients with a primary complaint of LBP of less than 1-month duration | Lumbar radiographs | Age > 50 | Se 0.77 (0.46, 0.95); Sp 0.71 (0.69, 0.73) |
| Deyo | 1986 | Primary | N=621 | Patients presenting with LBP, 311 received imaging | X-ray - anteroposterior and lateral lumbar view | Age > 50 | Se 0.75(0.19, 0.99); Sp 0.70 (0.66, 0.74) |

Table S15: Diagnostic accuracy of demographics for the diagnosis any serious spinal pathology

| **The diagnostic value of red flags in thoracolumbar pain: a systematic review (Maselli, 2020)** | | | | | | | |
|---|---|---|---|---|---|---|---|
| PRIMARY STUDIES | | | | | | | |
| First author | Year of publication | Setting(s) of data collection | Number of participants (N) | Population | Reference standard | Index test | Diagnostic accuracy data |
| Shaw | 2020 | Primary (ED) | N= 1000 | Patients presenting to the ED with back pain | NA | Age over 70 years | Se 48.5; Sp 79.9 ; LR+ 1.9 (1.3, 2.8); LR- 0.7 (0.5–1.0) |

Table S16: Diagnostic accuracy of patient history findings for the diagnosis of CES

| **The diagnostic value of red flags in thoracolumbar pain: a systematic review (Maselli, 2020)** | | | | | | | |
|---|---|---|---|---|---|---|---|
| PRIMARY STUDIES | | | | | | | |
| First author | Year of publication | Setting(s) of data collection | Number of participants (N) | Population | Reference standard | Index test | Diagnostic accuracy data |
| Tsiang | 2019 | Tertiary | N=500 | Patients with LBP seen at a large tertiary care spine clinic | NA | Bladder dysfunction | Se 1.00; Sp 0.77 |
| | | | | | | Bladder dysfunction patient reported | Se 0.50; Sp 0.87 |
| | | | | | | Bowel dysfunction | Se 0.50; Sp 0.87 |
| | | | | | | Lower limbs weakness | Se 1.00; 0.77 |
| Premkumar | 2018 | NA Retrospective study of medical files | N= 9940 | Patients visiting their medical doctor for a chief complaint of LBP | Imaging reports | Bladder dysfunction | Se 0.22; Sp 0.90; LR+ 2.31 (1.25, 4.27); LR- 0.86 (0.72, 1.03) |
| | | | | | | Bowel dysfunction | Se 0.14; Sp 0.95; LR+ 2.78 (1.23, 6.3); LR- 0.91 (0.8, 1.03) |
| Raison | 2014 | Primary (ED) | N=206 | Patients with LBP attending the ED | MRI | Bladder dysfunction | Se 0.65 (0.44, 0.82); Sp 0.73 (0.66, 0.80); LR+ 2.45 |
| | | | | | | Bowel dysfunction | Se 0.65 (0.44, 0.82); Sp 0.73 (0.66, 0.80); LR+ 2.45 |
| | | | | | | Saddle dysfunction | Se 0.27 (0.12, 0.48); Sp 0.87 (0.81, 0.92); LR+ 2.11 |
| **What is the diagnostic accuracy of red flags related to cauda equina syndrome (CES), when compared to Magnetic Resonance Imaging (MRI)? A systematic review (Dionne, 2019)** | | | | | | | |
| POOLED DATA | | | | | | | |
| First author | Year of publication | Setting(s) of data collection | Number of participants (N) / Number of studies (n) | Population | Reference standard | Index test | Diagnostic accuracy data (pooled estimates) |

| | | | | | | | |
|---|---|---|---|---|---|---|---|
| Dionne | 2019 | Mixed (Secondary and tertiary care settings) | N=869 | Adults who presented with a suspected CES from an insidious onset or herniated disc prolapse | MRI | Bowel incontinence (n=4 studies) | Se 0.18 (0.09, 0.32); Sp 0.86 (0.80, 0.91); LR+ 1.60 (0.65, 3.94); LR- 0.96 (0.78, 1.18) |
| | | | | | | Leg pain (n=5 studies) | Se 0.46 (0.32, 0.59); Sp 0.65 (0.58, 0.72); LR+ 1.45 (0.81, 2.58); LR- 0.90 (0.60, 1.35) |
| | | | n=7 | | | Urinary retention (n=5 studies) | Se 0.36 (0.25, 0.49); Sp 0.61 (0.54, 0.68); LR+ 0.99 (0.61, 1.61); LR- 1.06 (0.86) |
| | | | | | | Urinary incontinence (n=5 studies) | Se 0.36 (0.25, 0.48); Sp 0.56 (0.49, 0.64); LR+ 0.80 (0.56, 1.14); LR- 1.13 (0.90, 1.43) |
| | | | | | | Back pain (n=3 studies) | Se 0.92 (0.92, 0.98); Sp 0.30 (0.23, 0.37); LR+ 1,23 (0.77, 2.20); LR- 0.40 (0.05, 2.41) |

Table S17: Diagnostic accuracy of patient history findings for the diagnosis of spinal fracture

| **The diagnostic value of red flags in thoracolumbar pain: a systematic review (Maselli, 2020)** | | | | | | | |
|---|---|---|---|---|---|---|---|
| PRIMARY STUDIES | | | | | | | |
| First author | Year of publication | Setting(s) of data collection | Number of participants (N) | Population | Reference standard | Index test | Diagnostic accuracy data |
| Tsiang | 2019 | Tertiary | N=500 | Patients with LBP seen at a large tertiary care spine clinic | NA | History of trauma | Se 0.81; Sp 0.79 |
| | | | | | | Osteoporosis | Se 0.81; Sp 0.79 |
| Premkumar | 2018 | NA Retrospective study of medical files | N= 9940 | Patients visiting their medical doctor for a chief complaint of LBP | Imaging reports | History of trauma | Se 24.7; Sp 88.6; LR+ 2.17 (1.86–2.54); LR- 0.84 (0.81, 0.89) |
| Enthoven | 2016 | Primary | N=669 | Patients with back pain consulting their GP | Lumbar radiographs | Corticosteroids use | Se 0.18 (0.05, 0.31); Sp 0.93 (0.91, 0.95); LR+ 2.5 (1.1, 5.3); LR- 0.90 (0.8, 1.0) |
| | | | | | | History of trauma | Se 0.21 (0.07, 0.35); Sp 0.97 (0.95, 0.98); +LR 6.2 (2.8, 13.5); LR- 0.80 (0.5,1.3) |
| | | | | | | Severe disability | Se 0.30 (0.14, 0.46); 0.87 (0.84, 0.90); LR + 2.3 (1.3, 4.2); LR- 0.8 (0.6, 1.0) |
| | | | | | | Sudden decrease in height | 0.09 (0.01, 0.19); Sp 0.97 (0.95, 0.98); LR+ 2.9 (0.9, 9.4); LR- 0.90 (0.8, 1.0) |
| Roman | 2010 | Secondary | N=1448 | Patients with lumbar-related disorders | Standard radiograph or CT assessing sagittal | No regular exercise | Se 0.81 (0.65, 0.91); Sp 0.44 (0.43, 0.45); LR+ 1.5 (1.2, 1.6); LR- 0.43 (0.20, 0.80) |

| | | | | | | | |
|---|---|---|---|---|---|---|---|
| | | | | | alignment, vertebral body compression and spinal canal dimensions | Sitting decrease pain | Se 0.29 (0.27, 0.32); 0.81 (0.79, 0.83); LR+ 1.6 (1.2, 1.9); LR- 0.87 (0.82, 0.92) |
| Henschke | 2009 | Primary | N= 1172 | Patients presenting with acute LBP | Imaging studies and specialist view | Corticosteroids use | Se 0.25; Sp 1.00; LR+ 48.5 (11.62, 165.22); LR- 0.75 (0.41, 0.93) |
| | | | | | | History of trauma | Se 0.25; Sp 0.98; LR+ 10.0 (2.76, 26.36); LR- 0.77 (0.42, 0.95) |
| **Red flags to screen for vertebral fracture in patients presenting with low-back pain (Williams, 2013)** | | | | | | | |
| PRIMARY STUDIES | | | | | | | |
| First author | Year of publication | Setting(s) of data collection | Number of participants (N) | Population | Reference standard | Index test | Diagnostic accuracy data |
| Roman | 2010 | Secondary | N=1448 | Patients with lumbar-related disorders | Standard radiograph or CT assessing sagittal alignment, vertebral body compression and spinal canal dimensions | Osteoarthritis | Se 0.50 (0.33, 0.67); Sp 0.52 (0.50, 0.55); LR + 1.05 (0.76, 1.45); LR - 0.95 (0.69, 1.32) |
| | | | | | | No regular exercise | Se 0.82 (0.66, 0.92); Sp 0.44 (0.42, 0.47); LR + 1.47 (1.25, 1.75); LR - 0.42 (0.21, 0.81) |
| | | | | | | Absence of buttock / leg pain | Se 0.32 (0.18, 0.49); Sp 0.86 (0.84, 0.88); LR+ 2.24 (1.38, 3.64); LR- 0.80 (0.64, 0.99) |
| | | | | | | Decreased pain on sitting | Se 0.29 (0.15, 0.46); Sp 0.23 (0.21, 0.25); LR + 1.56 (0.94, 2.59); LR - 0.87 (0.71, 1.07) |
| Reinus | 1998 | Tertiary | N=482 | Patients with back pain presenting to the ED department | Lumbosacral radiographs | History of trauma | Se 0.07 (0.02, 0.18); Sp 0.60 (0.56, 0.65); LR+ 0.18 (0.07, 0.48); LR - 1.54 (1.38, 1.71) |
| Gibson | 1992 | Tertiary | N=225 | Patients presenting to the ED department with lumbar pain of less than 48 hours | Plain radiographs | History of trauma | Se 1.00 (0.59, 1.00); Sp 0.51 (0.41, 0.62); LR+ 1.93 (1.48, 2.52); LR - 0.121 (0.01, 1.79) |

| | | | | | | | |
|---|---|---|---|---|---|---|---|
| Deyo | 1986 | Primary | N=621 | Patients presenting with LBP, 311 received imaging | X-ray - anteroposterior and lateral lumbar view | Trauma | Se 0.36 (0.13, 0.65); Sp 0.90 (0.86, 0.93); LR+ 3.97 (0.20, 79.15); LR- 0.98 (0.89, 1.07) |
| | | | | | | Corticosteroids use | Se 0.0 (0.0, 0.23); Sp 0.99 (0.98, 1.00); LR + 3.97 (0.20, 79.15); LR- 0.98 (0.89, 1.07) |
| Patrick | 1983 | Tertiary | N=552 | Patients complaining of LBP presenting to the ED department | Lumbar radiographs | History of trauma | Se 0.80 (0.64, 0.91); Sp 0.55 (0.50, 0.59); LR+ 1.77 (1.48, 2.13); LR - 0.36 (0.20, 0.68) |
| Scavone | 1981 | Primary | N=871 | Patients with LBP presenting to the university teaching hospital medical centre | Lumbar radiographs | History of trauma | Se 0.65 (0.44, 0.83); Sp 0.95 (0.93, 0.96); LR+ 12.85 (8.58, 19.24); LR- 0.36 (0.22, 0.62) |
| | | | | | | Hip/leg pain | LR + 0.21 (0.01, 3.35); LR -1.08 (1.02, 1.14) |
| | | | | | | Sciatica | Se 0.04 (0.00, 0.20); Sp 0.91 (0.89, 0.93) |

**A systematic review identifies five "red flags" to screen for vertebral fracture in patients with low back pain (Henschke, 2008)**

PRIMARY STUDIES

| First author | Year of publication | Setting(s) of data collection | Number of participants (N) | Population | Reference standard | Index test | Diagnostic accuracy data |
|---|---|---|---|---|---|---|---|
| Holmes | 2003 | Secondary | N=2404 | Patients with back pain presenting to the ED department | Lumbar radiographs | Severe mechanism of injury | Se 0.61; Sp 0.64; LR+ 1.7 (1.4, 1.9); LR- 0.62 (0.50, 0.75) |
| Reinus | 1998 | Secondary | N=482 | Patients with back pain presenting to the ED department | Lumbosacral radiographs | Acute trauma | Se 0.40; Sp 0.64; LR+ 1.1 (0.5, 2.0); LR- 0.94 (0.49, 1.31) |

Table S18: Diagnostic accuracy of patient history findings for the diagnosis of spinal malignancy

| **The diagnostic value of red flags in thoracolumbar pain: a systematic review (Maselli, 2020)** | | | | | | | |
|---|---|---|---|---|---|---|---|
| PRIMARY STUDIES | | | | | | | |
| First author | Year of publication | Setting(s) of data collection | Number of participants (N) | Population | Reference standard | Index test | Diagnostic accuracy data |
| Tsiang | 2019 | Tertiary | N=500 | Patients with LBP seen at a large tertiary care spine clinic | NA | History of malignancy | Se 91.7; Sp 77.8 |
| Premkumar | 2018 | NA Retrospective study of medical files | N= 9940 | Patients visiting their medical doctor for a chief complaint of LBP | Imaging reports | Night pain | Se 0.55; Sp 0.42; LR+ 0.85 (0.83,1.1); LR- 1.07 (0.9, 1.27) |
| | | | | | | Unexplained weight loss | Se 0.08; Sp 0.96; LR+ 1.87 (1.1, 3.17); LR- 0.96 (0.92, 1.01) |
| | | | | | | History of malignancy | Se 0.32; Sp 0.96; +LR 7.25 (5.65, 9.3); -LR 0.71 (0.64, 0.79) |
| Cook | 2011 | Tertiary | N=1109 | Patients with LBP seen at a spine surgery center | Imaging | No pain during flexion | Se 0.67 (0.55, 0.77); Sp 0.41 (0.40, 0.42); LR+ 1.1 (0.9, 1.3); LR- 0.80 (0.56, 1.1) |
| | | | | | | No pain during extension | Se 0.65 (0.54, 0.75), Sp 0.50 (0.49, 0.51); LR+ 1.3 (1.1, 1.5); LR- 0.70 (0.49, 0.94) |
| | | | | | | No pain during right side flexion | Se 0.96 (0.88, 0.98); Sp 0.04 (0.04, 0.05); LR+ 0.94 (0.85,1); LR- 1 (0.92, 1.01) |
| | | | | | | No pain during left side flexion | Se 0.96 (0.88, 0.98); 0.04 (0.03, 0.04); LR + 0.99 (0.91, 1); LR-1.1 (0.37, 3.2) |
| Donner-Banzhoff | 2006 | Primary | N=1378 | Patients with LBP presenting to a GP | Experts opinions | Clinical judgment | Se 0.50; Sp 0.83; LR+ 2.95; LR- 0.6 |
| **Red flags to screen for malignancy in patients with low-back pain (Henschke, 2013)** | | | | | | | |
| PRIMARY STUDIES | | | | | | | |

| First author | Year of publication | Setting(s) of data collection | Number of participants (N) | Population | Reference standard | Index test | Diagnostic accuracy data |
|---|---|---|---|---|---|---|---|
| Henschke | 2009 | Primary | N= 1172 | Patients presenting with acute LBP | Imaging studies and specialist view | Constant Progressive Pain | Se 0 (0.0, 0.0); Sp 0.97 (0.96, 0.96) |
| | | | | | | Gradual Onset before age 40 | Se 0 (0.0, 0.0); Sp 0.91 (0.90, 0.93) |
| | | | | | | Insidious onset | Se 0 (0.0, 0.0); Sp 0.83 (0.80, 0.85) |
| | | | | | | Previous history of cancer | Se 0 (0.0, 0.0); Sp 0.96 (0.95, 0.97) |
| | | | | | | Systematically unwell | Se 0 (0.0, 0.0); Sp 0.96 (0.96, 0.97) |
| | | | | | | Tried bedrest with no relief | Se 0 (0.0, 0.0); Sp 0.84 (0.81, 0.86) |
| | | | | | | Unexplained weight loss | Se 0 (0.0, 0.0); Sp 1 (0.99, 0.1) |
| Donner-Banzhoff | 2006 | Primary | N=1378 | Patients with LBP presenting to a GP | Experts opinions | Is the LBP familiar | Se 0 (0.0, 0.97); Sp 0.83 (0.81, 0.85) |
| Reinus | 1998 | Tertiary | N=482 | Patients with back pain presenting to the ED department | Lumbosacral radiographs | Previous history of cancer | Se 1.0 (0.59, 1); Sp 0.97 (0.95, 0.96) |
| Deyo | 1988 | Primary | N=1975 | Patients with a primary complaint of LBP of less than 1-month duration | Lumbar radiographs | Duration of this episode >1month | Se 0.50 (0.21, 0.79); Sp 0.81 (0.79, 0.83) |
| | | | | | | Insidious onset | Se 0.62 (0.32, 0.86); Sp 0.42 (0.40, 0.44) |
| | | | | | | Not improved after 1 month | Se 0.31 (0.09, 0.51); Sp 0.90 (0.89, 0.91) |
| | | | | | | Previous history of cancer | Se 0.31(0.09, 0.61); Sp 0.96 (0.97, 0.99) |
| | | | | | | Recent back injury | Se 0.0 (0.0, 0.25); Sp 0.82 (0.80, 0.84) |
| | | | | | | Severe pain | Se 0.23 (0.05, 0.54); Sp 0.85 (0.83, 0.87) |
| | | | | | | Thoracic pain | Se 0.17 (0.02, 0.48); Sp 0.84 (0.82,0.86) |

| | | | | | | | |
|---|---|---|---|---|---|---|---|
| | | | | | | Tried bedrest with no relief | Se 1 (0.4, 1); Sp 0.46 (0.43, 0.49) |
| | | | | | | Unexplained weight loss | Se 0.15 (0.02, 0.45); Sp 0.94 (0.93, 0.95) |
| Deyo | 1986 | Primary | N=621 | Patients presenting with LBP, 311 received imaging | X-ray - anteroposterior and lateral lumbar view | Not improved after 1 month | Se 0.25 (0.01,0.81); Sp 0.90 (0.88,0.93) |

Table S19: Diagnostic accuracy of patient history findings for the diagnosis of spinal infection

| **The diagnostic value of red flags in thoracolumbar pain: a systematic review (Maselli, 2020)** | | | | | | | |
|---|---|---|---|---|---|---|---|
| PRIMARY STUDIES | | | | | | | |
| First author | Year of publication | Setting(s) of data collection | Number of participants (N) | Population | Reference standard | Index test | Diagnostic accuracy data |
| Premkumar | 2018 | NA Retrospective study of medical files | N= 9940 | Patients visiting their medical doctor for a chief complaint of LBP | Imaging reports | Chills | Se 0.12; Sp 0.93; LR+ 1.71 (1.04, 2.81); LR- 0.95 (0.89, 1.01) |
| | | | | | | Night pain | Se 0.58; Sp 0.42; LR+ 0.99; LR- 1.02 |
| | | | | | | Sweating | Se 0.12; Sp 0.93; LR+ 1.71 (1.04, 2.81); LR- 0.95 (0.89, 1.01) |
| | | | | | | Persistent night sweating | Se 0.18; Sp 0.86, +LR 1.26 (0.85, 1.86); LR- 0.96 (0.88, 1.04) |
| | | | | | | Recent infection | Se 0.24; Sp 0.97; LR+ 9.31 (6.63, 13.07); LR- 0.78 (0.7, 0.86) |
| **Low Back Pain in the Emergency Department: Prevalence of Serious Spinal Pathologies and Diagnostic Accuracy of Red Flags (Galliker, 2020)** | | | | | | | |
| PRIMARY STUDIES | | | | | | | |
| First author | Year of publication | Setting(s) of data collection | Number of participants (N) | Population | Reference standard | Index test | Diagnostic accuracy data |

| | | | | | | | |
|---|---|---|---|---|---|---|---|
| Davis | 2011 | Primary (ED) | N=55 | Patients who present to ED with spine pain | Imaging | Intravenous drug abuse | LR+ 13.7 (11.4, 16.5); LR- 0.4 (0.3, 0.5) |
| | | | | | | Recent spine fracture | LR+ 0.5 (5.0, 17.8); LR- 0.9 (0.8, 1.0) |
| | | | | | | Immunocompromised | LR+ 5.1 (3.2, 8.0); LR- 0.9 (0.8, 0.9) |
| | | | | | | Indwelling vascular catheter | LR+ 15.7 (7.9, 31.0); LR- 0.9 (0.9, 1.0) |
| | | | | | | Other infection site | LR+ 13.7 (9.4, 19.8); LR- 0.8 (0.7, 0.9) |

Table S20: Diagnostic accuracy of patient history findings for the diagnosis of serious spinal pathology

| **The diagnostic value of red flags in thoracolumbar pain: a systematic review (Maselli, 2020)** | | | | | | | |
|---|---|---|---|---|---|---|---|
| PRIMARY STUDIES | | | | | | | |
| First author | Year of publication | Setting(s) of data collection | Number of participants (N) | Population | Reference standard | Index test | Diagnostic accuracy data |
| Shaw | 2020 | Primary (ED) | N= 1000 | Patients presenting to the ED with back pain | NA | Acute urinary retention or overflow incontinence | Se 15.2; Sp 97.6; LR+ 6.4 (2.6, 15.7); LR- 0.9 (0.8, 0.1) |
| | | | | | | Anticoagulated | Se 9.1; Sp 95.4; LR+ 2.0 (0.6, 6.0); LR- 1.0 (0.9, 1.1) |
| | | | | | | Constant progressive non-mechanical pain | Se 3; Sp 99.3; LR+ 4.2 (0.5, 33.1); LR- 1.0 (0.9, 1) |
| | | | | | | Flank pain | Se 9.1; Sp 87.1; LR+ 0.7 (0.2, 2.1) LR- 1.0 (0.9, 1.2) |
| | | | | | | Gradual onset before age 40 | Se 0; Sp 99.6; LR+ 0.0; LR- 1.0 |
| | | | | | | Herpes zoster rash | Se 1.1 ; Sp 100; LR- 1.0 (1.0, 1.0) |

| | | | | | | | |
|---|---|---|---|---|---|---|---|
| | | | | | | History of cancer | Se 15.2; Sp 92.1; LR+ 1.9 (0.8, 4.5); LR- 0.9 (0.8, 1.1) |
| | | | | | | History of trauma (major in young/minor in elderly) | Se 27.3; Sp 90.7; LR+ 2.9 (1.6, 5.3); LR- 0.8 (0.7, 1) |
| | | | | | | Immune suppression | Se 12.1; Sp 96.0; LR+ 3.0 (1.1, 7.9); LR- 0.9 (0.8, 1) |
| | | | | | | Inflammatory arthritis or osteoporosis fracture | Se 30.3; Sp 93.4; LR+ 4.6 (2.6, 8.1); LR- 0.8 (0.6, 0.9) |
| | | | | | | Insidious onset | Se 6.1; Sp 97.2; LR+ 2.2 (0.5, 8.8) LR- 1.0 (0.9–1.1) |
| | | | | | | Intravenous drug use | Se 12.1; Sp 98.2; LR+ 6.9 (2.5, 19.4); LR- 0.9 (0.8, 1.0) |
| | | | | | | Iritis, skin rashes (psoriasis), colitis, urethral discharge | Se 1.7; Sp 99.6; LR + 4.6 (0.9, 22.6); LR- 1.0 (1.0, 1.0) |
| | | | | | | Known nephrolithiasis or abdominal aortic aneurysm | Se 6.1; Sp 95.6; LR+ 1.4 (0.3, 5.4) LR- 1.0 (0.9, 1.1) |
| | | | | | | Known spinal Paget's disease | Se 0; Sp 99.9; LR+ 0; LR- 1.0 |
| | | | | | | Morning back stiffness 30 minutes | Se 0; Sp 99.8; LR+ 0; LR- 1.0 |
| | | | | | | Pregnancy | Se 0.6; Sp 99.8; LR+ 2.3 (0.2, 25.2); LR- 1.0 (1.0, 1.0) |
| | | | | | | Progressive motor weakness in legs or gait disturbances | Se 3.0; Sp 98.4; LR+ 1.8 (0.3, 13.4); LR- 1.0 (0.9, 1.1) |
| | | | | | | Prolonged use of corticosteroids | Se 12.1; Sp 96.9; LR+ 3.9 (1.5, 10.5); LR- 0.9 (0.8, 1) |
| | | | | | | Recent infection | Se 18.2; Sp 96.0; LR + 4.5 (2.1, 9.9); LR- 0.9 (0.7, 1) |

| | | | | | | | |
|---|---|---|---|---|---|---|---|
| | | | | | | Recent spinal procedure | Se 3.0; Sp 97.3; LR+ 1.1 (0.2, 8.1) LR- 1.0 (0.9, 1.1) |
| | | | | | | Systemically unwell | Se 62.0; Sp 84.2; LR+ 2.8 (0.7, 11.4); LR- 1.0 (1.0, 1.0) |
| | | | | | | TB history | Se 3.0; Sp 99.7; LR+ 9.8 (1.0, 91.4); LR- 1.0 (0.9–1.0) |
| | | | | | | Thoracic/chest/abdominal pain | Se 3.0; Sp 94.1; LR+ 0.5 (0.1, 3.6); LR- 1.0 (1.0, 1.1) |
| | | | | | | Tried bed rest but no relief | Se 0; Sp 99.8; LR+ 0.0; LR-1.0 |
| | | | | | | Unexplained weight loss | Se 1.1; Sp 99.9; LR+ 9.2 (0.8, 100.6); LR- 1.0 (1.0, 1.0) |
| | | | | | | Urinary symptoms | Se 27.3; Sp 88.7; LR + 2.4 (1.4, 4.3); LR- 0.8 (0.7, 1) |
| | | | | | | Writhing in pain | Se 5.0; Sp 99.3; LR+ 6.9 (2.5, 19.9); LR-1.0 (0.9, 1.0) |
| **Low Back Pain in the Emergency Department: Prevalence of Serious Spinal Pathologies and Diagnostic Accuracy of Red Flags (Galliker, 2020)** | | | | | | | |
| PRIMARY STUDIES | | | | | | | |
| First author | Year of publication | Setting(s) of data collection | Number of participants (N) | Population | Reference standard | Index test | Diagnostic accuracy data |
| Thiruganasambandamoorthy | 2014 | Primary (ED) | N=329 | Patients who present to an academic ED with nontraumatic LBP | Blood work, imaging or both | Current anticoagulants use | LR + 8.7 (3.1, 24.4); LR – 0.8 (0.6, 1.0) |
| | | | | | | New urinary retention | LR+ 7.0 (1.9, 26.0); LR- 0.9 (0.7, 1.0) |

Table S21: Diagnostic accuracy of physical examination findings for the diagnosis of CES
t

| **Can digital rectal examination be used to detect cauda equina compression in people presenting with acute cauda equina syndrome? A systematic review and meta-analysis of diagnostic test accuracy studies (Tabrah, 2022)** | | | | | | | |
|---|---|---|---|---|---|---|---|
| POOLED DATA | | | | | | | |
| First author | Year of publication | Setting(s) of data collection | Number of participants (N)<br>Number of studies (n) | Population | Reference standard | Index test | Diagnostic accuracy data (pooled estimates) |
| Tabrah | 2022 | Secondary and tertiary care settings | N=600<br>n=5 | Adults presenting to secondary or tertiary care with acute CES | MRI | Digital rectal examination (anal tone) | LR+ 1.32 (0.94, 1.66); LR- 1.09 (0.94, 1.26); DOR 1.48 (0.87, 2.51) |
| PRIMARY STUDIES | | | | | | | |
| First author | Year of publication | Setting(s) of data collection | Number of participants (N) | Population | Reference standard | Index test | Diagnostic accuracy data |
| Venkatesan | 2019 | Tertiary | N=92 | Adults presenting to tertiary care with acute CES | MRI | Anal squeeze | Se 0.29; Sp 0.96 |
| Gooding | 2013 | Secondary | N=57 | Adults presenting to the radiology department with suspected CES | MRI | Digital rectal examination (internal sensation) | Se 0.4 (0.1, 0.7); Sp (0.35, 0.68) |
| Domen | 2009 | Secondary | N=58 | Adults presenting to tertiary care with acute CES | MRI | Anal reflexes | Se 0.38; Sp 0.60 (0.2, 4.2) |
| **The diagnostic value of red flags in thoracolumbar pain: a systematic review (Maselli, 2020)** | | | | | | | |
| PRIMARY STUDIES | | | | | | | |
| First author | Year of publication | Setting(s) of data collection | Number of participants (N) | Population | Reference standard | Index test | Diagnostic accuracy data |
| Raison | 2014 | Primary (ED) | N=206 | Patients with LBP attending the ED | MRI | Saddle dysfunction | Se 0.27 (0.12, 0.48); Sp 0.87 (0.81, 0.92); LR+ 2.11 |

| What is the diagnostic accuracy of red flags related to cauda equina syndrome (CES), when compared to Magnetic Resonance Imaging (MRI)? A systematic review (Dionne, 2019) | | | | | | | |
|---|---|---|---|---|---|---|---|
| POOLED DATA | | | | | | | |
| First author | Year of publication | Setting(s) of data collection | Number of participants (N) / Number of studies (n) | Population | Reference standard | Index test | Diagnostic accuracy data (pooled estimates) |
| Dionne | 2019 | Mixed (Secondary and tertiary care settings) | N=869 | Adults who presented with a suspected CES from an insidious onset or herniated disc prolapse | MRI | Saddle anesthesia (n=4 studies) | Se 0.43 (0.31, 0.55); Sp 0.79 (0.74, 0.83); LR+ 1.73 (0.98, 3.08); LR- 0.80 (0.61, 1.05) |
| | | | n=7 | | | Reduced anal tone (n=3 studies) | Se 0.29 (0.15, 0.47); Sp 0.83 (0.76, 0.88); LR+ 1.72 (0.91, 3.24); LR- 0.90 (0.86, 1.07) |

Table S22: Diagnosis accuracy of physical examination findings for the diagnosis of spinal fracture

| The diagnostic value of red flags in thoracolumbar pain: a systematic review (Maselli, 2020) | | | | | | | |
|---|---|---|---|---|---|---|---|
| PRIMARY STUDIES | | | | | | | |
| First author | Year of publication | Setting(s) of data collection | Number of participants (N) | Population | Reference standard | Index test | Diagnostic accuracy data |
| Enthoven | 2016 | Primary | N=669 | Patients with back pain consulting their GP | Lumbar radiographs | Percussion tenderness of spine | Se 0.21(0.07, 0.35); Sp 0.81 (0.78, 0.84); +LR 1.1 (0.6, 2.2); LR- 1.0 (0.8, 1.2) |
| Roman | 2010 | Secondary | N=1448 | Patients with lumbar-related disorders | Standard radiograph or CT assessing sagittal alignment, vertebral body compression and spinal canal dimensions | Gait abnormality | Se 0.66 (0.50; 0.79); Sp 0.23 (0.22, 0.23); LR+ 0.86 (0.65, 1.02); LR- 1.5 (0.91, 2.2) |

| | | | | | | | |
|---|---|---|---|---|---|---|---|
| Hsu | 2003 | NA Retrospective chart review | N=200 | Patients with confirmed thoraco-lumbar fracture and or following a general multi-trauma | NA | Midline tenderness | Se 0.62; Sp 0.92 |
| | | | | | | Palpable midline step | Se 0.14 Sp 1.00 |
| | | | | | | Back bruising | Se 0.69; Sp 0.99 |
| | | | | | | Abnormal neurological signs | Se 0.41; Sp 0.96 |
| **Red flags to screen for vertebral fracture in patients presenting with low-back pain (Williams, 2013)** | | | | | | | |
| PRIMARY STUDIES | | | | | | | |
| First author | Year of publication | Setting(s) of data collection | Number of participants (N) | Population | Reference standard | Index test | Diagnostic accuracy data |
| Roman | 2010 | Secondary | N=1448 | Patients with lumbar-related disorders | Standard radiograph or CT assessing sagittal alignment, vertebral body compression and spinal canal dimensions | No gait abnormality | Se 0.66 (0.49, 0.80); Sp 0.23 (0.21, 0.25); LR + 0.85 (0.68, 1.08); LR- 1.49 (0.95, 2.34) |
| Henschke | 2009 | Primary | N= 1172 | Patients presenting with acute LBP | Imaging studies and specialist view | Sensation change | Se 0.0 (0.0, 0.37); Sp 0.98 (0.97, 0.99); LR+ 3.32 (0.22, 50.86); LR- 0.96 (0.82, 1.13) |
| Reinus | 1998 | Tertiary | N=482 | Patients with back pain presenting to the ED department | Lumbosacral radiographs | Neurological signs | Se 0.05 (0.01, 0.15); Sp 0.92 (0.89, 0.94); LR + 0.69 (0.22, 2.17); LR - 1.03 (0.96, 1.10) |
| Gibson | 1992 | Tertiary | N=225 | Patients presenting to the ED department with lumbar pain of less than 48 hours | Plain radiographs | Neurological signs | Se 0.29 (0.04, 0.71); Sp 0.88 (0.80, 0.94); LR + 2.40 (0.67, 8.70); LR- 0.81 (0.51, 1.30) |
| Patrick | 1983 | Tertiary | N=552 | Patients complaining of LBP presenting to the ED department | Lumbar radiographs | Sensation change | Se 0.03 (0.00, 0.13); Sp 0.98 (0.97, 0.99); LR + 1.42 (0.19, 10.95); LR - 0.99 (0.94, 1.04) |

| | | | | | | | |
|---|---|---|---|---|---|---|---|
| | | | | | | Motor deficit | Se 0.02 (0.00, 0.13); Sp 0.99 (0.98, 1.00); LR + 1.39 (0.08, 25.38); LR - 0.98 (0.96, 1.03) |
| | | | | | | SLR | Se 0.18 (0.07, 0.33); Sp 0.83 (0.79, 0.86); LR + 1.02 (0.51, 2.05); LR - 1.00 (0.86, 1.16) |
| | | | | | | Tenderness | Se 0.73 (0.56, 0.85); Sp 0.59 (0.54, 0.63); LR + 1.76 (1.42, 2.19); LR- 0.47 (0.28, 0.78) |
| | | | | | | Contusion / abrasion | Se 0.85 (0.70, 0.94); Sp 0.97 (0.95, 0.98); LR+ 31.09 (18.25, 52.96); LR - 0.15 (0.07, 0.32) |
| | | | | | | Spasm | LR + 1.47 (0.83, 2.60); LR - 0.90 (0.75, 1.09) |
| Scavone | 1981 | Primary | N=871 | Patients with LBP presenting to the university teaching hospital medical centre | Lumbar radiographs | Sensation change | Se 0.27 (0.12, 0.48); Sp 0.27 (0.12, 0.48); LR+ 2.21 (1.14, 4.27); LR - 0.83 (0.66, 1.05) |
| | | | | | | Motor deficit | Se 0.23 (0.09, 0.44); Sp 0.89 (0.87, 0.91); LR+ 2.19 (1.06, 4.54); LR- 0.86 (0.70, 1.06) |
| | | | | | | Tenderness | Se 0.50 (0.30, 0.70); Sp 0.73 (0.70, 0.76); LR+ 0.70 (0.25, 1.97); LR - 1.11 (0.87, 1.41) |
| | | | | | | Spasm | LR + 1.25 (0.42, 3.70); LR- 0.98 (0.85, 1.12) |

**A systematic review identifies five "red flags" to screen for vertebral fracture in patients with low back pain (Henschke, 2008)**

PRIMARY STUDIES

| First author | Year of publication | Setting(s) of data collection | Number of participants (N) | Population | Reference standard | Index test | Diagnostic accuracy data |
|---|---|---|---|---|---|---|---|
| Holmes | 2003 | Secondary | N=2404 | Patients with back pain presenting to the ED department | Lumbar radiographs | Spinal pain | Se 0.72; Sp 0.35; LR + 1.1 (1.0, 1.2); LR- 0.79 (0.60, 1.01) |
| | | | | | | Tenderness | Se 0.71; Sp 0.28; LR+ 1.0 (0.9, 1.1); LR- 1.04 (0.80, 1.33) |

| | | | | | | | |
|---|---|---|---|---|---|---|---|
| | | | | | | Abnormal neurological exam | Se 0.09; Sp 0.91; LR+ 1.0 (0.6, 1.6); LR- 1.00 (0.94, 1.05) |
| Gestring | 2002 | Secondary | N=71 | Blunt trauma patients requiring CT and x-ray | Lumbar radiographs | Tenderness on palpation | Se 0.40; Sp 0.54; LR+ 0.9 (0.4, 1.7); LR- 1.11 (0.56, 1.72) |
| Terregino | 1995 | Secondary | N=183 | Blunt trauma patients able to be evaluated clinically | Lumbar radiographs | Pain | Se 0.47; Sp 0.88; LR+ 3.9 (1.9, 7.1); LR- 0.60 (0.35, 0.85) |
| | | | | | | Tenderness | Se 0.53; Sp 0.93; LR+ 8.0 (3.8, 15.9); LR- 0.50 (0.28, 0.74) |
| | | | | | | Deformity or neurological11 deficit | Se 0.12; Sp 1.00; LR+46.4 (2.3, 929.1); LR- 0.86 (0.72, 1.04) |
| Scavone | 1981 | Primary | N=871 | Patients with LBP presenting to the university teaching hospital medical centre | Lumbar radiographs | Abnormal deep tendon reflex | Se 0.12; Sp 0.89; LR+ 1.1 (0.4, 2.8); LR- 0.99 (0.79, 1.08) |

Table S23: Diagnostic accuracy of physical examination findings for the diagnosis of spinal malignancy

| **Red flags to screen for malignancy in patients with low-back pain (Henschke, 2013)** | | | | | | | |
|---|---|---|---|---|---|---|---|
| PRIMARY STUDIES | | | | | | | |
| First author | Year of publication | Setting(s) of data collection | Number of participants (N) | Population | Reference standard | Index test | Diagnostic accuracy data |
| Henschke | 2009 | Primary | N= 1172 | Patients presenting with acute LBP | Imaging studies and specialist view | Altered sensation from the trunk down | Se 0 (0.0, 0.0); Sp 0.96 (0.97, 0.99) |
| Khoo | 2003 | Primary | N=1030 | Patients with LBP; hip, leg, sacroiliac pain or trauma; neurological symptoms; possible malignancy; and | Imaging | Neurological symptoms | Se 0.0 (0.0, 0.97); Sp 0.97 (0.95, 0.96) |

| | | | | | | | |
|---|---|---|---|---|---|---|---|
| | | | | inflammatory condition. | | | |
| Deyo | 1988 | Primary | N=1975 | Patients with a primary complaint of LBP of less than 1-month duration | Lumbar radiographs | Fever (> 100°F) | Se 0 (0.0, 0.25); Sp 0.96 (0,97, 0.99) |
| | | | | | | Muscle spasm | Se 0.15 (0.02, 0.45) Sp 0.66 (0.64, 0.68) |
| | | | | | | Neurological symptoms | Se 0 (0.0, 0.26); Sp 0.91 (0.90, 0.92) |
| | | | | | | Spine tenderness | Se 0.15 (0.02, 0.45) Sp 0.60 (0.58, 0.62) |

Table S24: Diagnostic accuracy of physical examination findings for the diagnosis of spinal infection

| **The diagnostic value of red flags in thoracolumbar pain: a systematic review (Maselli, 2020)** | | | | | | | |
|---|---|---|---|---|---|---|---|
| PRIMARY STUDIES | | | | | | | |
| First author | Year of publication | Setting(s) of data collection | Number of participants (N) | Population | Reference standard | Index test | Diagnostic accuracy data |
| Premkumar | 2018 | NA Retrospective study of medical files | N= 9940 | Patients visiting their medical doctor for a chief complaint of LBP | Imaging reports | Fever | Se 0.12; Sp 0.93; LR+ 1.71 (1.04, 2.81); LR- 0.95 (0.89, 1.01) |
| **Low Back Pain in the Emergency Department: Prevalence of Serious Spinal Pathologies and Diagnostic Accuracy of Red Flags (Galliker, 2020)** | | | | | | | |
| PRIMARY STUDIES | | | | | | | |
| First author | Year of publication | Setting(s) of data collection | Number of participants (N) | Population | Reference standard | Index test | Diagnostic accuracy data |
| Davis | 2011 | Primary (ED) | N=55 | Patients who present to ED with spine pain | Imaging | Systolic blood pressure <90 mm Hg | LR+ 9.0 (3.9, 20.7) |

Table S25: Diagnosis accuracy of physical examination findings for the diagnosis of serious spinal pathology

| **The diagnostic value of red flags in thoracolumbar pain: a systematic review (Maselli, 2020)** | | | | | | | |
|---|---|---|---|---|---|---|---|
| PRIMARY STUDIES | | | | | | | |
| First author | Year of publication | Setting(s) of data collection | Number of participants (N) | Population | Reference standard | Index test | Diagnostic accuracy data |
| Shaw | 2020 | Primary (ED) | N= 1000 | Patients presenting to the ED with back pain | NA | Anal tone loss or faecal incontinence | Se 0.09; Sp 0.99; LR+ 6.3 (1.9, 20.8); LR- 0.9 (0.8, 1) |
| | | | | | | Central spine tenderness | Se 0.18; Sp 0.91; LR+ 2.0 (0.9, 4.1); LR- 0.9 (0.8, 1.1) |
| | | | | | | Fever | Se 0.03; Sp 0.99; LR+ 2.0 (0.3, 14.4); LR- 1.0 (0.9, 1.1) |
| | | | | | | Saddle anesthesia | Se 0.09; Sp 0.99; LR+ 11 (3.1, 39.6); LR- 0.9 (0.8,1.0) |
| | | | | | | Sensory level (altered sensation trunk down) | Se 0.6; Sp 0.98; LR+ 0.3 (0.0, 2.0); LR-1.0 (1.0, 1.0) |
| **Low Back Pain in the Emergency Department: Prevalence of Serious Spinal Pathologies and Diagnostic Accuracy of Red Flags (Galliker, 2020)** | | | | | | | |
| PRIMARY STUDIES | | | | | | | |
| First author | Year of publication | Setting(s) of data collection | Number of participants (N) | Population | Reference standard | Index test | Diagnostic accuracy data |
| Thiruganasambandamoorthy | 2014 | Primary (ED) | N=329 | Patients who present to an academic ED with nontraumatic LBP | Blood work, imaging or both | Bladder/suprapubic fullness | LR+ 40.2 (1.6; 979.1) |
| | | | | | | Disturbance of saddle sensation | LR+ 7.0 (1.4, 36.0) |

Table S26: Diagnosis accuracy of diagnostic support tools for the diagnosis of CES

| **The diagnostic value of red flags in thoracolumbar pain: a systematic review (Maselli, 2020)** | | | | | | | |
|---|---|---|---|---|---|---|---|
| PRIMARY STUDIES | | | | | | | |
| First author | Year of publication | Setting(s) of data collection | Number of participants (N) | Population | Reference standard | Index test | Diagnostic accuracy data |
| Premkumar | 2018 | NA Retrospective study of medical files | N= 9940 | Patients visiting their medical doctor for a chief complaint of LBP | Imaging reports | Combination of recent loss of bladder control and recent loss of bowel control | Se 0.08; Sp 0.97; LR+ 3 (1.01, 8.92); LR- 0.94 (0.85, 1.04) |
| Raison | 2014 | Primary (ED) | N=206 | Patients with LBP attending the ED | MRI | Combination of bladder dysfunction/bowel dysfunction and saddle anesthesia | Se 0.27 (0.12, 0.48); Sp 0.92 (0.87, 0.96); LR+ 3.46 |

Table S27: Diagnosis accuracy of diagnostic support tools for the diagnosis of spinal fracture

| **The diagnostic value of red flags in thoracolumbar pain: a systematic review (Maselli, 2020)** | | | | | | | |
|---|---|---|---|---|---|---|---|
| PRIMARY STUDIES | | | | | | | |
| First author | Year of publication | Setting(s) of data collection | Number of participants (N) | Population | Reference standard | Index test | Diagnostic accuracy data |
| Premkumar | 2018 | NA Retrospective study of medical files | N= 9940 | Patients visiting their medical doctor for a chief complaint of LBP | Imaging reports | Combination of trauma and age > 50 years | Se 0.15; Sp 0.94; LR+ 2.54 (2.05, 3.16); LR- 0.90 (0.87, 0.94) |
| | | | | | | Combination of trauma and age > 70 years | Se 0.05; Sp 0.99; LR+ 4.35 (2.92, 6.48); LR- 0.96 (0.94, 0.98) |

| | | | | | | | |
|---|---|---|---|---|---|---|---|
| Enthoven | 2016 | Primary | N=669 | Patients with back pain consulting their GP | Lumbar radiographs | Diagnostic prediction model ≥ 1 positive features (osteoporosis, age of ≥ 75 years, trauma, back pain intensity score ≥ 7 and thoracic pain) | Se 0.88 (0.77, 0.99); Sp 0.42 (0.38, 0.46); LR+ 1.5 (1.3, 1.8); LR- 0.3 (0.1, 0.7) |
| | | | | | | ≥ 2 positive features | Se 0.70 (0.54, 0.85); Sp 0.81 (0.78, 0.84); LR+ 3.6 (2.8, 4.8); LR- 0.4 (0.2, 0.6) |
| | | | | | | ≥ 3 positive features | Se 0.30 (0.15, 0.46); Sp 0.95 (0.93, 0.97); LR+ 5.8 (3.2, 10.8); LR- 0.7 (0.6, 0.9) |
| Henschke | 2009 | Primary | N= 1172 | Patients presenting with acute LBP | Imaging studies and specialist view | Henschke rule 1 sign positive (history of trauma, advanced age, prolonged use of corticosteroids and female gender) | Se 0.88 (0.47, 1.00); Sp 0.50 (0.47, 0.53); LR+ 1.75 (1.34, 2.29); LR- 0.25 (0.04, 1.57) |
| | | | | | | Henschke rule 2 sign positive (2/4) | Se 0.63 (0.24, 0.91); Sp 0.96 (0.95, 0.97); LR+ 15.48 (8.45, 28.36); LR - 0.39 (0.16, 0.96) |
| | | | | | | Henschke rule 3 sign positive (3/4) | Se 0.38 (0.09, 0.76); Sp 1.00 (1.00, 1.00); LR + 906.11 (50.37 - 16299.11); LR - 0.61 (0.36, 1.03) |
| Roman | 2010 | Secondary | N=1448 | Patients with lumbar-related disorders | Standard radiograph or CT assessing sagittal alignment, | 1 of 5 positive tests (age > 52 years, no presence of leg | Se 0.97 (0.89, 0.99); Sp 0.06 (0.06, 0.07); LR+ 1.04 (0.92, 1.1); LR- 0.39 (0.07, 2.1) |

| | | | | | | | |
|---|---|---|---|---|---|---|---|
| | | | | | vertebral body compression and spinal canal dimensions | pain, body mass index ≤22, does not exercise regularly and female gender | |
| | | | | | | 2 of 5 positive tests | Se 0.95 (0.83, 0.99); Sp 0.34 (0.33, 0.34); LR+ 1.4 (1.3, 1.8); LR- 0.16 (0.04, 0.51) |
| | | | | | | 3 of 5 positive tests | Se 0.76 (0.61, 0.87); Sp 0.68 (0.68, 0.69); LR+ 2.5 (1.9, 2.8); LR- 0.34 (0.19, 0.46) |
| | | | | | | 4 of 5 positive tests | Se 0.37 (0.24, 0.51); Sp 0.96 (0.95, 0.97); LR+ 9.6 (3.7, 14.9); LR- 0.65 (0.50, 0.79) |
| | | | | | | 5 of 5 positive tests | Se 0.03 (0.01, 0.08); Sp 0.99 (0.98, 0.99); LR+ 9.3 (1.4, 60.2); LR- 0.97 (0.92, 0.99) |

Table S28: Diagnostic accuracy of diagnostic support tools for the diagnosis of spinal malignancy

| **The diagnostic value of red flags in thoracolumbar pain: a systematic review (Maselli, 2020)** | | | | | | | |
|---|---|---|---|---|---|---|---|
| PRIMARY STUDIES | | | | | | | |
| First author | Year of publication | Setting(s) of data collection | Number of participants (N) | Population | Reference standard | Index test | Diagnostic accuracy data |
| Premkumar | 2018 | NA Retrospective study of medical files | N= 9940 | Patients visiting their medical doctor for a chief complaint of LBP | Imaging reports | Combination of unexplained weight loss and history of malignancy | Se 0.03; Sp 0.99; LR+ 10.25 (3.6, 29.21); LR- 0.98 (0.95,1) |

| Red flags to screen for malignancy in patients with low-back pain (Henschke, 2013) | | | | | | | |
|---|---|---|---|---|---|---|---|
| PRIMARY STUDIES | | | | | | | |
| First author | Year of publication | Setting(s) of data collection | Number of participants (N) | Population | Reference standard | Index test | Diagnostic accuracy data |
| Deyo | 1988 | Primary | N=1975 | Patients with a primary complaint of LBP of less than 1-month duration | Lumbar radiographs | Combination of age greater than 50 years, history of malignancy, unexplained weight loss and failure to improve with conservative therapy | Se 1.00 |

Table S29: Diagnostic accuracy of diagnostic support tools for the diagnosis of spinal infection

| The diagnostic value of red flags in thoracolumbar pain: a systematic review (Maselli, 2020) | | | | | | | |
|---|---|---|---|---|---|---|---|
| PRIMARY STUDIES | | | | | | | |
| First author | Year of publication | Setting(s) of data collection | Number of participants (N) | Population | Reference standard | Index test | Diagnostic accuracy data |
| Premkumar | 2018 | NA Retrospective study of medical files | N= 9940 | Patients visiting their medical doctor for a chief complaint of LBP | Imaging reports | Combination of fever, chills or sweating, and a recent infection | Se 0.08; Sp 0.99; LR+ 13.15 (6.66, 25.97); LR- 0.93 (0.88, 0.98) |
| **Low Back Pain in the Emergency Department: Prevalence of Serious Spinal Pathologies and Diagnostic Accuracy of Red Flags (Galliker, 2020)** | | | | | | | |
| PRIMARY STUDIES | | | | | | | |
| First author | Year of publication | Setting(s) of data collection | Number of participants (N) | Population | Reference standard | Index test | Diagnostic accuracy data |

| | | | | | | | |
|---|---|---|---|---|---|---|---|
| Davis | 2011 | Primary (ED) | N=55 | Patients who present to ED with spine pain | Imaging | Classic triad (Fever ≥38°C, spine pain and neurological deficit) | LR+ 5.7 (1.4, 23.2) |
| Davis | 2004 | Primary (ED) | N=189 | Patients with spinal epidural abscess presenting to the ED | Imaging | Classic triad (Fever ≥38°C, spine pain and neurological deficit) | Se 0.08; Sp 0.99; LR+ 10.0; LR- 0.93 |

Table S30: Diagnostic accuracy of diagnostic support tools for the diagnosis of inflammatory back pain

| **Diagnostic Clinical Prediction Rules for Specific Subtypes of Low Back Pain: A Systematic Review (Haskins, 2015)** | | | | | | | |
|---|---|---|---|---|---|---|---|
| PRIMARY STUDIES | | | | | | | |
| First author | Year of publication | Setting(s) of data collection | Number of participants (N) | Population | Reference standard | Index test | Diagnostic accuracy data |
| Braun | 2013 | Primary | N= 322 | Patients with LBP > 2mo presenting to orthopaedic surgeons, most with ≥ 1 inflammatory back pain symptoms | Diagnosis of axial SpA as determined by a rheumatologist (dichotomous) | (1) age at onset ≤ 35 yrs; (2) alternating buttock pain; (3) improvement with NSAIDs within 48h; (4) waking up in the second half of the night; (5) improvement with movement and not with rest | For 4 or more predictors present: Se 0.48; Sp 0.86; +LR 3.4; -LR 0.6 |
| Chan | 2012 | NA | N=25 | Patients with anterior uveitis | Diagnosis of inflammatory back pain by rheumatologist (dichotomous) | Berlin criteria: (1) morning stiffness, (2) improvement in back pain with exercise but not with rest; (3) awakening because of | For 2 or more predictors present: Se 0.92; Sp 0.67; LRs not reported but calculated to be +LR 2.8 (1.2, 6.3); -LR 0.12 (0.02, 0.79) |

| | | | | | | | |
|---|---|---|---|---|---|---|---|
| | | | | | | back pain in the second half of the night only; (4) alternating buttock pain | |
| Sieper | 2009 | NA | N=648 | Patients with chronic back pain of unknown origin that began before 45 yrs of age | Diagnosis of inflammatory back pain by rheumatologist (dichotomous) | Berlin criteria | For 2 or more predictors present; Se 0.70; Sp 0.81; LRs not reported but approximated to be +LR 3.8 (2.8, 5.0); -LR 0.37 (0.31, 0.43) |
| | | | | | | (1) age at onset < 40 yrs, (2) insidious onset; (3) improvement with exercise; (4) no improvement with rest; (5) pain at night with improvement on getting up | For 4 or more predictors present: Se 0.80; Sp 0.72; LRs not reported, but approximated to be +LR 2.9 (2.3, 3.6); -LR 0.28 (0.23, 0.35) |
| Rudwaleit | 2004 | NA | N=213 | Patients with chronic back pain already diagnosed with either AS or mechanical LBP | Diagnosis of As by a rheumatologist or other specialist prior to the study, using the modified New York criteria (dichotomous) | Berlin criteria | For 2 or more predictors present: Se 0.70 (0.61, 0.78); Sp 0.81 (0.73, 0.87); +LR 3.7 (2.5, 5.6); -LR 0.4 (0.3, 0.5) |

File S31: Medline Search Stragegy

sensitivity or specificity or validity or accuracy or ''predictive value'' or ''predictive validity'' or ''diagnostic accuracy'' or ''diagnostic performance'' or ''diagnostic value'' or ''test performance'' or ''diagnostic utility'' or ''diagnostic ability'' or ''posttest probability'' or ''predictive validity'' or (discriminative N3 (value or validity or performance or ability

((diagnos* or clinical) N3 (test* or examination or evaluation* or assessment* or indicator* or finding* or feature* or history or sign* or symptom*)) or (neurologic* N3 (test* or examination*)) or ''diagnostic test*'' or (physical N3 (examination* or test* or evaluation* or assessment* or sign* or symptom*)) or "sensory test*" or "motor test*" or "muscle test*" or “muscle strength” or evaluation or assessment or imaging or x-rays or radiograph* or ''magnetic resonance imaging'' or MRI or tomograph* or scan or ultraso* or electromyograph* or EMG or electrodiagnos* or (conduction N3 (nerve or motor)) or “electrical stimulation” or “nerve root block*” or (injection* N3 (“nerve root*” or epidural or joint)) or discograph* or scintigraph* or (history N3 (taking or patient or clinical or medical or family)) or ''patient interview'' or (screening N3 (tool* or instrument* or question*)) or questionnaire* or prognosis or prediction or discriminat* or “prognostic screening” or ((prognostic or predictive) N3 (factor* or indicator*)) or “prediction rule*” or ((criteria or model) N3 (diagnostic or classification) or ''yellow flag*'' or ''red flag*'' or biomarker* or (blood N3 (test* or analysis or marker* or factor* or work* or count or parameter*))'' or (laboratory N3 (test* or analysis or profile or marker* or biomarker* or parameter*)) or HLAB27 or HLA-B27 or (inflammat* N3 (marker* or biomarker*)) or (gene* N3 (marker* or biomarker*)) or antibod*

("low back pain" or "lower back pain" or "lumbar radiculopath*" or “lumbosacral radiculopath*” or ("lumbar dis*” N3 hernia*) or ("intervertebral dis*” N3 hernia*) or ''lumbar spinal stenosis'' or ''lumbar pain'' or ''low back ache'' or lombago or lumbalgia or ''lumbar spine'' or ''low back disorder*'' or ''lumbar spinal condition*'' or ''lumbar radicular pain'' or ''lumbar radicular symptom*'' or ''lumbosacral pain'' or sciatica or ''radiating leg pain'' or ''low back related leg pain'' or ''back pain'' or ''back ache'' or ''neurogenic claudication'' or ''intermittent claudication'' or ''axial spondyloarthritis'' or "inflammatory back pain" or “ankylosing spondylitis” or "cauda equina syndrome" or "underlying spinal condition*" or (spin* N3 (fracture* or malignancy or neoplasm* or pathology*)))

AB ( (systematic N3 review*) or guideline* ) OR TI ( (systematic N3 review*) or guideline* ) OR SU ( (systematic N3 review*) or guideline* ) OR PT ( (systematic N3 review*) or guideline* )
